# A purine loop and the primer binding site are critical for the selective encapsidation of mouse mammary tumor virus genomic RNA by Pr77^Gag^

**DOI:** 10.1093/nar/gkab223

**Published:** 2021-04-09

**Authors:** Akhil Chameettachal, Valérie Vivet-Boudou, Fathima Nuzra Nagoor Pitchai, Vineeta N Pillai, Lizna Mohamed Ali, Anjana Krishnan, Serena Bernacchi, Farah Mustafa, Roland Marquet, Tahir A Rizvi

**Affiliations:** Department of Microbiology & Immunology, College of Medicine and Health Sciences (CMHS), United Arab Emirates University (UAEU), Al Ain, United Arab Emirates; Université de Strasbourg, CNRS, Architecture et Réactivité de l’ARN, UPR 9002, Strasbourg, France; Department of Microbiology & Immunology, College of Medicine and Health Sciences (CMHS), United Arab Emirates University (UAEU), Al Ain, United Arab Emirates; Department of Microbiology & Immunology, College of Medicine and Health Sciences (CMHS), United Arab Emirates University (UAEU), Al Ain, United Arab Emirates; Department of Microbiology & Immunology, College of Medicine and Health Sciences (CMHS), United Arab Emirates University (UAEU), Al Ain, United Arab Emirates; Department of Microbiology & Immunology, College of Medicine and Health Sciences (CMHS), United Arab Emirates University (UAEU), Al Ain, United Arab Emirates; Université de Strasbourg, CNRS, Architecture et Réactivité de l’ARN, UPR 9002, Strasbourg, France; Department of Biochemistry, College of Medicine and Health Sciences (CMHS), United Arab Emirates University (UAEU), Al Ain, United Arab Emirates; Zayed Center for Health Sciences, United Arab Emirates University, United Arab Emirates; Université de Strasbourg, CNRS, Architecture et Réactivité de l’ARN, UPR 9002, Strasbourg, France; Department of Microbiology & Immunology, College of Medicine and Health Sciences (CMHS), United Arab Emirates University (UAEU), Al Ain, United Arab Emirates; Zayed Center for Health Sciences, United Arab Emirates University, United Arab Emirates

## Abstract

Retroviral RNA genome (gRNA) harbors *cis*-acting sequences that facilitate its specific packaging from a pool of other viral and cellular RNAs by binding with high-affinity to the viral Gag protein during virus assembly. However, the molecular intricacies involved during selective gRNA packaging are poorly understood. Binding and footprinting assays on mouse mammary tumor virus (MMTV) gRNA with purified Pr77^Gag^ along with in cell gRNA packaging study identified two Pr77^Gag^ binding sites constituting critical, non-redundant packaging signals. These included: a purine loop in a bifurcated stem-loop containing the gRNA dimerization initiation site, and the primer binding site (PBS). Despite these sites being present on both unspliced and spliced RNAs, Pr77^Gag^ specifically bound to unspliced RNA, since only that could adopt the native bifurcated stem–loop structure containing looped purines. These results map minimum structural elements required to initiate MMTV gRNA packaging, distinguishing features that are conserved amongst divergent retroviruses from those perhaps unique to MMTV. Unlike purine-rich motifs frequently associated with packaging signals, direct involvement of PBS in gRNA packaging has not been documented in retroviruses. These results enhance our understanding of retroviral gRNA packaging/assembly, making it not only a target for novel therapeutic interventions, but also development of safer gene therapy vectors.

## Graphical Abstract

**Graphical Abstract:**
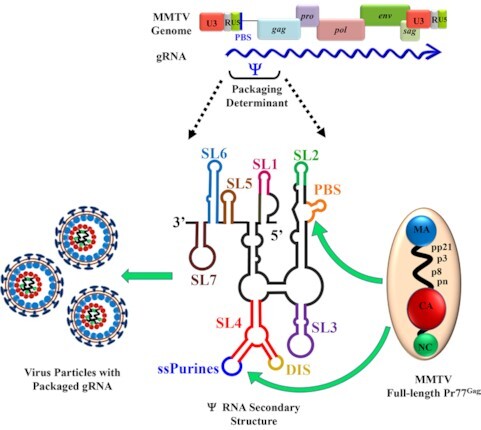
A single stranded purines (ssPurine) loop in a bifurcated stem-loop structure containing the genomic RNA dimerization initiation site (DIS) hairpin, and the primer binding site (PBS) play a crucial role in the packaging of MMTV genomic RNA by recruiting Pr77^Gag^ precursor polyprotein.

## INTRODUCTION

Viruses consist of a protein shell that encloses a genome that can either be DNA or RNA. Viral structural proteins have the ability to specifically recognize their genome and ‘package’ it into the assembling virus particles. They must incorporate their genomes into the virus particles with high specificity to ensure continuity of their life cycle. Different viral groups employ different mechanisms for packaging their genomes selectively and with high fidelity. Retroviruses belong to a special class of viruses that use RNA as their genome (full length unspliced gRNA), which harbors *cis*-acting packaging sequences (called psi, Ψ), that facilitate specific encapsidation of the gRNA (1–8). This is despite the gRNA being in an intense competition with other cellular and viral RNAs for packaging into assembling viral particles formed by the viral protein called ‘Group specific antigen’ (Gag; 1–8). Gag is sufficient by itself to assemble into virus-like particles (VLPs), as has been shown for a number of retroviruses such as human immunodeficiency virus type 1 (HIV-1), mouse mammary tumor virus (MMTV), Mason-Pfizer monkey virus (MPMV) and feline immunodeficiency virus (FIV; 9–13). However, once the viral nucleic acid is present in the cell, Gag can selectively encapsidate the gRNA into the assembling virus particle owing to the presence of *cis*-acting Ψ sequences (14–16). Such selective and faithful packaging has been linked to the presence of high-affinity Gag binding sites within the Ψ on the gRNA (17–21). However, the precise molecular intricacies during selective packaging of gRNA enabling Gag to bind selectively to gRNA over other viral cellular RNAs are still poorly realized.

Similar to retroviruses, ∼8% of the human genome consists of long terminal repeat retrotransposons (LTR retrotransposons) which are retrovirus-like elements also known as endogenous retroviral elements (22). LTR retrotransposon are remnants of retroviruses that have had a profound effect on the evolution of the human genome. They share many functional and structural similarities with retroviruses, such as their basic replication mechanism that includes reverse transcription of gRNA into DNA followed by its integration into the host genome (23,24). Similarly, their unspliced gRNA is used for translation of Gag/Pol proteins as well as for incorporation into the nascent virions (25). Among retroviruses, although a *cis*-packaging mechanism had initially been suggested for human immunodeficiency virus type 2 (HIV-2), it is now generally accepted that packaging of retroviral gRNA takes place independently from Gag translation (26). It has been proposed that, in HIV-1, two pools of gRNA differing by the RNA structure (27,28) and/or its post-transcriptional modifications (29) co-exist, and that only one is packaged. Intracytoplasmic compartmentalization of the Gag and the gRNA and the Rev-dependent nuclear-export mechanism of the gRNA play a critical role in specific packaging of HIV-1 gRNA (30–32). A recent study suggests that the initial interaction with HIV-1 Gag and a fraction of unspliced viral transcripts occurs in the nucleus forming ribonucleoprotein (RNP) complexes. Formation of RNPs with only a fraction of gRNA could be due to the existence of ‘packageable’ and ‘translatable’ gRNA pools (33). Similarly, interaction between gRNA and Rous sarcoma virus (RSV) Gag takes place in the nucleus, at the sites of active viral RNA transcription (34). On the other hand, in *Saccharomyces cerevisiae* Ty1 and Ty3 retrotransposons, the same pool of gRNA undergoes translation and packaging (reviewed in 35). Nevertheless, a distinct feature of all retroviruses and retrotransposons is the packaging of two copies of gRNA that are non-covalently associated into a dimer (4,36,37). Indeed, gRNA dimerization and packaging are intricately related processes (38–41, for reviews see 4,36), even though the molecular mechanism(s) coupling these events remain unknown.

The specificity towards retroviral gRNA recognition by Gag is conferred by Ψ sequences located within the 5′ region of the gRNA (1–3,5–7). Other than the Ψ, additional regions, not directly interacting with Gag may also facilitate gRNA packaging, perhaps by recruiting Gag indirectly (42,43). In Ty3, sequences in both the untranslated regions (UTRs) as well as in the *pol* are necessary for packaging its gRNA, whereas in Ty1, a 144 nucleotide-long region at the 5′ end has been identified as critical for Ty1 gRNA packaging (44–46). However, minimal sequences required for efficient packaging of their gRNA remains largely unclear in both retroviruses and retrotransposons. Nevertheless, it is becoming increasingly clear that specific selection for packaging of retroviral gRNA over cellular and spliced RNAs is a multifaceted phenomenon that occurs in the context of the whole Gag polyprotein (47,48). Despite numerous studies, how the Gag precursor specifically recognizes its gRNA and how gRNA dimerization affects this process remains largely unclear. Therefore, in order to identify general rules that govern retroviral gRNA binding to Gag and packaging, it is important to compare the gRNA packaging process in different retroviruses.

**Figure 1.**
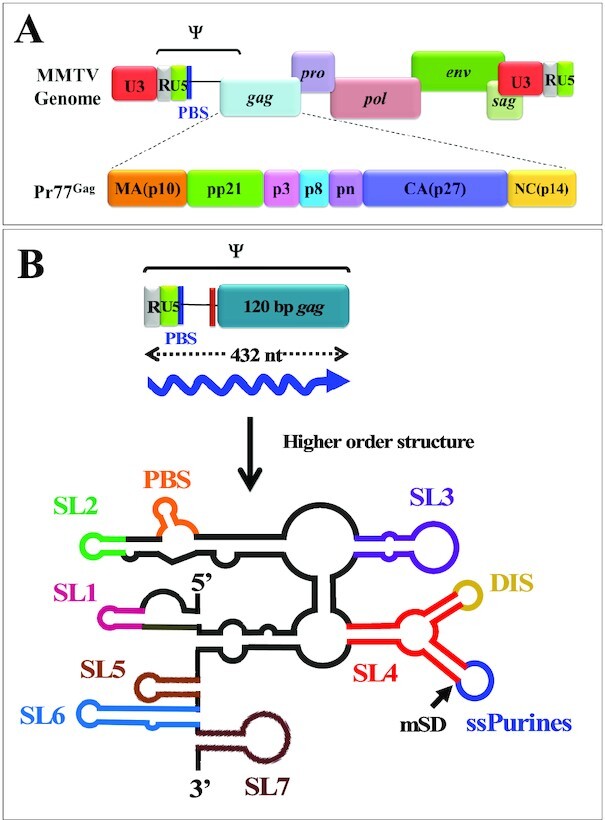
Schematic representation of MMTV genome, organization of different domains of full-length Gag (Pr77^Gag^) and higher order structure of MMTV packaging signal RNA. (**A**) Organization of MMTV full-length genome and domain organization of MMTV Gag precursor protein. (**B**) Schematic representation of RNA secondary structure of MMTV packaging determinants located on the 5′ end of the genome. SL1–7, stem-loops 1–7; PBS, primer binding site; DIS, dimerization initiation site; ssPurines, single stranded purines; mSD, major splice donor.

Here, we studied MMTV, which belongs to the *Betaretrovirus* genus of the *Retroviridae* family and is the etiological agent of breast cancer, and at times T-cell lymphomas, in mice (49–52). The biology of MMTV is being intensely studied to design MMTV-based vectors for human gene therapy due to its ability to infect non-dividing cells, the main target cell population for human gene therapy (53). Unlike most retroviruses including the well-studied lentiviruses, MMTV assembles intracellularly as spherical particles and migrate to the plasma membrane once assembly is completed; after maturation, MMTV displays type B morphology with eccentric cores (54,55). The MMTV gRNA packaging signal comprises the entire 5′ untranslated region (5′ UTR) and the first 120 nucleotides (nts) of the *gag* gene (56–58) and folds into several stem-loops (SLs; 59; Figure 1A and B). Of these, the bifurcated SL4 is of particular interest since one of its apical loops contains a self-complementary sequence that has been shown to act as the dimerization initiation site (DIS), while the other apical loop consists of nine phylogenetically conserved purines (ssPurines; 59; Figure 1B) that have been proposed to be important for MMTV gRNA packaging and may constitute a Gag-binding site (60).

Therefore, to establish whether ssPurines or other sequences within the packaging determinants of MMTV mediate gRNA packaging, possibly by functioning as a Gag binding site(s), we performed a combination of *in vitro* biochemical studies using recombinant full-length Pr77^Gag^ (9) and biologically relevant in cell assays. Our results demonstrate that while ssPurines are required for high affinity Pr77^Gag^ binding, quite strikingly, the primer binding site (PBS) also critically contributes to Pr77^Gag^ binding. Furthermore, our in cell packaging data also established that ssPurines and PBS are not redundant during the MMTV life cycle, as loss of either of these Pr77^Gag^ binding sites ablates gRNA packaging. To the best of our knowledge, these results demonstrate for the first time a direct role of the PBS in retroviral gRNA encapsidation. Future studies will reveal if this feature is unique to MMTV or if it also exists in other retroviruses and/or LTR retrotransposons. Identifying the structural motifs that allow gRNA/Gag interactions is likely to offer opportunities to develop small molecule-based anti-retroviral therapeutic interventions specifically targeting virus assembly especially given the fact that retroviral Gag is the only gene necessary for virus particle formation and gRNA encapsidation for the perpetuation of the virus life cycle.

## MATERIALS AND METHODS

### Nucleotide numbers

Nucleotide numbers refer to nucleotide positions of HYB-MTV, a molecular clone created by Shackleford and Varmus (61).

### Expression and purification of full-length MMTV Pr77^Gag^

Full-length MMTV Gag polypeptide with a C-terminus hexa-histidine tag (Pr77^Gag^-His_6_-tag protein) was expressed, purified and characterized as previously described (9).

### Physical characterization of Pr77^Gag^ by dynamic light scattering (DLS)

Pr77^Gag^ was characterized by DLS in the storage buffer (50 mM Tris–HCl pH 8.0 and 1 M NaCl; 9). Briefly, the intensity of scattered light was measured using a DynaPro Nanostar (100 mW He–Ne laser; Wyatt Technologies) in a 1 μl quartz cuvette (JC-006, Wyatt Technologies) at 20°C. Variations of the diffused light intensity were recorded at microsecond time intervals and the autocorrelation function was derived, allowing the determination of the translational diffusion coefficients (D). Assimilating the proteins in solution to spheres, the diffusion coefficients were related to the hydrodynamic radius (*R*_h_) of the molecules in solution, *via* the Stokes-Einstein equation:

$$\begin{equation*}D\ = \ \frac{{kT}}{{6\pi \mu {R_{\rm h}}}}\end{equation*}$$

in which *k* is the Boltzmann constant, *T* is the temperature and *μ* is the viscosity of the solvent. Before sample acquisition, buffer was filtered through 0.2 μm filters (Millex^®^) and the offset of the solvent was measured for subsequent sample data treatment.

### Cloning, mutagenesis, *in vitro* transcription and RNA purification

Desired mutations in the ssPurines and other regions were introduced through splice overlap extension (SOE) PCR using MMTV subgenomic transfer vector DA024 (57) as the template and OTR 249 and OTR 552 (Supplementary Table S1) as outer sense (S) and antisense (AS) primers, respectively. Mutations were incorporated using inner primers bearing the nucleotide changes to be introduced into the template (Supplementary Table S1). Following two separate first rounds of amplification, the amplified products were mixed to allow annealing at the complementary region harboring the introduced mutation and amplified using the outer primers OTR 249 and OTR 552. The final amplified products containing flanking *Spe*I sites were cleaved and then cloned into the subgenomic transfer vector DA024 that was previously cleaved with the same restriction endonuclease. All clones were confirmed by sequencing.

Clones for *in vitro* transcription were created as previously described (59). Briefly, the 5′ end of the MMTV gRNA corresponding to nts 1–712 (R, +1) was amplified by PCR from the MMTV subgenomic transfer vector DA024 (wild type; WT) or its mutant clones as the template, using primers OTR 984 (S) and OTR 985 (AS) to insert the T7 promoter sequence at its 5′ end. The flanking *Hind*III and *Xma*I sites were used to clone the amplified products into a pUC-based cloning vector (pIC19R; 62). The resulting clones were confirmed by sequencing. Details of the primers used for cloning are provided in the Supplementary Table S1.

Mutations introduced to study in cell biological assays were cloned into the subgenomic transfer vector DA024, referred to as WT. Whereas, for *in vitro* biochemical assays, the same mutations were cloned into a T7 expression plasmid SA35 (referred to as WT and described earlier; 59). To allow ease of understanding, names of the mutations introduced into SA35 were used throughout the manuscript, rather than the actual names of the vectors/clones since the same mutations were introduced into both SA35 and DA024.

For *in vitro* transcription, clones containing the T7 promoter were linearized with *Sma*I, extracted using Roti^®^Aqua for nucleic acid extraction as per manufacturer's instructions (Carl Roth), and resuspended in 30 μl Milli-Q water. Transcription was performed using MEGAscript T7 transcription kit according to manufacturer's instructions (Thermo Fisher Scientific) and following DNase treatment, RNA was extracted, precipitated and resuspended in 500 μl Milli-Q water. The *in vitro* transcribed RNAs were then purified by gel filtration chromatography using a TSKgel G4000SW column (Tosoh Bioscience) with running buffer containing 200 mM sodium acetate and 1% methanol. The eluted fractions under a same peak were pooled, ethanol precipitated, and resuspended in 150 μl of Milli-Q water. The RNA integrity was checked using denaturing polyacrylamide gel electrophoresis (PAGE).

Internally labeled RNAs were prepared by performing *in vitro* transcription in the presence of [α-^32^P]-ATP, as described earlier (41,63–65). Following DNase treatment, the labeled RNAs were electrophoresed on denaturing polyacrylamide gels (6–8%, 8 M urea), bands were excised and extracted in 300 μl of buffer containing 500 mM ammonium acetate, 1 mM EDTA and 0.1% SDS overnight at 4°C. The RNAs were then ethanol precipitated and resuspended in 10 μl Milli-Q water.

### Band-shift and band-shift competition assays

Samples for band-shift assays were prepared by denaturing 50 000 cpm of internally labeled RNA together with 10 nM of the cognate unlabeled RNA (in order to favor RNA dimerization, which might be critical for Pr77^Gag^ binding) and 0.4 μg of yeast tRNA at 90°C for 2 min followed by chilling on ice for 2 min. The denatured RNAs were next allowed to re-fold by incubation in 1× folding buffer (30 mM Tris pH 7.5, 300 mM NaCl, 5 mM MgCl_2_, 5 units of RNasin, 0.01% Triton-X 100, total volume 6 μl) at 37°C for 30 min. Next, increasing concentrations of Pr77^Gag^ were mixed with the refolded RNA (Pr77^Gag^ was diluted in 30 mM Tris pH 7.5, 300 mM NaCl, 5 mM MgCl_2,_ 10 mM DTT, and 0.02 mg/ml BSA in a final volume of 14 μl). The mixture was then incubated at 37°C for 30 min for binding, followed by incubation on ice for 30 min. Samples were separated using 1% agarose gel with electrophoresis performed in TBM buffer (0.5× TB, 0.1mM MgCl_2_) at 150 V for 4 h at 4°C. The gels were fixed in 10% trichloroacetic acid (TCA) for 10 min, dried under vacuum for 1 h and analyzed using a FLA 5000 (Fuji) scanner. Quantitative analysis of the bands was performed using ImageQuant software (20). The Hill slope was identified by applying the values to Hill equation:

$$\begin{equation*}Y\ = \frac{{{B_{{\rm max}}}\ \times \ {X^{h}}}}{{\left( {{K_{\rm d}}^{h} + {X^{h}}} \right)}}\ \end{equation*}$$

using the GraphPad Prism 5 software. *B*_max_ is the maximum specific binding, *K*_d_ is the dissociation constant and *h* is the Hill slope.

**Figure 2.**
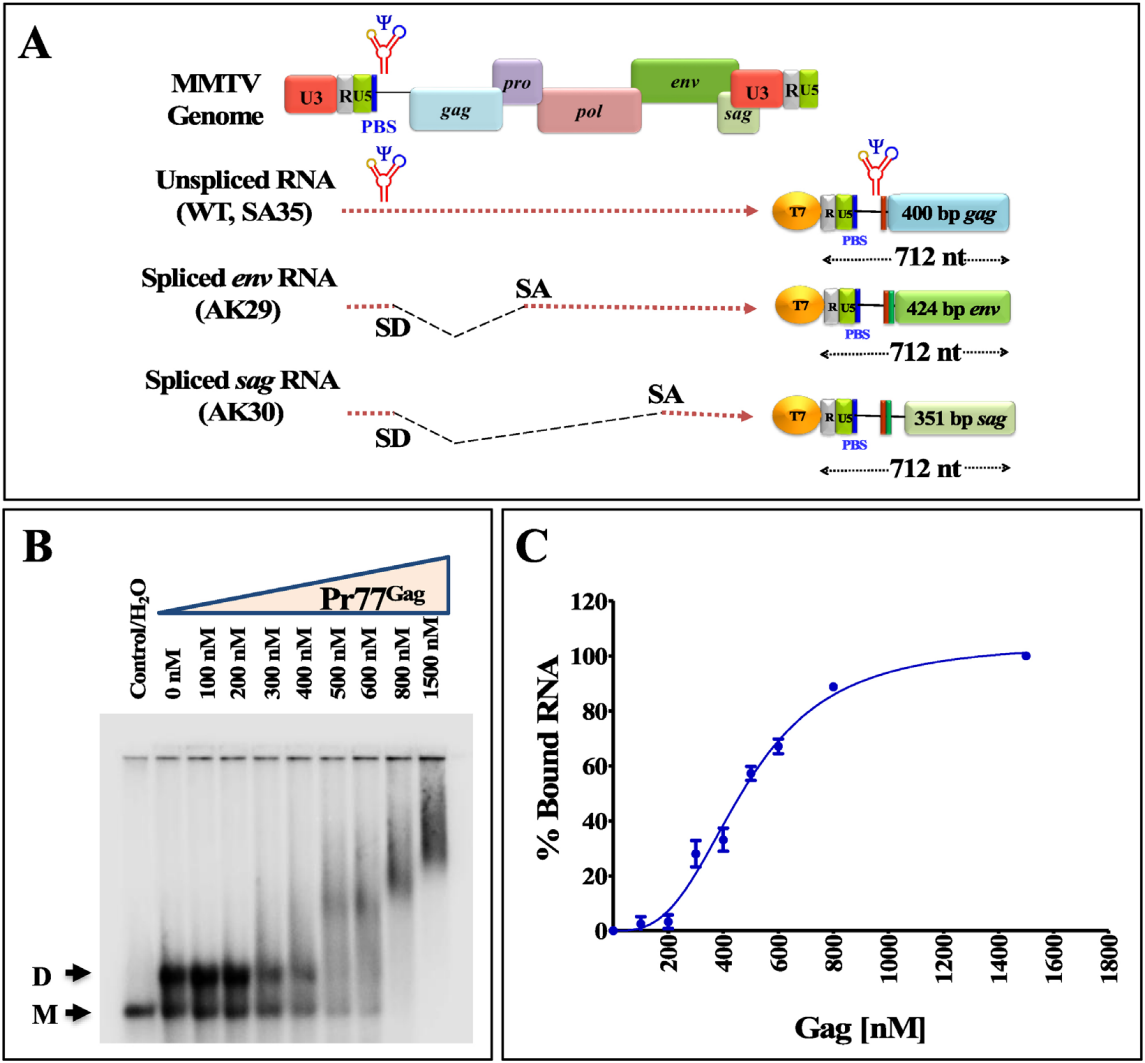
The MMTV packaging signal RNA binds to Pr77^Gag^. (**A**) Scheme of the 712 nts long *in vitro* transcribed unspliced gRNA (SA35), spliced *env* (AK29), and spliced *sag* (AK30) RNAs used for band-shift competition assays. (**B**) Representative gel of a band-shift assay using radiolabeled MMTV gRNA and increasing concentrations of Pr77^Gag^. (**C**) Saturation plot fit to Hill equation based on the quantification of bands obtained in the band-shift assay. Best fit was obtained with plateau = 105 ± 6%, Hill coefficient = 3 ± 0.4 and *K*_d_ = 480 ± 26 nM (mean ± SD); *R*^2^ = 0.99.

For band-shift competition assays, internally labeled WT RNA (25 000 cpm) and 10 nM unlabeled WT RNA (SA35; Figure 2A) along with increasing concentrations of unlabeled competitor RNAs (up to 400 nM) were denatured at 90°C followed by cooling on ice for 2 min. RNAs were refolded by incubation at 37°C for 30 min in folding buffer (30 mM Tris pH 7.5, 300 mM NaCl, 5 mM MgCl_2_, 0.01% Triton X-100, and 5 units RNasin) in a volume of 10 μl. In the meantime, a 20 μM stock of Pr77^Gag^ was diluted in a buffer containing 30 mM Tris pH 7.5, 300 mM NaCl, 5 mM MgCl_2_, 10 mM DTT and 0.02 mg/ml BSA in a volume of 5 μl. The refolded RNAs were then mixed with the diluted protein (final concentration of 800 nM) and incubated at 37°C for 30 min for binding and then on ice for 30 min for stabilization. The reaction mixtures were analyzed on 1% agarose gels with electrophoresis performed in TBM buffer (0.5× TB, 0.1 mM MgCl_2_) at 150 V for 4 h at 4°C. The gels were then fixed and analyzed as described above.

### Filter-binding assay

Samples for filter-binding assays were prepared as described above by denaturing 25 000 cpm of internally labeled RNA, together with 5 nM of the cognate unlabeled RNA and 0.4 μg of yeast tRNA at 90°C for 2 min followed by chilling on ice for 2 min. The denatured RNAs were then incubated at 37°C, mixed with increasing concentrations of Pr77^Gag^ and incubated for an additional 30 min period. The RNA/protein complexes were stabilized at 0°C for 30 min and filtered through a nitrocellulose membrane (0.45 μm, Bio-Rad) using a dot blot apparatus. Membranes were pre-wetted with Tris buffered saline solution (30 mM Tris pH 7.5, 500 mM NaCl) and pre-washed once with 100 ml of buffer (30 mM Tris pH 7.5, 300 mM NaCl). After sample filtration, wells were washed three times with 60 ml of cold buffer (30 mM Tris pH 7.5, 300 mM NaCl), the membranes were removed from the filtration apparatus, and dried on air. The filters were exposed with an Imaging Plate (Fujifilm), scanned with a FLA 5000 (Fuji) scanner and quantified with the ImageQuant software (20).

### Footprinting experiments using hSHAPE in the presence and absence of Pr77^Gag^

High-throughput selective 2′-hydroxyl acylation analyzed by primer extension (hSHAPE) was performed on the WT (SA35) MMTV RNA either in the presence (4 μM) or absence of Pr77^Gag^, as previously described (59). Briefly, the target (100 nM SA35 RNA) and the competitor (400 nM spliced *env* mRNA; AK29) were mixed and denatured at 90°C for 2 min followed by cooling on ice for 2 min. The RNAs were then refolded in a folding buffer (30 mM HEPES–KOH pH 8.0, 300 mM KCl, 5 mM MgCl_2_) along with 5 units of RNasin and 2 μg total yeast tRNA in 10 μl reaction volume for 30 min at 37°C. The 20 μM Pr77^Gag^ stock was diluted in the same folding buffer and mixed with the refolded RNA to a final concentration of 4 μM; RNA in the folding buffer without Gag was used as a control. The mixture was incubated at 37°C for 30 min to allow for protein binding and then stabilized by cooling on ice for 30 min. This was followed by modification of the RNA samples by 100 mM BzCN dissolved in anhydrous DMSO or DMSO alone as a control. The modified and control RNAs were extracted using Roti^®^Aqua, ethanol precipitated, and the pellets were resuspended in 7 μl Milli-Q water.

Finally, the modified and control RNAs were reverse transcribed using VIC-labeled primers (OTR 10 & OTR 14; Supplementary Table S1) in the presence of 1× RT buffer, 0.75 mM of dNTPs and 2 units of AMV RT at 42°C for 20 min and 50°C for 30 min, followed by 60°C for 10 min. For sequencing reactions, reverse transcription was performed using two sets of NED-labeled primers OTR 11 and OTR 15 in a reaction mixture containing 1X RT-buffer, 10 μM ddGTP, 6 μl of G10 (0.15 mM dGTP, 0.6 mM dATP, 0.6 mM dCTP, 0.6 mM dTTP) and 4 units of AMV RT. The cDNAs thus generated from both reactions were mixed together and sequenced using an Applied Biosystems 3130xl genetic analyzer as described earlier (59,60). The electropherograms obtained were analyzed with the software QuShape (66). Normalized SHAPE reactivity data from at least 3–4 independent experiments in the absence of Pr77^Gag^ were used as pseudo-energy constraints to fold the RNA secondary structure of the MMTV packaging signal using the program RNAstructure version 6.1 (67). RNA structures were then drawn using VARNAv3–93 and SHAPE reactivity was incorporated, as described previously (68). The SHAPE reactivity obtained in the presence of 4 μM Pr77^Gag^ was applied onto the RNA structure obtained using reactivity in the absence of Gag. For determination of statistically significant differences between SHAPE reactivities in the absence and presence of Pr77^Gag^, the standard paired, two-tailed Student's *t-*test was performed. Variation in SHAPE reactivity by at least 1.5-fold with a *P*-value ≤0.05 was considered significant.

High-throughput selective 2′-hydroxyl acylation analyzed by primer extension in the absence of Pr77^Gag^ was performed on the spliced RNAs AK29 (*envelope; env*) and AK30 (*superantigen; sag*) as described above except that only yeast tRNA was used as the competitor. For AK29 RNA, Splice_1_1 and Splice_3_1 oligos were used for reverse transcription and Splice_1_2 and Splice_3_2 primers were used for sequencing. Similarly, for AK30 RNA, Splice_1_1 and Splice_2_1 oligos were used for reverse transcription and Splice_1_2 and Splice_2_2 primers were used for sequencing (Supplementary Table S1).

### In cell genetic complementation assay

A previously described three-plasmid genetic complementation assay was used to study the gRNA packaging and RNA transduction efficiencies of the ssPurines mutations (Supplementary Figure S1; 56,57,69). The MMTV subgenomic transfer vector (DA024) containing the minimum *cis*-acting sequences required for RNA packaging, reverse transcription, and integration was used as a source of the RNA to be packaged in the vesicular stomatitis virus envelope glycoprotein (VSV-G) pseudotyped particles produced by JA10 and MD.G (56,57,69). Additionally, DA024 also expresses the *hygromycin B phosphotransferase* gene (*hyg*^r^), which helps to monitor the effect of the mutations introduced into this transfer vector following transduction of the target cells by the viral RNA in a single round of replication assay (Supplementary Figure S1).

Virus particles containing the WT or mutated RNAs were produced in human embryonic kidney (HEK) 293T producer cells and purified as described before (59,60). A fraction of the supernatant was used to infect the human cervical cancer cells (HeLa) to determine the RNA transduction efficiency of the packaged RNA. This was achieved using the single round of replication assay followed by selecting the transduced cells with medium containing 200 μg/ml of hygromycin B (Hyclone). Transduced cells containing the packageable transfer vector RNA expressing the selectable marker resulted in hygromycin resistant colonies that were counted and represented as colony forming units (CFU/ml). The CFU/ml values were then normalized to the transfection efficiency and divided by the WT values to represent the transduction of the mutant RNAs relative to the WT (relative CFU/ml). For determination of statistically significant differences between the WT and the mutations introduced, the standard paired, two-tailed Student's *t-*test was performed.

### Nucleocytoplasmic fractionation, isolation of RNA and cDNA preparation

Nuclear and cytoplasmic fractions were separated from transfected cells as described previously (60). Cellular RNAs from the cytoplasmic fractions and packaged viral RNA from the pelleted viral particles were isolated as described earlier (60). The extracted RNAs were DNase treated and tested for any residual contaminating plasmids by performing conventional PCR using vector-specific primers OTR 1391(S) and OTR 1392 (AS; Supplementary Table S1). DNase free RNA samples were reverse transcribed into cDNA by M-MLV RT (Promega) and amplified to determine the quality of the cDNA samples. A multiplex RT-PCR specific for unspliced β-actin (OTR 582; S and OTR 581; AS; Supplementary Table S1) and 18S rRNA (18S Quantum competimer control, Ambion) was performed to ensure the quality of the nucleocytoplasmic fractionation, as described previously (60). Refer to Supplementary Table S1 for details of primers.

### Quantitative RT-PCR (RT-qPCR) for transfer vector RNA packaging efficiency

Quantitation of the transfer vector RNAs expressed in the cytoplasm and packaged into the pelleted viral particles was accomplished using a Taqman assay and the relative packaging efficiency (RPE) for each mutation was determined as described previously (56,59,60). Once again, the standard paired, two-tailed Student's *t-*test was performed for determination of statistically significant differences in RPE between the WT and the mutations introduced.

### *In vitro* dimerization assay

*In vitro* dimerization assays were performed using RNAs that were *in vitro* transcribed using MEGAscript™ T7 transcription kit (Invitrogen). The *in vitro* transcribed RNAs were purified using MEGAclear™ kit (Invitrogen) and ethanol precipitated as described previously. Briefly, 300 nM RNAs were denatured at 90°C and incubated on ice for 2 min followed by refolding at 37°C for 30 min in either the dimer (30 mM Tris pH 7.5, 300 mM NaCl, 5 mM MgCl_2_) or monomer (30 mM Tris pH 7.5, 300 mM NaCl, 0.1 mM MgCl_2_) buffers. The samples were then electrophoresed in TBM buffer (50 mM Tris, 45 mM boric acid, 0.1 mM MgCl_2_) at 4°C or TBE buffer (50 mM Tris, 45 mM boric acid, 1 mM EDTA) at 25°C using agarose gels prepared in respective buffers. Density of the dimeric and monomeric RNA species was quantitated using ImageQuant software, and dimerization efficiency was calculated by dividing intensity of dimeric RNA band divided by intensity of total RNA bands (i.e., sum of intensities of dimer and monomer bands) and expressed as dimerization relative to the WT for each mutation introduced.

## RESULTS

### Physical characterization of functional Pr77^Gag^ polyprotein

Recently, we reported the purification of recombinant MMTV Pr77^Gag^ polyprotein containing His_6_ tag at the C-terminus expressed in *Escherichia coli* (9). The functionality of the protein was confirmed by the formation of VLPs both in cells and *in vitro*. Moreover, VLPs produced in eukaryotic cells by Pr77^Gag^-His_6_ fusion protein were found to be competent to efficiently package MMTV gRNA (9). The protein preparation was >95% pure based on the ratio of UV absorbance at 260 and 280 nm. DLS experiments revealed that the mean hydrodynamic radius (Rh) based on number distribution was estimated to be ∼6.00 nm, which corresponds to Pr77^Gag^ trimers (Supplementary Figure S2).

### Pr77^Gag^ binds specifically to unspliced MMTV gRNA

First, we asked whether the binding of the Pr77^Gag^ to the WT RNA was specific to the unspliced gRNA or whether it could bind to the spliced RNAs produced by MMTV. This was achieved by testing the differential binding ability of Pr77^Gag^ to unspliced gRNA and spliced *env* and *sag* RNAs using band-shift competition assays. Towards this end, we *in vitro* transcribed the first 712 nts of unspliced gRNA from clone SA35, the spliced *env* and *sag* RNAs were *in vitro* transcribed from clones AK29 and AK30, respectively, while maintaining equal lengths of the RNAs (Figure 2A).

We first analyzed binding of gRNA to increasing concentrations of Pr77^Gag^ (Figure 2B). Shifted bands corresponding to RNA-protein complexes were clearly visible at 500 nM Pr77^Gag^ and above, and the shift increased with increasing protein concentrations, revealing formation of several gRNA-Pr77^Gag^ complexes with different stoichiometry (Figure 2B). After quantification of the gels, the data fit to Hill equation (Figure 2C), and the Hill slope obtained was 3.0 ± 0.4 (mean ± SD) indicating that binding of Pr77^Gag^ to gRNA is cooperative and that at least three Pr77^Gag^ molecules bind per RNA strand. The apparent *K*_d_ obtained was 480 ± 26 nM (mean ± SD).

**Figure 3.**
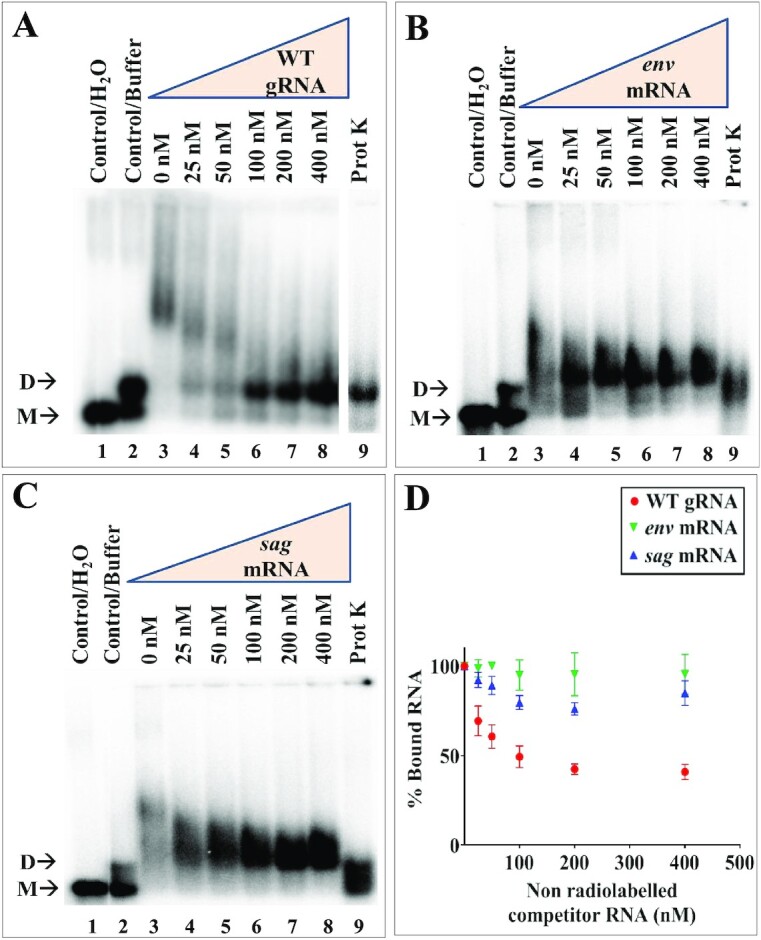
Unspliced genomic and spliced RNAs binds differentially to MMTV Pr77^Gag^. (**A**–**C**) Differential binding specificity of unspliced WT gRNA, spliced *env*, and *sag* RNAs to full-length MMTV Pr77^Gag^ as analyzed by band-shift competition assay. A radiolabeled 712 nts long unspliced WT gRNA and 800 nM MMTV Pr77^Gag^ were incubated with increasing concentrations of unlabeled unspliced WT gRNA, or spliced *env* or *sag* RNAs as competitors, respectively. The last lane in each gel shows the Proteinase K treated reaction mixture containing unspliced WT gRNA, Pr77^Gag^ and 400 nM of respective competitor RNAs. M and D indicate the monomeric and dimeric RNA forms, respectively. (**D**) Quantification of the gels showing the bound fraction of RNA in Gag-RNA complexes. WT, wild type.

Next, band-shift competition experiments were carried out in the presence of 800 nM Pr77^Gag^ since an almost complete shift of radiolabeled unspliced gRNA (<1 nM) was observed at this concentration (Figure 2B). To determine whether the higher mobility-shifted complex was specific to the gRNA, increasing concentrations of unlabeled unspliced gRNA were added to the labeled unspliced gRNA as a competitor (unlabeled SA35; Figure 3A). We observed a gradual reduction and downward shifting of the Gag-bound RNA complex as the concentration of the unlabeled RNA increased, with the reappearance of the dimeric RNA. This indicates that the unlabeled gRNA was able to displace the labeled gRNA from the protein-RNA complex and the displacement was maximum at a concentration of 400 nM of competitor RNA (Figure 3A; lane 8 and Figure 3D). However, when *env* or *sag* spliced RNAs were added as competitors, they could not efficiently compete with the labeled unspliced gRNA for Pr77^Gag^ binding. While the slow-migrating complexes gradually disappeared, an RNA-protein complex migrating immediately above the position of the RNA dimer persisted even at the highest competitor concentrations (Figure 3B and C; lanes 4–8). This is clearly visible in Figure 3D that shows quantitation of the experiments shown in Figure 3A–C. Addition of Proteinase K to Gag-RNA complexes resulted in the re-appearance of labeled dimeric RNA (Figure 3A–C; lane 9), confirming that the shift observed when spliced viral RNAs were used as competitors was indeed due to the protein–RNA interactions (Figure 3B–D; lanes 4–8). Our interpretation of these experiments is that, similarly to what we previously reported for HIV-1 (20), both gRNA and spliced viral RNAs harbor unspecific low affinity binding sites and that, in addition gRNA harbors at least one specific high affinity binding site; spliced viral RNAs are able to displace Pr77^Gag^ from the gRNA low affinity binding sites, but not from the specific high affinity binding site(s).

**Figure 4.**
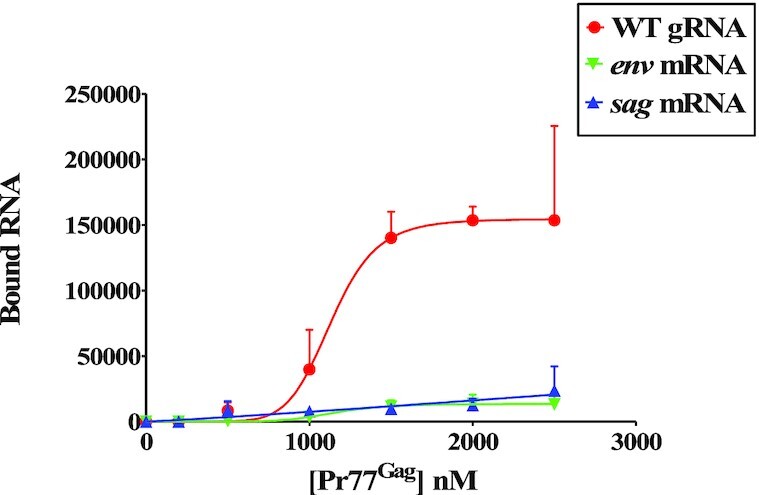
Binding of unspliced genomic and spliced RNAs to MMTV Pr77^Gag^ analyzed by filter-binding assay. The membrane-bound radioactivity of WT gRNA and spliced viral RNAs was quantified at increasing concentrations of MMTV Pr77^Gag^. The experimental data points were fit with the Hill equation. WT, wild type.

To confirm the differential binding of Pr77^Gag^ to unspliced genomic and spliced RNAs by a different technique, direct filter-binding assays, not involving competition between several RNA species, were performed using radiolabeled WT (SA35) or spliced (AK29 and AK30) RNAs in the presence of increasing concentrations of Pr77^Gag^. Compared to WT unspliced gRNA (SA35), the spliced (*env* and *sag*) RNAs showed a drastic reduction in their affinity for Pr77^Gag^, further stressing the existence of high affinity binding site exclusively on the unspliced gRNA (Figure 4).

### Single-stranded purines (ssPurines) are crucial for Pr77^Gag^ binding *in vitro*

**Figure 5.**
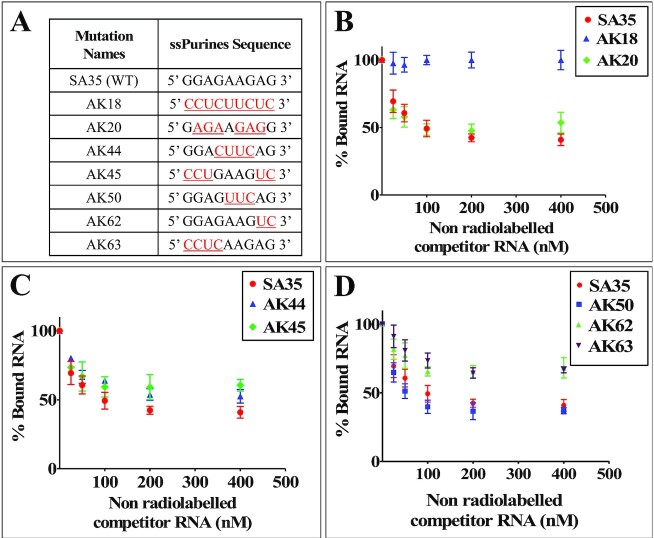
Differential binding abilities of ssPurines and its mutant RNAs to MMTV Pr77^Gag^. (**A**) Nature of mutations introduced in ssPurines that were used in band-shift competition assays. The red and underlined nucleotides represent substitutions introduced. (**B**–**D**) Data generated from quantification of gels analyzing the binding specificity of the listed ssPurine-mutant RNAs to full-length MMTV Pr77^Gag^ analyzed by band-shift competition assays. A radiolabeled 712 nts long unspliced WT gRNA and 800 nM MMTV Pr77^Gag^ were incubated with increasing concentrations of unlabeled competitor mutant RNAs. WT, wild type.

To characterize the role of ssPurines in Pr77^Gag^ binding, a series of mutations were introduced into the ssPurines loop of SL4 (Figure 5A) and the binding specificity of Pr77^Gag^ for each mutation was analyzed by band-shift competition assay, as described above. Competitor RNA transcribed from AK18, in which ssPurines were substituted with complementary pyrimidines (Figure 5A), showed a protein displacement pattern similar to spliced RNAs, indicating that this RNA was unable to displace labeled WT RNA from the Pr77^Gag^-RNA complex (Figure 5B). Such a loss of Pr77^Gag^ binding ability could not be attributed to structural changes as it has recently been shown that substitution of ssPurines with the pyrimidines does not alter the higher order structure of this RNA (60).

In order to determine the effects of the sequence of ssPurines (5′ GGAGAAGAG 3′) on Gag binding, a mutation (AK20) was created in which six of the nine purines were substituted with other purines (5′ GAGAAGAGG 3′; the substituted nucleotides are underlined; Figure 5A). In mutation AK50, the internal AAG motif within the ssPurines was substituted with UUC (5′ GGAGUUCAG 3′; substituted nucleotides are underlined; Figure 5A). Band-shift competition assays performed using mutant RNAs (AK20 & AK50) showed binding ability of Pr77^Gag^ similar to WT RNA (Figure 5B and D). Substitution of internal GAAG purines with CUUC in AK44 (5′ GGACUUCAG 3′; Figure 5A) or maintaining GAAG while substituting the flanking sequences in ssPurines in AK45 (5′ CCUGAAGUC 3′; Figure 5A), showed a slightly reduced ability to compete with WT RNA for Pr77^Gag^ binding (Figure 5C). In contrast, substitution of the first four purines (AK63, 5′ CCUCAAGAG 3′; Figure 5A) or the last two purines (AK62, 5′ GGAGAAGUC 3′; Figure 5A) with complementary pyrimidines, resulted in RNA substrates with lower ability to displace the Gag protein from the WT RNA, albeit some displacement was still observed (Figure 5D).

**Figure 6.**
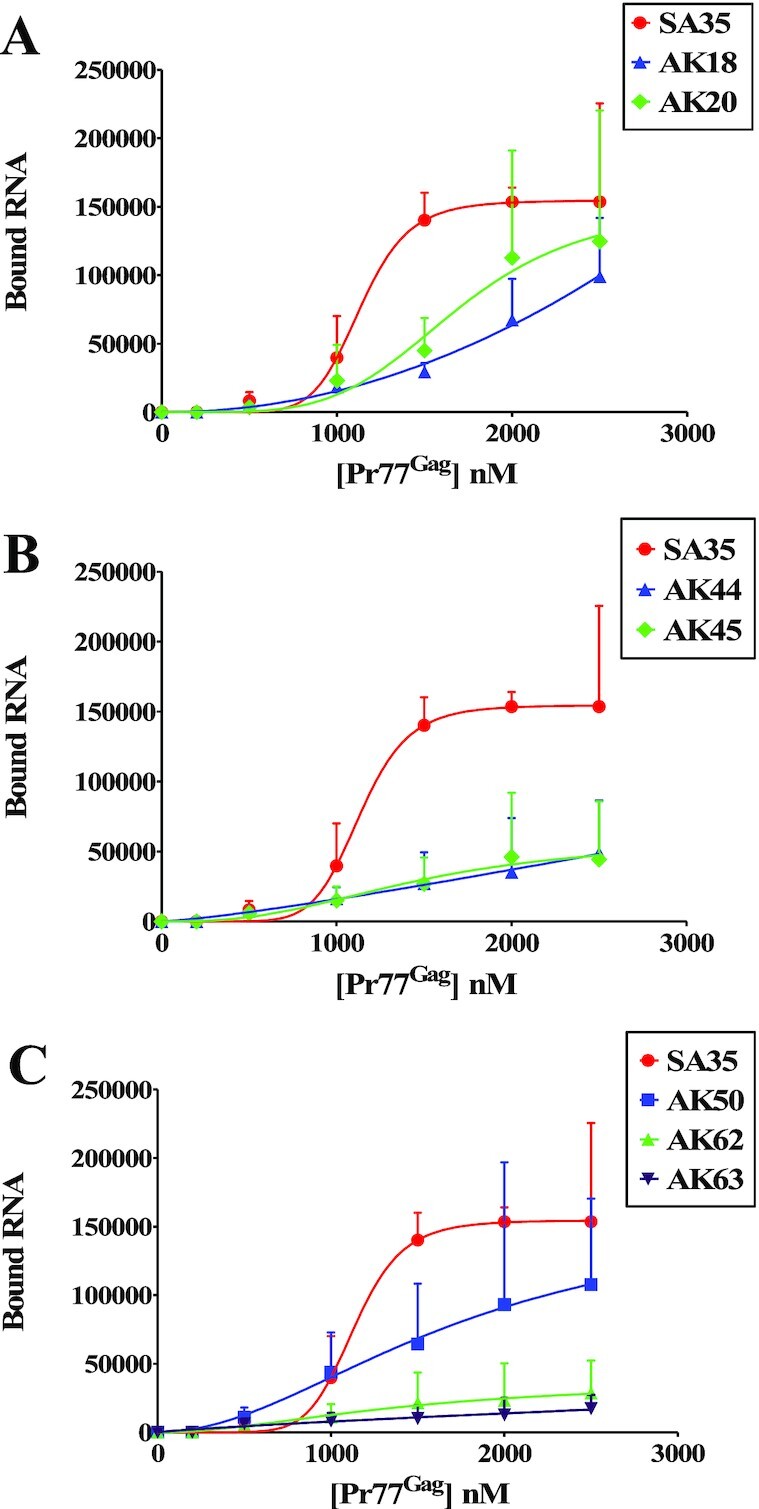
Binding of ssPurines and its mutant RNAs to MMTV Pr77^Gag^ analyzed by filter-binding assay. The membrane-bound radioactivity of WT gRNA and selected mutant RNAs was quantified at increasing concentrations of MMTV Pr77^Gag^. The experimental data points were fit with the Hill equation. WT, wild type.

To further demonstrate the *in vitro* binding of Pr77^Gag^ to ssPurines in a non-competitive experimental setup, we performed filter-binding assay using radiolabeled WT (SA35; 5′ GGAGAAGAG 3′) or ssPurine mutant RNAs (Figure 5A). The mutations AK20 (containing six purine substitutions with other purines; 5′ GAGAAGAGG 3′) and AK50 (5′ GGAGUUCAG 3′; three purines substituted with pyrimidines) revealed slightly reduced levels of binding to Gag compared to the WT gRNA (Figure 6A and C). Unexpectedly, mutation AK18 (ssPurines substituted with complementary pyrimidines) showed moderate binding affinity to Pr77^Gag^ (Figure 6A), in contrast to our competition assay which revealed that AK18 is a non-competitor compared to WT gRNA (Figure 5B). Such a discrepancy could be due to the differences in the two experimental conditions employed. On the other hand, mutations AK44 (5′ GGACUUCAG 3′) and AK45 (5′ CCUGAAGUC 3′) containing partial substitution of purines to pyrimidines showed a slightly reduced competition to WT gRNA (Figure 5C), and moderate affinity to Pr77^Gag^ in filter-biding assays (Figure 6B). Substitution mutations AK62 (5′ GGAGAAGUC 3′) and AK63 (AK63, 5′ CCUCAAGAG 3′) exhibited a drastically reduced ability to bind to Pr77^Gag^ (Figure 6C), further validating results of the competition assays (Figure 5D). This confirmed that the two stretches of purines: (i) GGAG at the 5′ end and (ii) AG at the 3′ end of ssPurines are important for RNA–Gag interactions.

To ensure that the introduced mutations in ssPurines somehow did not compromise the ability of the mutant RNAs to dimerize (which in turn may result in poor affinity for Gag), *in vitro* dimerization assays were performed on three crucial mutations (AK18, AK62 and AK63). The results obtained revealed that all the three mutations maintained dimerization abilities similar to that of the WT (SA35) RNA (Supplementary Figure S3).

Together, these results validate our hypothesis that ssPurines (5′ GGAGAAGAG 3′) act as a high affinity binding site for Pr77^Gag^ and the results observed were not due to effects of the introduced mutations on gRNA dimerization. Notably, the 5′ GGAG and 3′ AG nucleotides are important for retaining high affinity binding (mutations AK50, AK62 and AK63, Figures 5D and 6C), while the remaining purines (AAG; AK50; Figures 5D and 6C) are less critical. Furthermore, comparison of the SA35 (WT) and AK20 sequences suggest that the identity of the first and last nucleotides in the SL4 ssPurine loop is a key determinant for Pr77^Gag^ binding (Figures 5B and 6A).

### ssPurines in the spliced RNAs are base-paired

**Figure 7.**
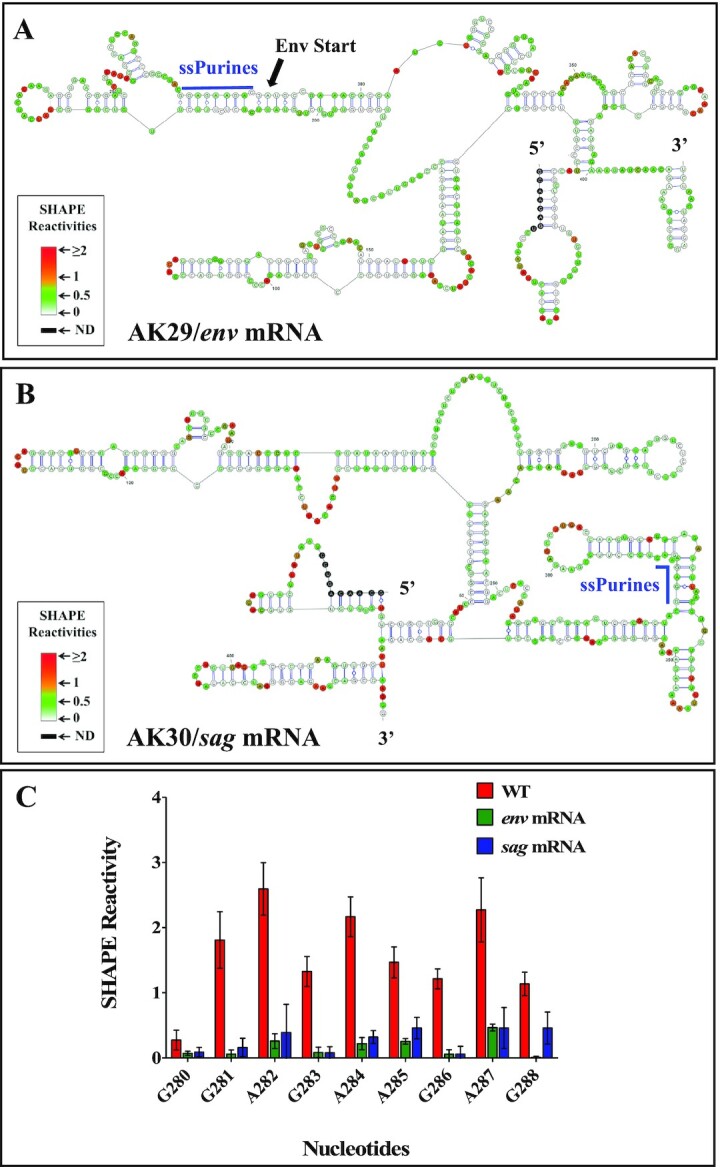
SHAPE-validated RNA secondary structure of first 712 nts of: (**A**) *env* (AK29), (**B**) *sag* (AK30) RNAs showing that ssPurines base-pairs with other sequences following RNA splicing and (**C**) Graphical representation showing SHAPE reactivities of ssPurine region in the WT gRNA, spliced *env* and *sag* RNAs. SHAPE data were used as constraints for all structures. WT, wild type.

The ssPurines are present upstream of the mSD and thus present in both the unspliced (SA35) as well as spliced mRNAs (*env*; AK29 and *sag*; AK30; Figure 2A). However, *in vitro* binding assays performed on these spliced RNAs showed poor competition efficiency/binding capacity to Pr77^Gag^ compared to unspliced RNA (Figures 3 and 4). These were intriguing results that one could rationalize if the ssPurines in the spliced RNAs were no longer present in an unpaired conformation. Therefore, to test such a possibility, we performed hSHAPE experiments on the gRNA and on both *env* (AK29) and *sag* (AK30) spliced RNAs to elucidate the secondary structure of these RNAs. Results of these experiments revealed that the ssPurines in both the *env* and *sag* spliced RNAs were present in a base-paired state with neighboring sequences and therefore unavailable for Pr77^Gag^ binding (Figure 7 and Supplementary Table S2). This suggests a possible mechanism for the preferential binding of unspliced RNAs over spliced RNAs, governing specific selection of full-length gRNA during the packaging process.

### Pr77^Gag^ attenuates the reactivity of ssPurines towards SHAPE reagents

To further analyze binding of Pr77^Gag^, hSHAPE was performed using the benzoyl cyanide reagent (BzCN) and WT ψ-containing RNA, both in the absence as well as presence of Pr77^Gag^. The nucleotides showing reduced SHAPE reactivity in the presence of the protein correspond to protein binding site(s), as Pr77^Gag^ binding protects these nucleotides from modification by BzCN. To minimize non-specific binding of Pr77^Gag^ to WT RNA, hSHAPE was performed in the presence of excess spliced *env* mRNA (AK29; 4-fold excess molar concentration). Pr77^Gag^ was used at a concentration of 4 μM (i.e. about 10-fold higher concentration than the *K*_d_ value) to ensure complete binding of RNA to Pr77^Gag^. Under the conditions (protein and competitor RNA concentrations) used for footprinting, the band-shift assay showed formation of only a single complex, indicating that we were looking only at the high affinity binding site in the SHAPE footprinting experiments (Supplementary Figure S4).

**Figure 8.**
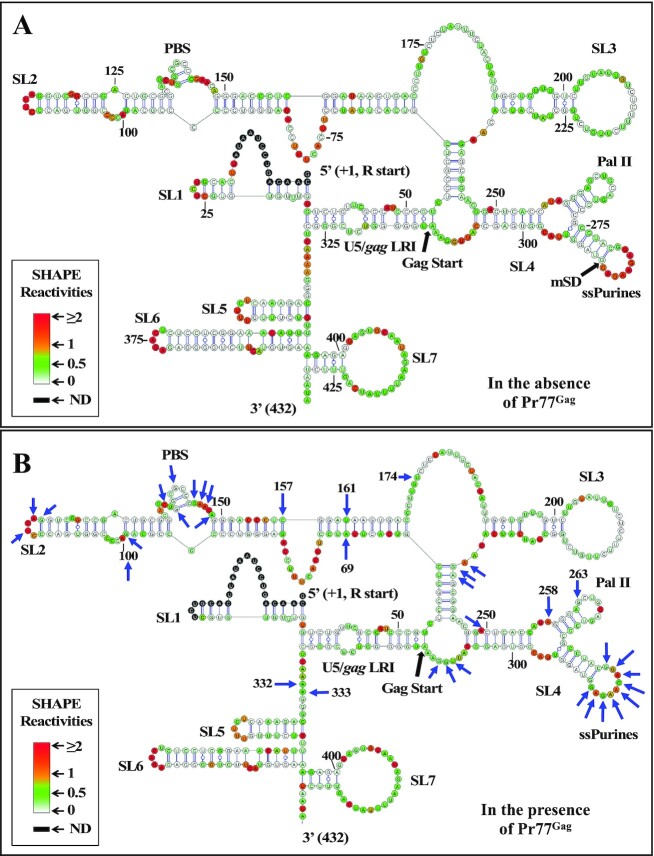
SHAPE-validated secondary structure and footprints of Pr77^Gag^ on MMTV packaging signal RNA. **(A)** SHAPE validated secondary structure of MMTV packaging signal RNA obtained when hSHAPE was conducted in the absence of MMTV Pr77^Gag^. **(B)** The reactivities obtained in presence of Gag were represented on the secondary structure based on the SHAPE reactivities in the absence of Gag. All nucleotides marked by arrows showed a 1.5- to 2-fold reduction in SHAPE reactivities with statistical significance (*P*-value ≤ 0.05; tested by paired, two-tailed Student's *t*-test).

In the absence of Pr77^Gag^, we obtained the higher order structure of MMTV ψ-RNA (Figure 8A) that is identical to the one that has been reported earlier (59,60) except for some minor changes in SL5, SL6 and SL7 located at the 3′ end of the packaging signal RNA. SHAPE reactivities obtained in the presence of Pr77^Gag^ were then plotted on the structure obtained using reactivities without Pr77^Gag^ in order to identify the reactivity changes for each nucleotide (Figure 8B). High-throughput SHAPE analysis in the presence of Pr77^Gag^ resulted in 1.5- to 2-fold reduction in reactivity of all ssPurines (*P*-values ≤ 0.05; Supplementary Figure S5A and Supplementary Table S3), indicating that Pr77^Gag^ directly binds to the ssPurines, an observation in agreement with the band-shift competition and filter-binding data.

### Pr77^Gag^ attenuates reactivities of nucleotides other than ssPurines

Besides ssPurines, SHAPE analysis of MMTV WT RNA conducted with and without Pr77^Gag^ revealed attenuated reactivity for a few other nucleotides (*P*-values ≤ 0.05, Figure 8B). These nucleotides are dispersed as discontinuous short stretches throughout the packaging signal RNA, such as the apical part of SL2 (nts G99, A100, U113, A115 and G116; Figure 8B & Supplementary Figure S5B), the PBS region (nts C135, G137, G140, G144, A145, A146 and A148; Figure 8B and Supplementary Figure S5C), the basal part of SL3 (nts A237, G238 and A239; Figure 8B and Supplementary Figure S5D), the bulge downstream of SL4 (nts U308, G309, and G310; Figure 8B and Supplementary Figure S5E), and the unpaired region upstream of SL5 (nts A332 and A333; Figure 8B and Supplementary Figure S5E). In addition, some nucleotides that are distantly located from these regions, A69 and its complementary pair U161, C157 (in SL2), U174 (bulge downstream of SL2), A249, A258 and G263 (in SL4) also showed attenuated SHAPE reactivity (Figure 8B). However, at this point, we cannot ascertain whether these attenuated SHAPE reactivities are due to direct Pr77^Gag^ binding or to Gag induced structural changes in these regions. The role of these nucleotides in gRNA packaging is analyzed below.

### Role of ssPurines during in cell packaging and transduction of ψ-containing RNAs

**Figure 9.**
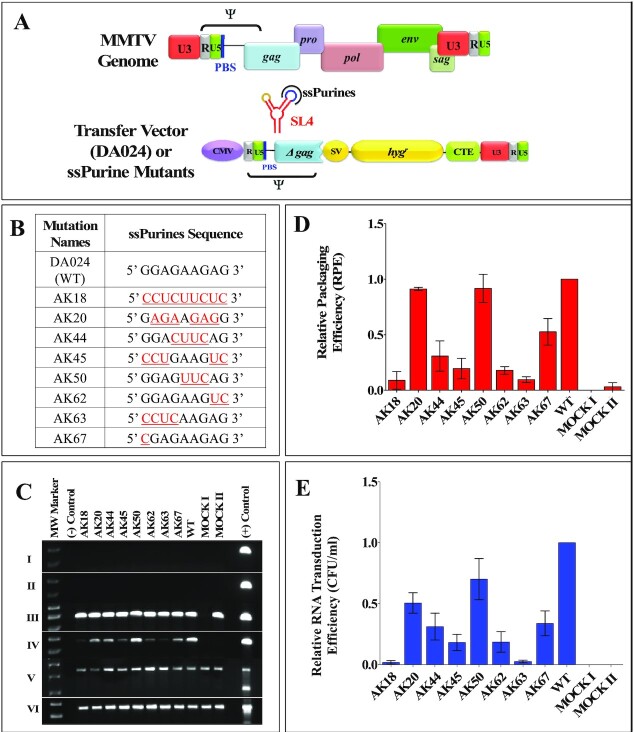
Role of ssPurines in MMTV gRNA packaging and transduction efficiencies. (**A**) Schematic representation of the MMTV genome and MMTV-based WT transfer vector, DA024. (**B**) Nature of the mutations introduced in the ssPurines and cloned into the background of subgenomic transfer vector DA024. The red and underlined nucleotides represent substitutions introduced. (**C**) PCR amplifications of the DNase-treated cytoplasmic (panel I) and viral (panel II) RNAs using virus-specific primers (169 bp). Panels III and IV show PCR amplifications of the cytoplasmic and viral cDNAs using virus-specific primers (169 bp). Multiplex amplifications conducted on cDNAs in the presence of primers/competimer for 18S rRNA (324 bp) and unspliced β-actin mRNA (200 bp; panel V). Panel VI shows PCR amplification of cytoplasmic cDNAs using spliced β-actin mRNA primers (249 bp). (**D**) Relative packaging efficiencies (RPE) of mutant transfer vector RNAs. (**E**) Relative RNA transduction efficiencies of the packaged mutant RNA represented as hygromycin resistant (hyg^r^) colony forming units per ml (CFU/ml). Mock I contain only the packaging construct (without transfer vector) and Mock II has only transfer vector and no packaging construct.

In order to assess the biological significance of the ssPurines mutations tested in band-shift competition and filter-binding assays (Figure 5A) and which showed attenuated SHAPE reactivity (Figure 8), same mutations were cloned into the subgenomic transfer vector (DA024; Figure 9A) and are listed in Figure 9B. These mutations were tested in a biologically relevant single round of replication assay developed in our laboratory (57) to investigate their effect on RNA packaging and transduction efficiencies of the packaged RNAs. The rationale of three-plasmid genetic complementation assay is depicted in Supplementary Figure S1 and described in the Materials and Methods section. Following transfection, the pseudotyped MMTV particles generated were analyzed for the amount of transfer vector RNA packaged into the virions to determine the biological significance of the mutations introduced in ssPurines. In parallel, a portion of the virus particles were used to infect HeLa cells to monitor their ability to transduce the packaged RNA into the target cells by the appearance of hygromycin resistant colonies.

The packaging efficiency of the RNAs was calculated by determining the amount of RNA packaged into the virus particles normalized to the amount of RNA expressed in the cytoplasm. Thus, RNAs from cytoplasmic fractions and pelleted viral particles were extracted and DNase treated to remove any contaminating plasmid DNA. This was confirmed by amplification of the DNase-treated cytoplasmic and viral RNAs using transfer vector-specific primers and showing no amplification, thus confirming that RNA preparations were free of any contaminating plasmids (Figure 9C, Panels I and II respectively). The DNased-RNAs were then converted into cDNAs and the expression of the transfer vector RNAs (WT and mutant RNAs) in the cytoplasm and the packaged RNA in the virus particles were analyzed by RT-PCR (Figure 9C, Panels III and IV). The absence of unspliced β-actin mRNA and presence of 18S rRNA (Figure 9C, Panel V) in the cytoplasmic fractions by RT-PCR ensured that the nuclear membrane integrity was maintained during nucleocytoplasmic fractionation. This was important to demonstrate since the relative packaging efficiency (RPE) is calculated in relation to the stably expressed RNAs that are efficiently exported from the nucleus to the cytoplasm (56,59,60). After having taken into account all the necessary controls described above, the amount of transfer vector RNAs (WT or mutant RNAs) expressed in the cytoplasm and packaged RNA in the pelleted virus particles were quantitated using RT-qPCR (Figure 9D; 56,59,60).

A complete substitution of ssPurines with complementary pyrimidines (AK18) resulted in a drastic reduction in the packaging efficiency (RPE = 0.089 ± 0.079; Figure 9D) of AK18 compared to the WT, DA024. On the other hand, packaging efficiency of the mutation AK20 (containing a reverse sequence of ssPurines, 5′ GGAGAAGAG 3′ to 5′ GAGAAGAGG 3′) was similar to wild type levels (Figure 9D). These results demonstrated that ssPurines in either orientation was necessary for optimal packaging of MMTV gRNA. The mutation in which the GGA at the 5′ end and AG at the 3′ end of ssPurines were substituted with CCU and UC, respectively (5′ CCUGAAGUC 3′; AK45) showed nearly 80% reduction in packageability of the mutant RNA (RPE = 0.195 ± 0.091; Figure 9D), which was in close agreement with Pr77^Gag^ binding ability of this mutation (AK45; Figures 5C and 6B). This further suggested that either both or one of the flanking sides of ssPurines plays an important role in binding to Pr77^Gag^. The packaging efficiency of mutation in which the central GAAG of ssPurines was substituted with CUUC (5′ GGACUUCAG3′; AK44) was found to be slightly increased when compared to AK45 containing flanking substitutions (5′ CCUGAAGUC 3′; RPE = 0.308 ± 0.135; Figure 9D). Interestingly, the mutation in which the internal AAG was substituted with UUC (5′ GGAGUUCAG 3′; AK50) showed packaging efficiency comparable to WT (RPE = 1.00 ± 0.4; Figure 9D).

Next, to investigate the importance of the nucleotides present at the flanks of ssPurines, individual mutations specific to the GGAG at the 5′ end (5′ CCUCAAGAG 3′; AK63) and AG at 3′ end of ssPurines (5′ GGAGAAGUC 3′; AK62; Figure 9B) were tested for the direct effect of these regions on the RNA packaging process. The RPE of AK63 and AK62 revealed 80 to 90 percent reduced packaging efficiency (AK63, RPE = 0.095 ± 0.026 and AK62, RPE = 0.178 ± 0.034; Figure 9D). These results are consistent with Pr77^Gag^ binding assays, which showed reduced ability of these mutant RNAs to bind Gag (AK63 & AK62; Figures 5D and 6C). Finally, based on our SHAPE footprinting data, G280 (the first G in ssPurines) showed minimal SHAPE reactivity both in the absence as well as presence of Pr77^Gag^ (0.27 versus 0.18, respectively, *P* ≤ 0.05; Figure 8 and Supplementary Figure S5A). Analysis of the RPE of the substitution mutation (5′ CGAGAAGAG 3′; AK67; Figure 9B) revealed about 50% reduction in the packaging efficiency when compared to the WT (DA024).

Finally, the relative RNA transduction efficiency for each of the mutation introduced was calculated by counting the number of hygromycin resistant colonies (CFU/ml) that appeared when viral particles containing these mutants RNAs were used to transduce target cells. For all the introduced mutations, relative transduction of the packaged RNA corroborated well with the relative packaging efficiency except for mutation AK20 (containing a reverse sequence of ssPurines, 5′ GGAGAAGAG 3′ to 5′ GAGAAGAGG 3′). Contrary to our expectations, RNA transduction efficiency of AK20 RNA was observed to be only 50% (compared to WT; DA024) despite the fact that the RNA containing this mutation was packaged almost to the WT (DA024) levels (compare Figure 9D with E). This suggests that while the AK20 mutation did not affect RNA packaging, it may have had some inadvertent effect on subsequent steps of RNA propagation, such as reverse transcription and/or integration. Overall, results from these single round replication assays indicate that ssPurines are crucial for RNA packaging and the 5′ GGAG and the 3′ AG nucleotides are required for the optimal packaging of MMTV gRNA.

### Nucleotides in the PBS region play a crucial role in RNA packaging

**Figure 10.**
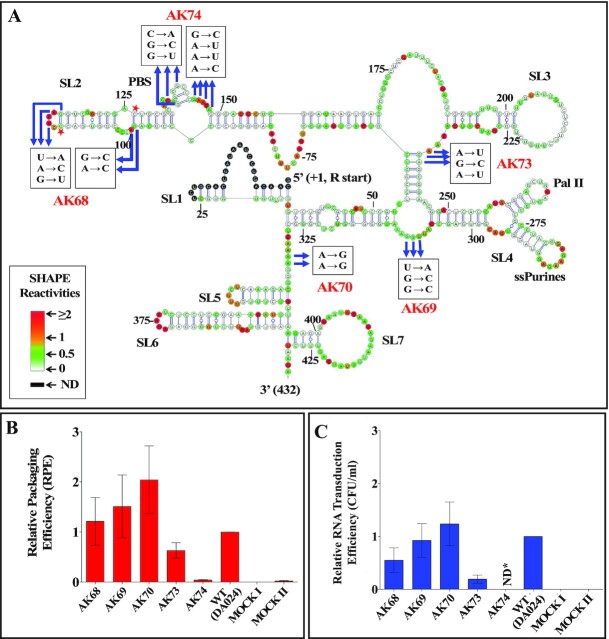
Role of nucleotides outside ssPurines that showed attenuation of SHAPE reactivities in the presence of MMTV Pr77^Gag^ on MMTV gRNA packaging and transduction efficiencies. (**A**) Nature of mutations introduced in the SHAPE-validated structure of MMTV gRNA showing attenuation of SHAPE reactivities in the presence of MMTV Gag. The nucleotides showing attenuation other than ssPurines are marked by arrows. Note that this figure and Figure 8B are the same and has been reproduced to depict the SHAPE reactivities and the locations of the nucleotides identified for introducing mutations. Mutations were introduced in these nucleotides (boxed) to test their role in RNA packaging and transduction. The red star marked nucleotides are the compensatory mutated nucleotides (C111 to A and C126 to G) in AK68 in order to maintain the secondary structure. (**B**) Relative packaging efficiency (RPE) of mutant transfer vector RNAs. (**C**) Relative RNA transduction efficiencies (tested using single round of replication assays) of the packaged mutant RNAs represented as hygromycin resistant (Hyg^r^) colony forming unit per ml (CFU/ml). Mock I contain only the packaging construct (without transfer vector) and Mock II has only transfer vector and no packaging construct. *ND; not determined.

After having confirmed that the ssPurines are required for MMTV gRNA packaging, it was important to determine the role of other nucleotides that have shown attenuation of SHAPE reactivity in our footprinting assays. It was particularly important to ascertain whether the reduced SHAPE reactivity was due to direct Pr77^Gag^ binding or to Pr77^Gag^-induced RNA conformational changes. Toward this end, we grouped those nucleotides into five regions and groups of substitution mutations were introduced to test their RNA packaging and transduction abilities using the single round of replication assays. The details and/or nature of these substitution mutations (AK68, AK69, AK70, AK73 and AK74) is schematically shown in Figure 10A.

After performing the same necessary controls as described for the other mutations (Supplementary Figure S6), the packaging efficiency of these mutations was determined. Mutations in the apical part of SL2 (AK68), bulge downstream of SL4 (AK69), and the unpaired region upstream of SL5 (AK70) did not detrimentally affect RNA packaging efficiency; rather, these mutations showed increased RNA packaging (Figure 10B, AK68; RPE = 1.21 ± 0.47, AK69; RPE = 1.50 ± 0.62, AK70; RPE = 2.02 ± 0.67). Transduction efficiencies for these mutations revealed a similar pattern except for mutation AK68 (Figure 10C). The mutation AK68 showed almost 50% reduction in its ability to transduce the packaged RNA (Relative CFU/ml = 0.54 ± 0.23; compare Figure 10B with C). On the other hand, substitution of the three nucleotides at the bottom part of SL3 in mutation AK73 showed an intermediate effect, resulting in 40% reduction in RNA packaging efficiency compared to WT (AK73, RPE = 0.63 ± 0.153), and a correspondingly reduced transduction ability (CFU/ml = 0.19 ± 0.076; compare Figure 10B with C). On the contrary, substitution of 7 nucleotides in the PBS region within SL2 (AK74) resulted in a drastic reduction in RNA packaging (96% reduction, RPE = 0.043 ± 0.005; Figure 10B), revealing the importance of this region to MMTV gRNA packaging. Transduction of the packaged RNA for this mutation (AK74) was not investigated since mutations in the PBS limits the ability of the packaged RNA to reverse transcribe which is a must for the transduction read out assay (Supplementary Figure S1). Together, these results demonstrate that the nucleotides in the first three regions tested (apical part of SL2, bulge downstream of SL4, and the unpaired region upstream of SL5) are not important for RNA packaging, nucleotides in the bulge downstream of SL3 have some moderate effect, while nucleotides in the PBS region have the most drastic effect on RNA packaging amongst the regions tested.

**Figure 11.**
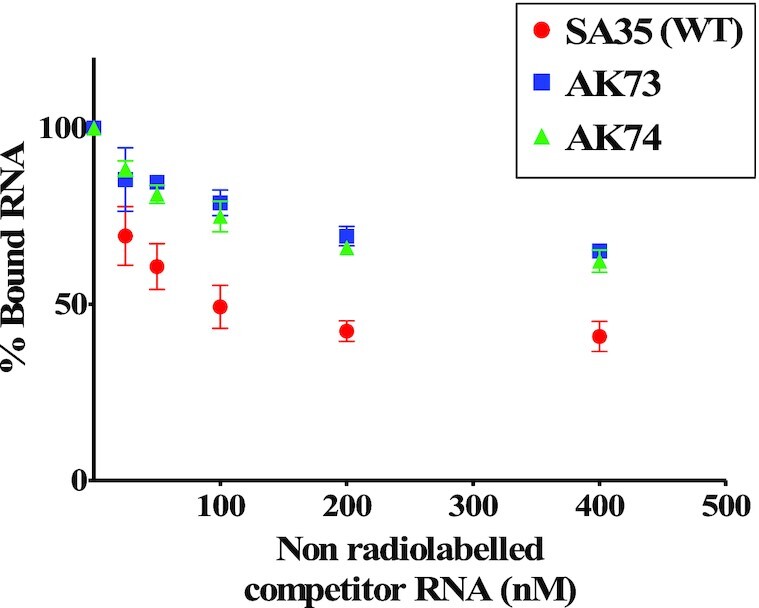
Band-shift competition assays for the clones containing mutations in nucleotides showing attenuation in SHAPE reactivities in PBS region (AK74) and basal part of SL3 (AK73; see Figure 10A for details of mutations introduced). Quantification of the gels showing the binding specificity of AK74 and AK73 RNAs to full-length MMTV Pr77^Gag^ analyzed by band-shift competition assay.

Our mutational analysis showed that in addition to ssPurines, mutations in the bottom part of SL3 and PBS region (AK73 and AK74) also showed a 40% and 96% reduction in their packaging abilities respectively when compared to WT (DA024). To determine if these nucleotides played a true role in Pr77^Gag^ binding, AK73 and AK74 mutations were tested in band-shift competition assays. Both mutations (AK73 and AK74) showed significantly reduced affinity to Pr77^Gag^ compared to the WT (SA35) in band-shift competition assays (Figure 11). In order to check whether this loss of affinity was due to loss of higher order structure, hSHAPE was performed on these mutants. In the case of mutation AK73, the first two SHAPE-validated structures showed different secondary structures when compared to the WT; however, structure 3 was very similar to WT (SA35), maintaining the conserved structural motifs (part of U5/*gag* LRI, SL2, SL3 and SL4) between nt 48–318 (Supplementary Figure S7). However, the AK74 mutation maintained an overall WT (SA35) structure with a prominent change at the PBS region, resulting in unpairing of the short stem-loop in the PBS structure of WT (SA35) RNA (Supplementary Figure S8). These data suggest that the 40% packaging defect observed in the mutation introduced in the bottom part of SL3 (AK73) is probably due to a loss of Gag binding to the RNA, attributed to a partial loss of structure of the packaging signal RNA. Alternatively, it is also possible that the mutated nucleotides in this region play some role in Gag binding. On the other hand, substitution mutations in the PBS region (AK74) did not perturb the RNA structure, suggesting that the PBS has a direct role in MMTV RNA packaging, by affecting Gag binding.

To further confirm the direct contribution of nucleotides in the PBS region towards Pr77^Gag^ binding, SHAPE footprinting experiments were performed using the mutation AK74 RNA (in which all the 7 nucleotides that showed attenuation of SHAPE reactivities in the presence of Gag were substituted; Figure 10A) employing the same conditions used for the footprinting of WT (SA35) RNA. Consistent with our expectations, the reactivities of these seven substituted nucleotides were not significantly reduced in the presence of Pr77^Gag^, indicating that Gag is not able to bind to these nucleotides (Supplementary Figure S9), which agrees with the reduced competition ability of the same mutation (AK74) compared to WT (Figure 11).

### The role of PBS in MMTV gRNA packaging is at the primary sequence level

**Figure 12.**
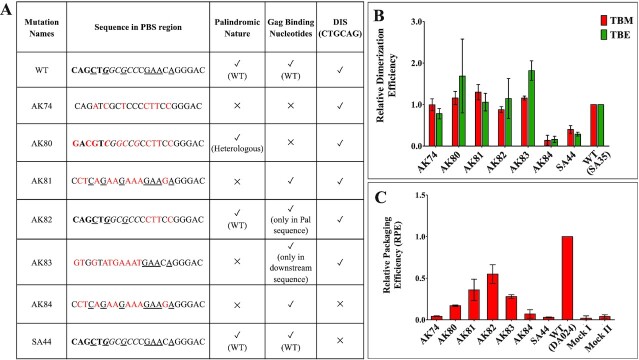
Role of PBS in dimerization and packaging of MMTV gRNA. (**A**) Table describing the nature of substitution mutations introduced into the PBS region. The bold nucleotides correspond to the first palindromic sequence within the overlapping pals while the second palindromic sequence is italicized. The underlined nucleotides represent the nucleotides which were protected from SHAPE modification by Pr77^Gag^ and substitutions are shown in red. (**B**) *In vitro* dimerization ability of PBS mutant RNAs related to that of WT (SA35) in native (TBM) and denaturing (TBE) conditions. (**C**) Packaging efficiencies of mutant transfer vector RNAs relative to the WT (DA024). Mock I contain only the packaging construct (without transfer vector) and Mock II has only transfer vector and no packaging construct. WT, wild type.

The PBS harbours overlapping palindromic sequences (PBS pal; 3′ **CAGCT*G****GCGCC* 5′; the bolded nucleotides represent first palindromic sequence, while the second palindromic sequence is italicized) that might affect RNA dimerization and, indirectly, RNA packaging (59). Thus, at this point, the loss of RNA packaging of the mutation AK74, in which the seven nucleotides that showed attenuation of SHAPE reactivities in the presence of Gag were substituted, that we reported in Figure 10 could be due to a direct effect on Pr77^Gag^ binding, or an indirect effect on RNA dimerization, since the two PBS pals lost their palindromic nature by introducing such a mutation. In order to clarify the precise role of PBS in MMTV gRNA packaging, a series of mutations were introduced in PBS that either disrupted its palindromic nature while maintaining the nucleotides that showed Pr77^Gag^ footprints or vice versa, with or without simultaneous mutations in the DIS (Figure 12A), followed by testing their RNA dimerization and encapsidation capabilities.

Briefly, starting with a DIS pal mutation (SA44), a double mutation was created in which the palindromic nature of DIS as well as that of PBS pal was lost (AK84; Figure 12A). In another mutation AK80, the seven nucleotides in the PBS region that showed attenuation of SHAPE reactivities in the presence of Gag were mutated while maintaining the palindromic nature of the PBS by substituting the 11-nucleotide native overlapping palindromic sequence (**CAGCT*G****GCGCC*) with a heterologous palindromic sequence (**GACGT*C****GGCCG*). On the other hand, mutation AK81 had disrupted the palindromic nature of the PBS while maintaining the nucleotides that showed attenuation of SHAPE reactivities in the presence of Gag. In the mutation AK82, the overlapping palindromes were maintained but the four downstream nucleotides that showed attenuation of SHAPE reactivities in the presence of Gag were substituted. Finally, in mutation AK83, the palindromic sequences were substituted with non-palindromic sequences concomitantly with substitution of three out of seven nucleotides at the 5′ end of the PBS showing Gag footprints, thus restricting the mutations to only the palindromic regions within PBS.

First, we compared the RNA dimerization ability of mutations in which the DIS pal (SA44), the PBS pal (AK74, AK81) or both palindromes (AK84) were mutated (Figure 12A). While RNA dimerization of SA44 and AK84 was severely compromised, AK74 and AK81 RNAs dimerized with wild type efficiency (Figure 12B & Supplementary Figure S10), indicating that the PBS pals are not required for efficient RNA dimerization. However, RNA packaging of all the three mutations (AK74, AK81 and AK84) was severely affected (Figure 12C and fractionation and cDNA controls in Supplementary Figure S11), suggesting a direct role of PBS sequences in RNA packaging, independent of gRNA dimerization.

Next, we compared the RNA packaging ability of the various mutations introduced in the 7 nucleotides of the PBS region which showed Gag footprints (Figure 8). In the case of AK80 (in which the palindromic nature of PBS was maintained), the RNA packaging efficiency was reduced by 83% (Figure 12C and fractionation and cDNA controls in Supplementary Figure S11). On the other hand, the mutation AK81 (in which the palindromic nature of the PBS was lost while maintaining the nucleotides that showed Gag footprints) revealed ∼60% reduction in packaging (Figure 12C), less drastic than AK80. Test of mutations AK82 (in which the 5′ part of the PBS containing the PBS pals was maintained but the downstream nucleotides showing Gag footprint were substituted), and AK83 (in which the substitutions were restricted only to the PBS pals) revealed a 50% and 86% reduction, respectively, in RNA packaging (Figure 12C). The dimerization ability was not affected in any of these mutations (Figure 12B).

Together, these results indicate that (i) the palindromic sequences in PBS are not required for RNA dimerization (see AK84 and AK81; Figure 12B), (ii) the nucleotides in PBS showing Gag footprints are important for RNA packaging (see AK74; Figure 12C), as well as other nucleotides in the PBS that do not show Gag footprints (see AK81; Figure 12C) and (iii) the 5′ region of PBS apparently plays a greater role in RNA packaging than the 3′ region (see AK82 versus AK83 in Figure 12C).

## DISCUSSION

The mechanism by which the Gag precursor selects and packages the retroviral genome, a key step in retroviral life cycle, remains largely unclear despite having been studied extensively. Most reported studies on gRNA packaging in the literature have been performed on HIV-1, where purine-rich apical or internal loops in hairpins structures have been proposed to govern gRNA packaging by functioning as Gag binding sites. In this study, to identify general rules underlying retroviral gRNA packaging, we addressed this aspect in MMTV, a *Betaretrovirus* that assembles in the cytosol, before migrating to the plasma membrane and budding. To that aim, we expressed and purified the full-length MMTV Pr77^Gag^ and compared its binding to WT and mutant gRNA fragments as well as to spliced *env* and *sag* viral mRNAs. We also performed footprinting with SHAPE reagents to identify Pr77^Gag^ binding sites on the WT gRNA. Our results reveal the presence of two specific Gag binding sites of non-redundant nature within the packaging signal RNA consisting of a purine loop and the primer binding site. Despite these sequences being present on both unspliced and spliced RNAs, Gag specifically bound only to unspliced RNA, since it is the only one that could adopt the native bifurcated stem-loop structure containing the looped purines. Thus, results presented in this study have important implications for how MMTV, in particular, and retroviruses/retrotransposons, in general, recognize gRNA for specific incorporation into the assembling virions. Our study reinforces the hypothesis proposed earlier that specific structural elements in the context of the larger RNA packaging signal act as high affinity binding site(s) for Gag protein recognition (17–19). Furthermore, these observations have important ramifications for the development of antiviral therapies that target the virion assembly process in retroviruses.

Previous studies have shown that the major packaging determinants for MMTV gRNA reside in the 5′ UTR and 120 nts of *gag* (56) and fold into a complex secondary structure with several SLs. Among these, the bifurcated SL4 contains the DIS in one of the apical loops and a stretch of ssPurines in the second apical loop; the main 5′ splice site that is used to generate the viral spliced RNAs, is located immediately after this ssPurine stretch (59). In this study, our *in vitro* binding and in cell packaging/transduction assays showed that ssPurines are indispensable for MMTV gRNA packaging and virus replication by directly binding Pr77^Gag^ (Figures 5, 6 and 9). Within the ssPurines, the GGAG at the 5′ end and the AG at the 3′ end, respectively, are crucial for packaging (Figure 9). Interestingly, a sequence similar to the MMTV 5′ GGAG was found to be involved in the Pr55^Gag^ binding in HIV-1 (in the form of an asymmetrical internal loop 5′ G/AGG 3′ (20) and HTLV-1 NC and MA binding (5′GAG 3′; 70). Similarly, in HIV-2, a 5′ GGRG 3′ motif located upstream of DIS was found to be important for RNA packaging and has been suggested as Gag binding site (71). Altogether, results presented here propose a possible mechanism towards selecting gRNA and suggest that the rather large packaging region on gRNA mapped earlier must maintain specific RNA structural motifs so that the ssPurines can be presented to Gag in a single-stranded manner for recognition during packaging.

Interestingly, our study reveals that the bifurcated SL4 of MMTV gRNA juxtaposes the DIS to the ssPurines that constitutes the primary Pr77^Gag^ binding site. This situation is reminiscent of HIV-1, where the apical and internal loops of a long hairpin (SL1) constitute the DIS (63,72) and primary Pr55^Gag^ binding site (20,43,48), respectively. This is all the more the case that in MMTV gRNA the two hairpins of the bifurcated SL4 may stack on top of each other (73). If this is the case, the MMTV DIS and Gag binding sites are collinear, as it has been reported in the case in HIV-1 (74). The spatial proximity of the DIS and primary Gag binding site may be a general phenomenon, and it would explain the observation that dimerization and packaging of retroviral gRNA are highly interconnected events (38,39,41,75). A slightly different situation may prevail in MLV, where dimerization of the gRNA seems to be required to expose a nearby sequence recognized by the nucleocapsid domain of Gag (76,77).

Packaging of gRNA into newly forming virus particles is highly selective, although cellular and spliced RNAs are also incorporated (14,16,56,78,79). Our binding assays with RNAs corresponding to the first 712 nts of unspliced gRNA and spliced *env* and *sag* mRNAs suggest that selective packaging of unspliced gRNA begins at the initial stages of viral assembly, which involves direct, specific binding of Pr77^Gag^ to unspliced gRNA and not to spliced RNAs. Indeed, despite the presence of the ssPurines in both spliced mRNAs (*env* and *sag*) used in our study, Pr77^Gag^ is unable to bind to those RNAs with high affinity (Figures 3 & 4). This is due to the fact that the major 5′ splice donor site is located immediately downstream of the ssPurines, and thus, the ssPurine hairpin structure is lost during splicing, an observation that was confirmed by hSHAPE analysis (Figure 7). This analysis revealed that the ssPurines in spliced RNAs are base-paired and unavailable for Gag binding, explaining the selective binding of Pr77^Gag^ to the unspliced gRNA over spliced RNAs. This is in line with previous observations that revealed that the bifurcated SL4 structure is crucial to maintain gRNA packaging (60). Of note, our previous study reported that the packaging determinants for MMTV gRNA reside in the entire 5′ UTR and extends up to 120 nt of *gag* (56). Interestingly, our footprinting experiments revealed no Pr77^Gag^ binding sites in the *gag* gene (Figure 8), but the region downstream of the mSD plays a critical function in maintaining the bifurcated structure of SL4. A similar observation has been made in the case of avian leukosis/sarcoma virus (ALSV) and suggest that the ALSV *env* mRNA acquires a packaging incompetent structure (80). Selective binding of the Gag precursor to gRNA but not to spliced viral RNAs has also been observed in the case of HIV-1, despite the fact that the major packaging sequence (5′G/AGG 3′), forming the internal loop of SL1, is present in both unspliced and spliced RNAs (20). In this case the structure of SL1 is identical in gRNA and spliced viral RNAs, but sequences upstream of SL1 prevent Gag binding if a short region downstream of SL3, which is part of gRNA but not spliced RNAs, is not present (20,43,48). Regions upstream of SL1 and downstream of SL3 maintain a 3D structure that exposes the lower part SL1 for Pr55^Gag^ binding (20,43,48). It has also been shown that in HIV-1, the long-range interactions (LRI) between U5 and the *gag* initiation codon stabilizes a dimerization-competent RNA structure, which in turn, may lead to packaging (81–84). Further studies will be required to elucidate the precise role of U5-*gag* LRIs (Figure 8), if any, in MMTV gRNA packaging.

Our footprinting assays revealed that Pr77^Gag^ induces attenuation of SHAPE reactivity not only in the ssPurines loop, but also in nucleotides of other regions (Figure 8 and Supplementary Figure S5). To determine whether reduced reactivity was due to direct Pr77^Gag^ binding or due to Pr77^Gag^-induced structural changes, we introduced region-wise mutations in these nucleotides and assayed their effects on in cell RNA packaging and transduction (Figure 10). Mutations in the apical part of SL2, the bulge downstream of SL4, and the unpaired region upstream of SL5 did not have any significant effect on the packaging efficiency. The mutation of 3 nucleotides in the basal part of SL3 (Figure 10), showed a 40% reduction in the packaging efficiency; however, the SHAPE-validated structures suggest that this RNA may exist as a mixture of dynamical structures and a portion of these RNAs may assume a wild type structure (Supplementary Figure S7). One possibility for reduced packaging, transduction, and *in vitro* Gag binding of this mutant RNA could be attributed to the existence of these alternate RNA conformations. Since these mutations resulted in only 40% reduction of packaging efficiency, we are not considering these nucleotides as a critical requirement for packaging.

Our *in vitro* and in cell assays showed that the PBS region binds to Gag and is a critical element for packaging (Figures 9 and 10). The PBS region contains two overlapping palindromic sequences, and our previous study has shown that deletion of these sequences results in a drastic reduction of dimerization ability of the MMTV gRNA (59). By conducting a detailed mutational analysis of the PBS region combined with dimerization and packaging assays of the mutant RNAs, we observed that the PBS does not play a direct role in the dimerization of MMTV gRNA since loss of its palindromic nature did not show any significant reduction in RNA dimerization (Figure 12B and Supplementary Figure S10). On the other hand, the PBS sequence is critical for RNA packaging. Of note, a palindrome in the HIV-2 PBS has been proposed to be involved in its dimerization (85), but to the best of our knowledge, a role of this PBS palindrome in HIV-2 gRNA packaging has never been demonstrated. In the case of HIV-1, the core Pr55^Gag^ binding domain encompasses the extreme 3′ end of the PBS region (43) and secondary Gag binding sites have been identified in this domain by footprinting (86,87). Studies using annealing of either tRNA^Lys3^ or oligonucleotides complimentary to PBS showed an increase in HIV-1 RNA dimerization by enhancing the dimerization-competent RNA conformation, whereas deletion of PBS resulted in a moderate reduction in packaging (81,88).

The precise role of PBS in HIV-1 gRNA packaging is rather elusive, essentially relying on drastic mutations that potentially could have compromised the global RNA secondary structure (89). Conversely, systematic point substitutions in the 5′ region of the HIV-1 gRNA did not identify sequences in the PBS contributing to RNA packaging (42). In the case of retrotransposon Ty1, the bipartite PBS located at both the 5′ and 3′ UTRs was observed to be necessary for packaging, possibly by acting as a dimerization site mediated by the hybridization of tRNA_i_^Met^ (90). Subsequently, it was shown that the Gag-induced dimerization was not required for annealing of tRNA to PBS (37), and similarly in Ty3, the annealing of tRNA was also not required for RNA dimerization, suggesting that the bipartite PBS is not critical for gRNA packaging of either Ty1 or Ty3 retrotransposons (45,91). In light of these observations, the role of the PBS domain in Gag binding and RNA packaging presented in this study might be specific for MMTV or *Betaretroviruses*.

It has been suggested that in HIV-1, Pr55^Gag^ is involved in the initial placement of tRNA to the PBS, while NCp7 in mature virions facilitates the formation of more stable tRNA-gRNA complexes (92,93). It is also becoming clear that the selective packaging of tRNA in the assembling virions is facilitated by increasing the local concentration of lysyl-tRNA synthetase and is independent of gRNA (83,85). During this process, Gag plays a central role in recruiting the tRNA^Lys3^- lysyl-tRNA synthetase complex through the specific binding of its CA domain to lysyl-tRNA synthetase (reviewed in (93,94). Thus, in case of MMTV, we speculate that the Pr77^Gag^ binding to the PBS region may play dual role in MMTV replication by ensuring first selection of the gRNA during the early stages of the viral assembly process, then initial annealing/placement of tRNA^Lys3^ to the PBS once the immature particles are formed.

Taken together, our *in vitro* binding and in cell RNA packaging/transduction assays identify two regions, the architecturally accessible ssPurines loop of SL4 and the PBS domain, critical for the packaging of MMTV gRNA by Pr77^Gag^. The spatial proximity of ssPurines to the DIS, which is reminiscent to the situation in HIV-1, may provide a molecular explanation for the strong link between gRNA dimerization and packaging that has been described for many retroviruses and retrotransposons. Consistent with this, packaging determinants have mostly been shown to overlap with, or found to be in close proximity to, sequences responsible for augmenting dimerization while having a higher order structure. On the contrary, the role of MMTV PBS domain in gRNA packaging demonstrated here is most likely not a general phenomenon among retroviruses. Finally, the discrimination between unspliced and spliced RNAs begins at the initial stages of assembly and could primarily rely on maintaining the structural integrity of the bifurcated SL4. These results provide insights into the molecular mechanisms involved in the packaging of a much less studied *Betaretrovirus*, MMTV, and allow distinguishing feature that are conserved amongst divergent retroviruses, such as MMTV and HIV-1, from those that are virus-specific.

In conclusion, this study demonstrates that MMTV Gag recognizes ssPurines fundamentally on the basis of the higher order structure during gRNA packaging in an infected cell, suggesting that certain structural motifs in secondary or tertiary RNA conformation(s) mediate RNA–protein interaction. Since gRNA packaging during virus assembly is vital for the continuity of viral life cycle, these findings have ramifications towards the development of therapeutic interventions based on unique antiretroviral drugs that target virus assembly, especially given the fact that only retroviral Gag is required for virus particle formation. These drugs could then target specific structural elements (such as ssPurines and PBS) within the packaging signal RNA or its interaction domains within the Gag protein using novel small molecule approaches (19,95,96). Unfortunately, a lack of basic understanding of these fundamental processes has prevented the development of such therapeutic modalities that can target retrovirus assembly. Thus, information gleaned from our study significantly adds to the developing literature of how retroviruses recognize their gRNA to initiate the process of virion particle assembly which can facilitate the development of such novel antivirals into the realm of reality.

## DATA AVAILABILITY

All data pertaining to this manuscript has been provided as supplementary data.

## Supplementary Material

gkab223_Supplemental_Files
