# Supplementary material for: A purine loop and the primer binding site are critical for the selective encapsidation of mouse mammary tumor virus genomic RNA by Pr77Gag
Source: Nucleic Acids Res. 2021 Apr 9;49(8):4668–88. doi: 10.1093/nar/gkab223 (PMC8096270; doi:10.1093/nar/gkab223)
Supplement: gkab223_Supplemental_Files [file gkab223_supplemental_files.zip › Supplementary Figures Legends.docx]

# Supplementary Figures Legends

**Supplementary Figure 1**. Design and rationale of the MMTV three-plasmid genetic complementation assay. Virus particles produced by the MMTV Gag/Pro/Pol expression plasmid (JA10) and pseudotyped by vesicular stomatitis virus envelope glycoprotein (VSV-G) expressed by MD.G allow packaging of MMTV subgenomic transfer vector (DA024) RNA due to the presence of the packaging signal (Ψ). 293T cells that have been co-transfected with the 3 plasmids produce infectious virus particles and RNAs from these cells are fractionated into nuclear and cytoplasmic fractions. The cytoplasmic fractions are analyzed for proper RNA expression and the virus particles harvested from the transfected 293T cells are tested for the amount of RNA packaged using RT-qPCR. Viral supernatants are also used to infect target HeLa cells to study propagation of the packaged RNA in a single round of replication assay. After infection, target cells are selected with media containing hygromycin B antibiotic, allowing only those cells to survive which have been transduced by the RNA since the packaged RNA contains the *hygromycin resistance* gene cassette.

**Supplementary Figure 2.** Dynamic light scattering of purified Pr77^Gag^ in the RNA binding buffer. **A)** Protein number *versus* hydrodynamic radius (Rh) distribution, and **B)** Protein mass *versus* hydrodynamic radius (Rh) distribution.

**Supplementary Figure 3**. *In vitro* dimerization ability of the WT (SA35) and the ssPurine mutant RNAs (AK18, AK62 & AK63). These mutant RNAs show minimal effects, suggesting that the loss of Pr77^Gag^ binding is not due to the impairment in *in vitro* RNA dimerization. WT, wild type.

**Supplementary Figure 4**. Band-shift assay showing the formation of a single high-affinity Pr77^Gag^- radiolabeled gRNA complex under the exact conditions used for footprinting experiments.

**Supplementary Figure 5**. Histograms showing the Pr77^Gag^-induced attenuation of the SHAPE reactivities of **A)** ssPurines, **B)** apical part of SL2, **C)** PBS region, **D)** basal part of SL3, and **E)** bottom region downstream of SL4 and unpaired region upstream of SL5 (*p* value ≤ 0.05; tested by paired, two-tailed Student’s *t*-test).

**Supplementary Figure 6.** Control PCR amplifications used to test DNase-treatment of RNA, cDNA synthesis, and integrity of nucleocytoplasmic cell fractionation of RNAs with mutations that have been introduced in regions other than ssPurines. PCR amplifications of the DNase-treated cytoplasmic (panel I) and viral (panel II) RNAs using MMTV-specific primers (169 bp). Panels III and IV show PCR amplifications of the cytoplasmic and viral cDNAs, respectively, using MMTV-specific primers (169 bp). Multiplex amplifications were conducted in the presence of primers/competimer for 18S rRNA (324 bp) and unspliced β-actin mRNA (200 bp). Lack of any amplifications of unspliced β-actin mRNA in panel V confirms that there was no contamination of cytoplasmic RNA fractions with that of nuclear fraction, while amplification of 18S rRNA confirms the quality of the cDNA. Panel VI shows amplification of cytoplasmic cDNA using primers that amplify spliced β-actin mRNA (249 bp). Mock I contain only the packaging construct (without transfer vector) and Mock II has only transfer vector and no packaging construct.

**Supplementary Figure 7**. SHAPE-validated secondary structure of wild type (SA35) and mutant containing substitutions in the bottom part of SL3 (AK73; A237, G238 and A239 to U237, C238 and U239) RNAs, **A)** Wild type (SA35) **B)** AK73 structure 1 obtained from the software RNAstructure 6.1, Energy (E) = -274.8 kcal/mol. **C)** Structure 2, E = -279.6 kcal/mol. **D)** Structure 3, E = -270.6 kcal/mol. SHAPE data were used as constraints for all structures.

**Supplementary Figure 8.** SHAPE-validated RNA secondary structure of **A)** Wild type (SA35) and **B)** AK74 containing substitution mutations in the primer binding site (PBS) region; structure number 2 from the RNAstructure 6.1, Energy (E) = -280.8 kcal/mol. SHAPE data were used as constraints for all structures.

**Supplementary Figure 9**. Histograms showing SHAPE reactivities of the mutation (AK74) introduced into the PBS region in the absence and presence of Pr77^Gag^.

**Supplementary Figure 10.** Representative gel picture showing *in vitro* dimerization of WT gRNA (SA35) and PBS mutant RNAs (SA44, AK74, AK80, AK81, AK82, AK83 and AK84) in native (TBM) and denaturing (TBE) conditions. M & D at the bottom of the gel figure indicates the monomer and dimer buffers in which the dimerization assays were performed. Following dimerization, electrophoresis was performed using both the native (TBM) and denaturing (TBE) gels with respective running buffers. The M and D in the left side of the figure indicates the position of the monomeric and dimeric RNA species, respectively. WT, wild type.

**Supplementary Figure 11.** Control PCR amplifications used to test DNase-treatment of RNA, cDNA synthesis, and integrity of nucleocytoplasmic cell fractionation of RNAs with mutations that have been introduced in the PBS region. PCR amplifications of the DNase-treated cytoplasmic (panel I) and viral (panel II) RNAs using MMTV-specific primers (169 bp). Panels III and IV show PCR amplifications of the cytoplasmic and viral cDNAs, respectively, using MMTV-specific primers (169 bp). Multiplex amplifications were conducted in the presence of primers/competimer for 18S rRNA (324 bp) and unspliced β-actin mRNA (200 bp). Lack of any amplifications of unspliced β-actin mRNA in panel V confirms that there was no contamination of cytoplasmic RNA fractions with that of nuclear fraction, while amplification of 18S ribosomal RNA confirms the quality of the cDNA. Panel VI shows amplification of cytoplasmic cDNA using primers that amplify spliced β-actin mRNA (249 bp). Mock I contain only the packaging construct (without transfer vector) and Mock II has only transfer vector and no packaging construct.
