# Supplementary material for: A purine loop and the primer binding site are critical for the selective encapsidation of mouse mammary tumor virus genomic RNA by Pr77Gag
Source: Nucleic Acids Res. 2021 Apr 9;49(8):4668–88. doi: 10.1093/nar/gkab223 (PMC8096270; doi:10.1093/nar/gkab223)

Supplementary Figure 1

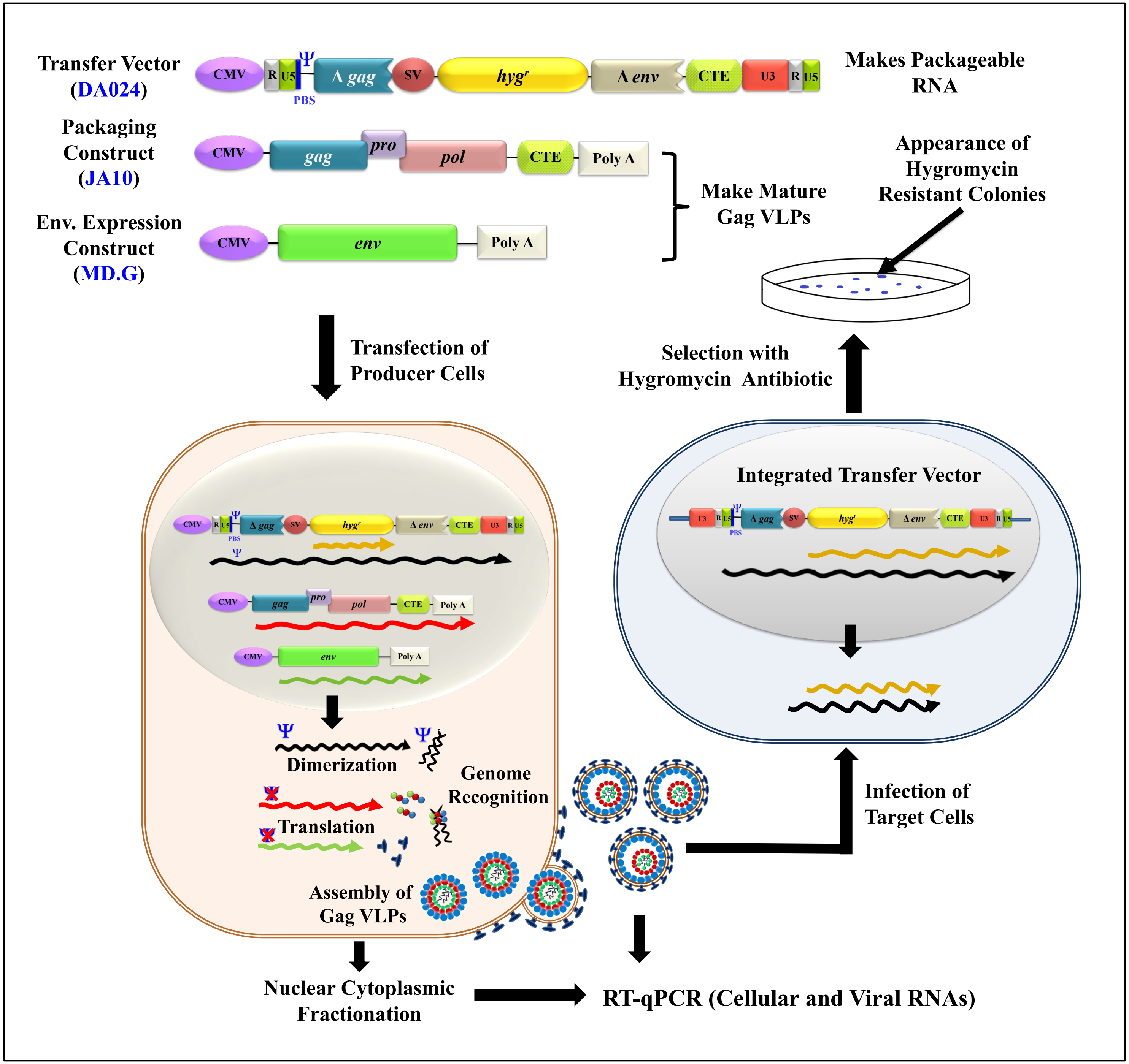

Supplementary Figure 2

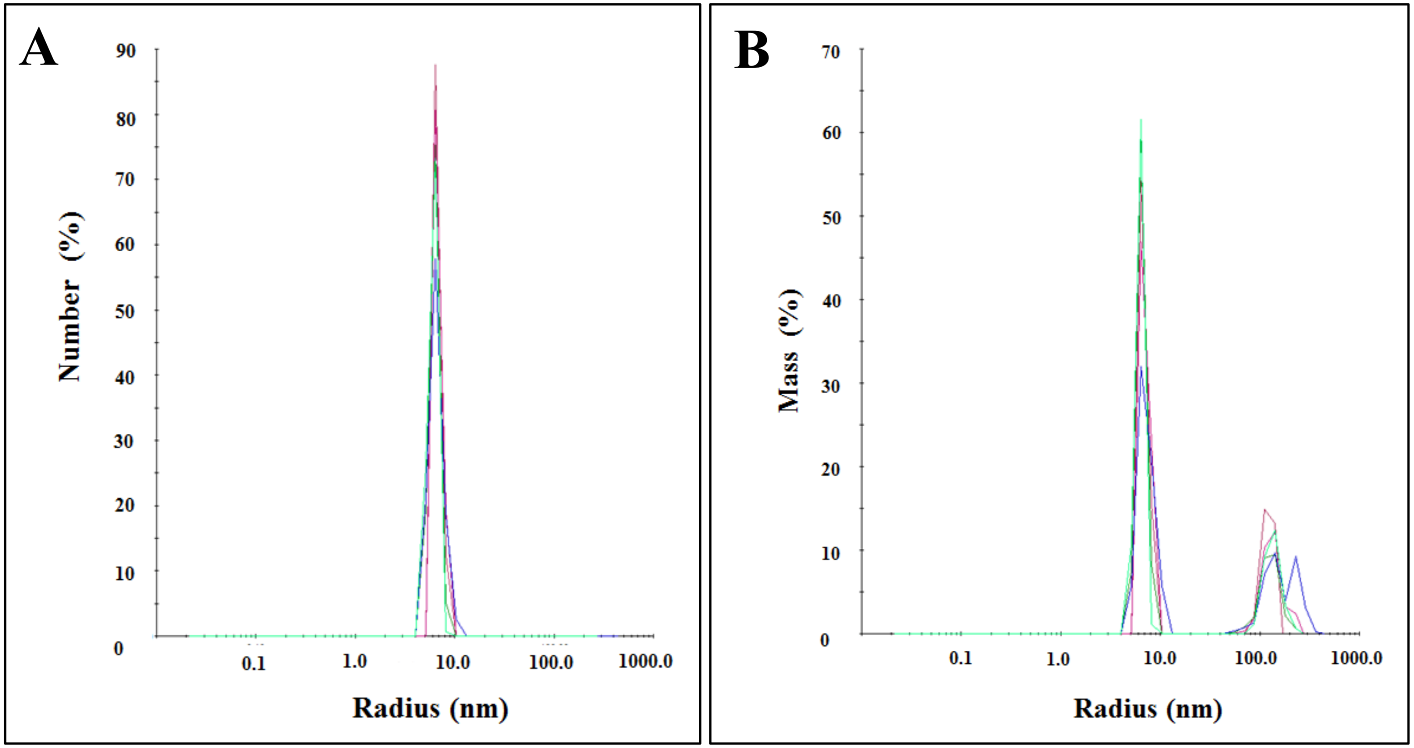

Supplementary Figure 3

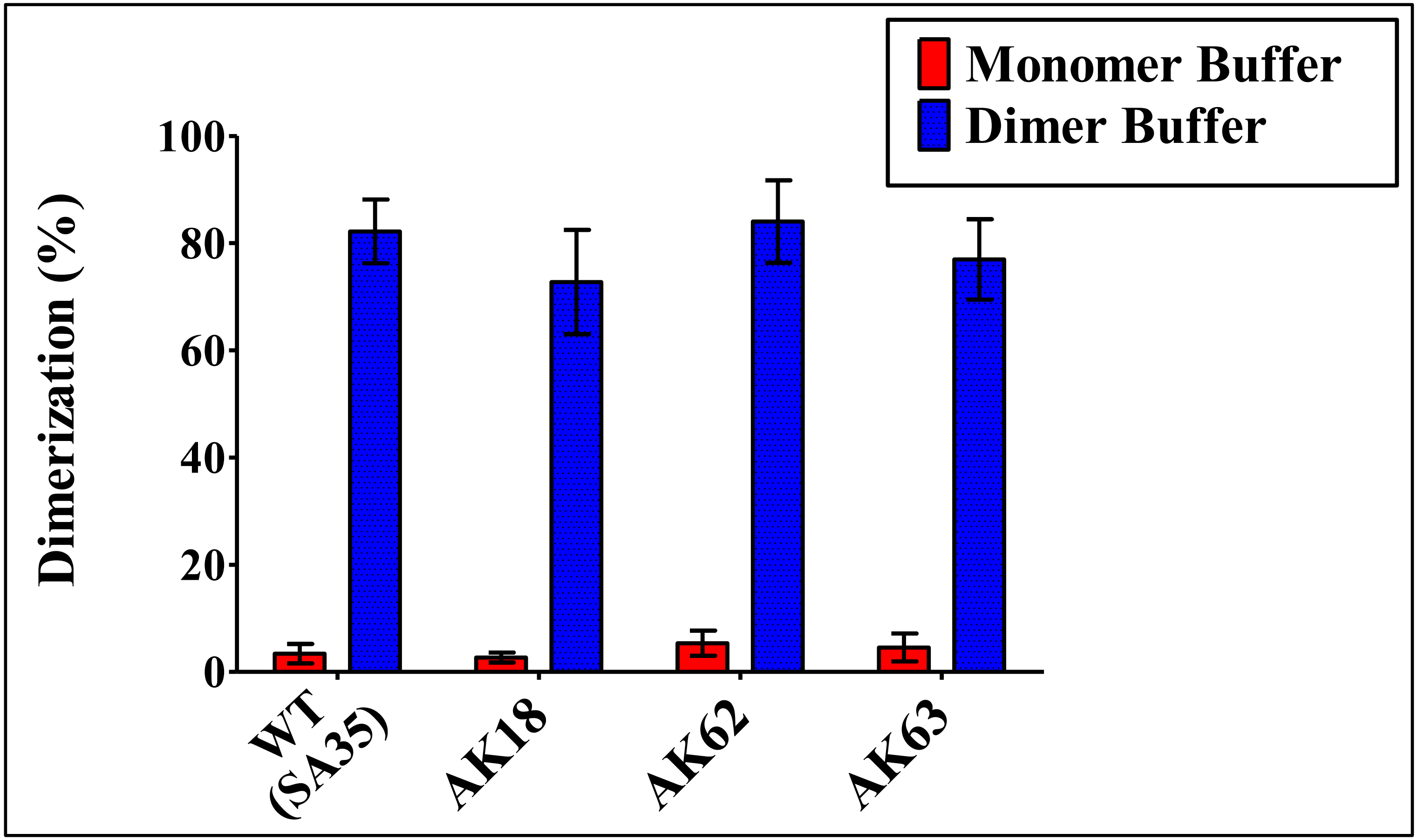

Supplementary Figure 4

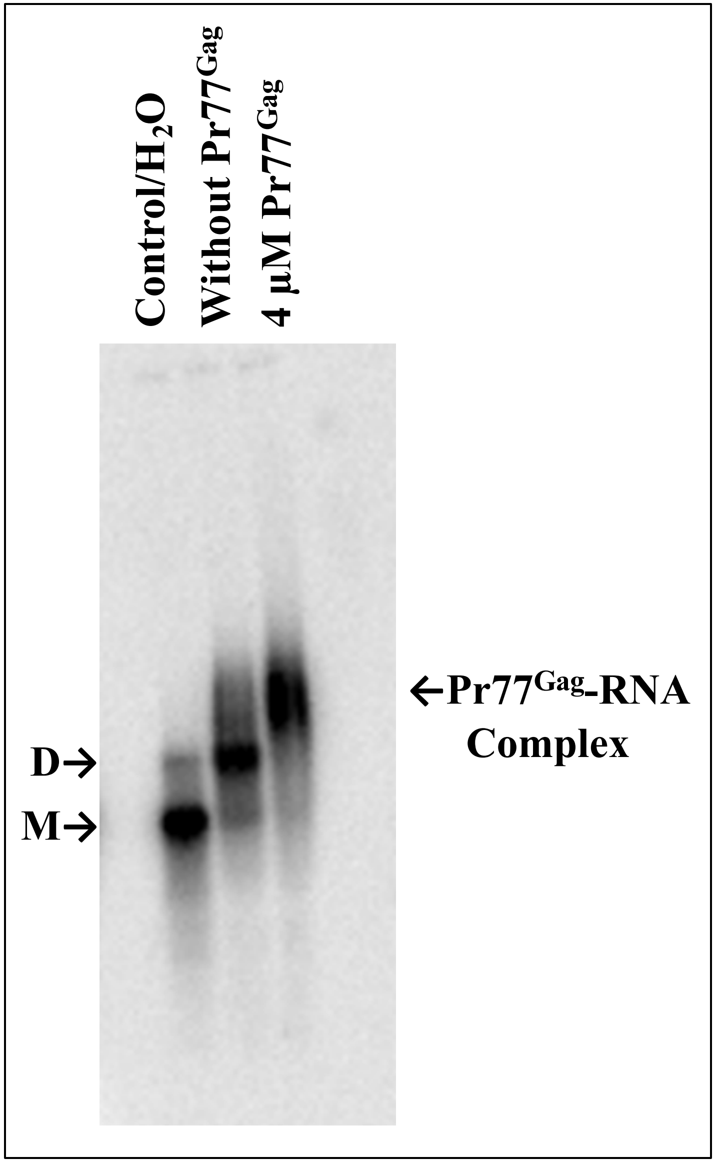

Supplementary Figure 5

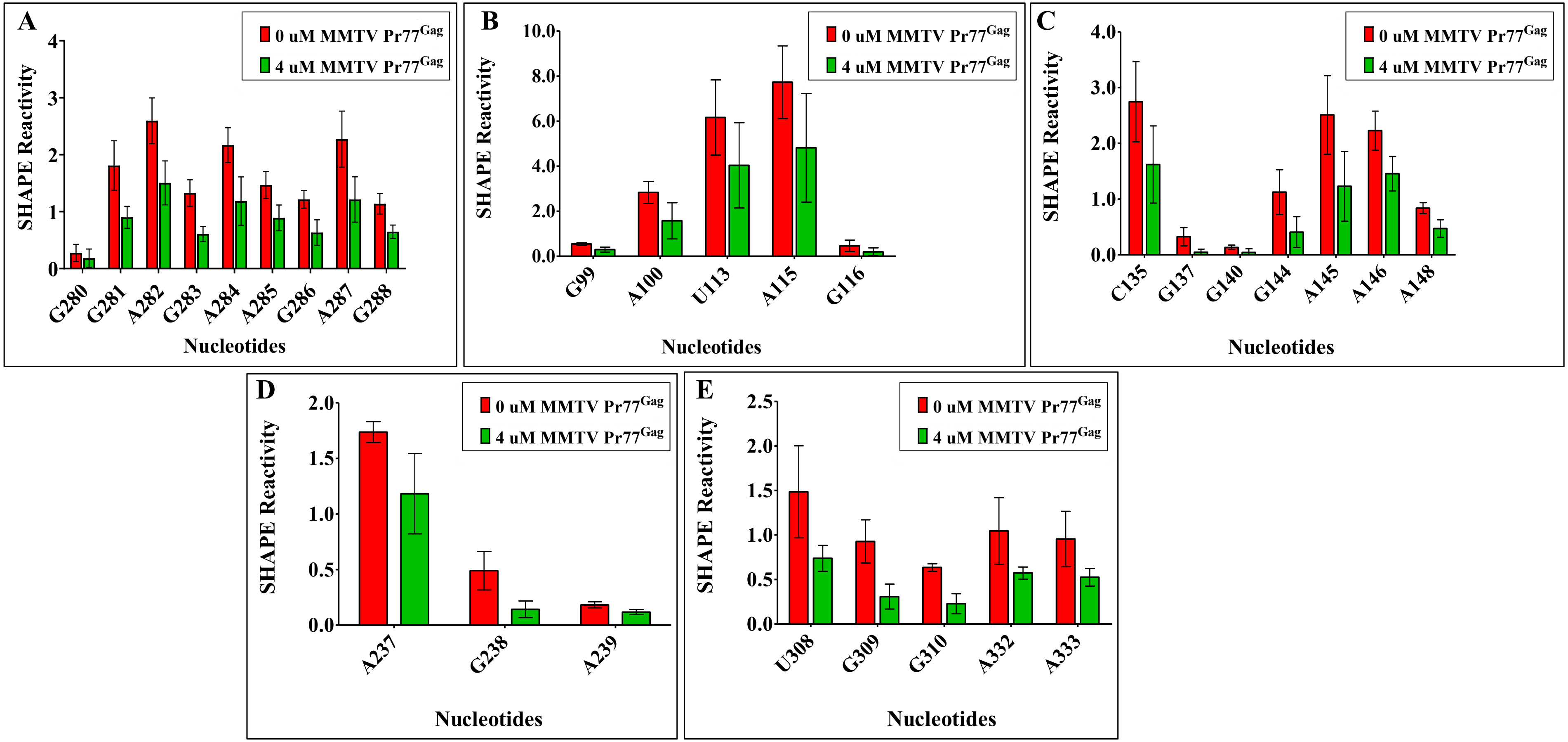

Supplementary Figure 6

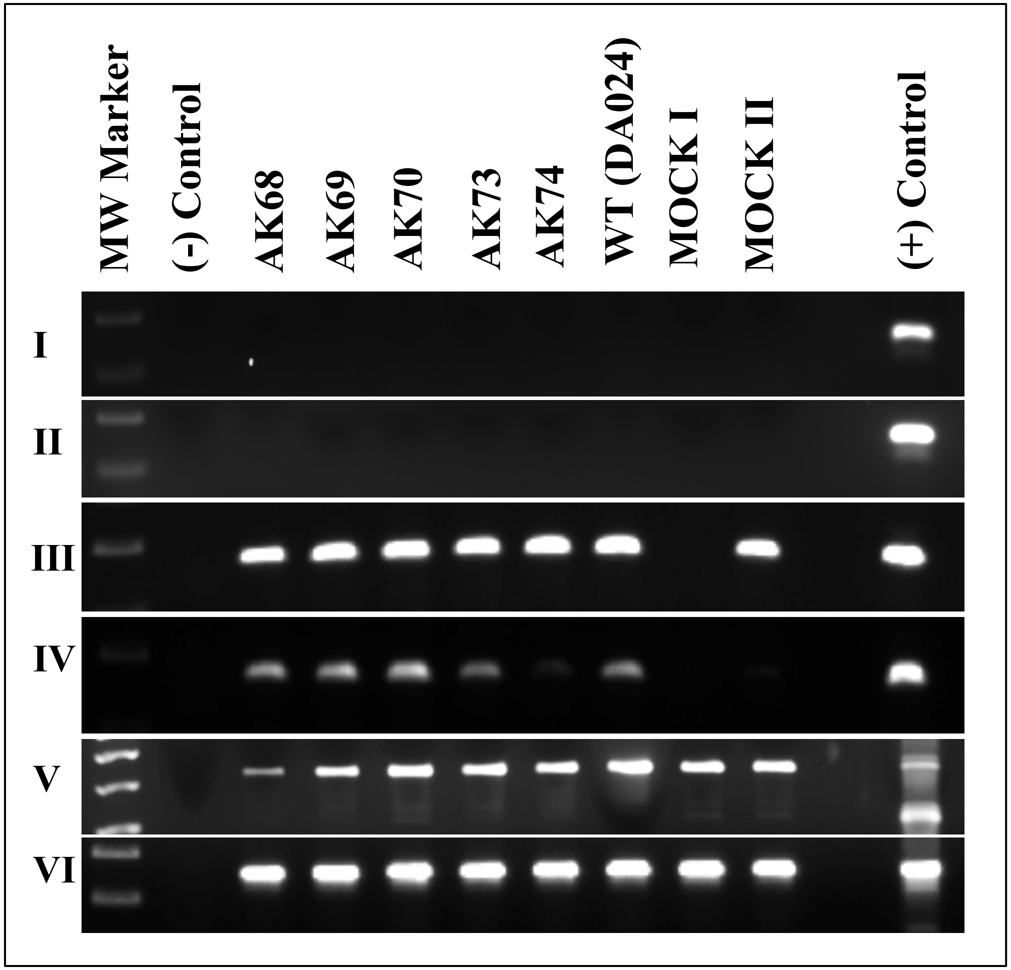

Supplementary Figure 7

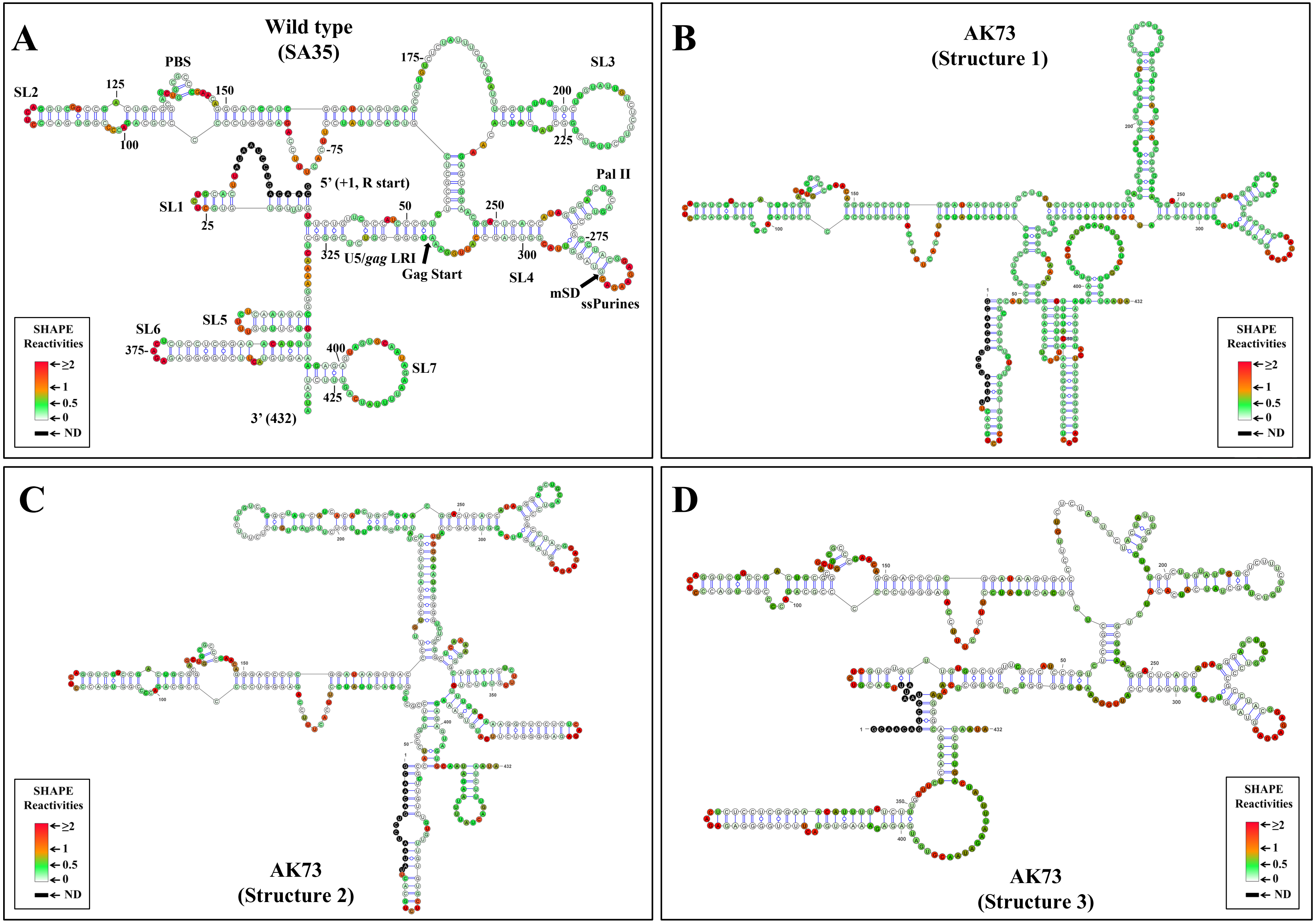

Supplementary Figure 8

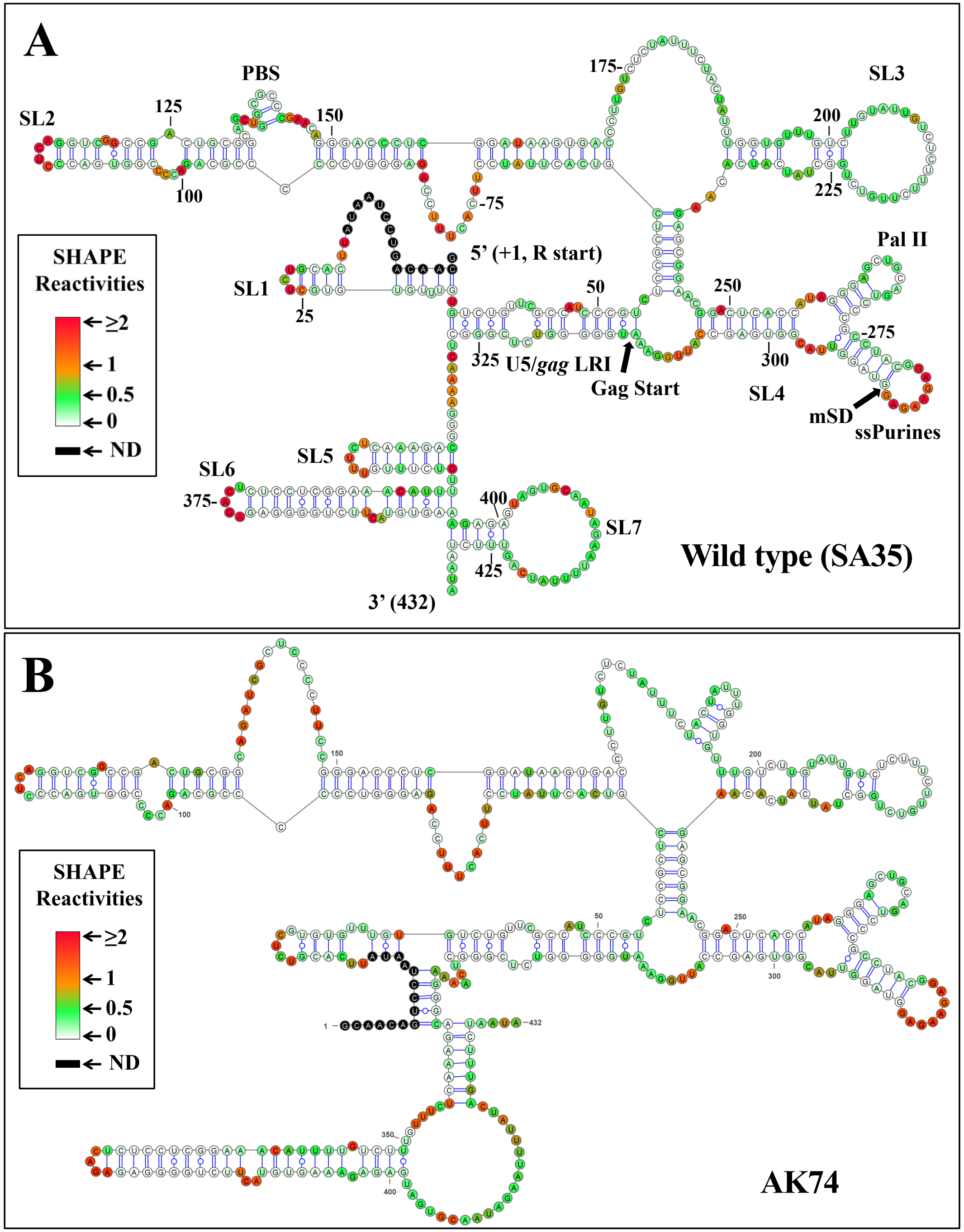

Supplementary Figure 9

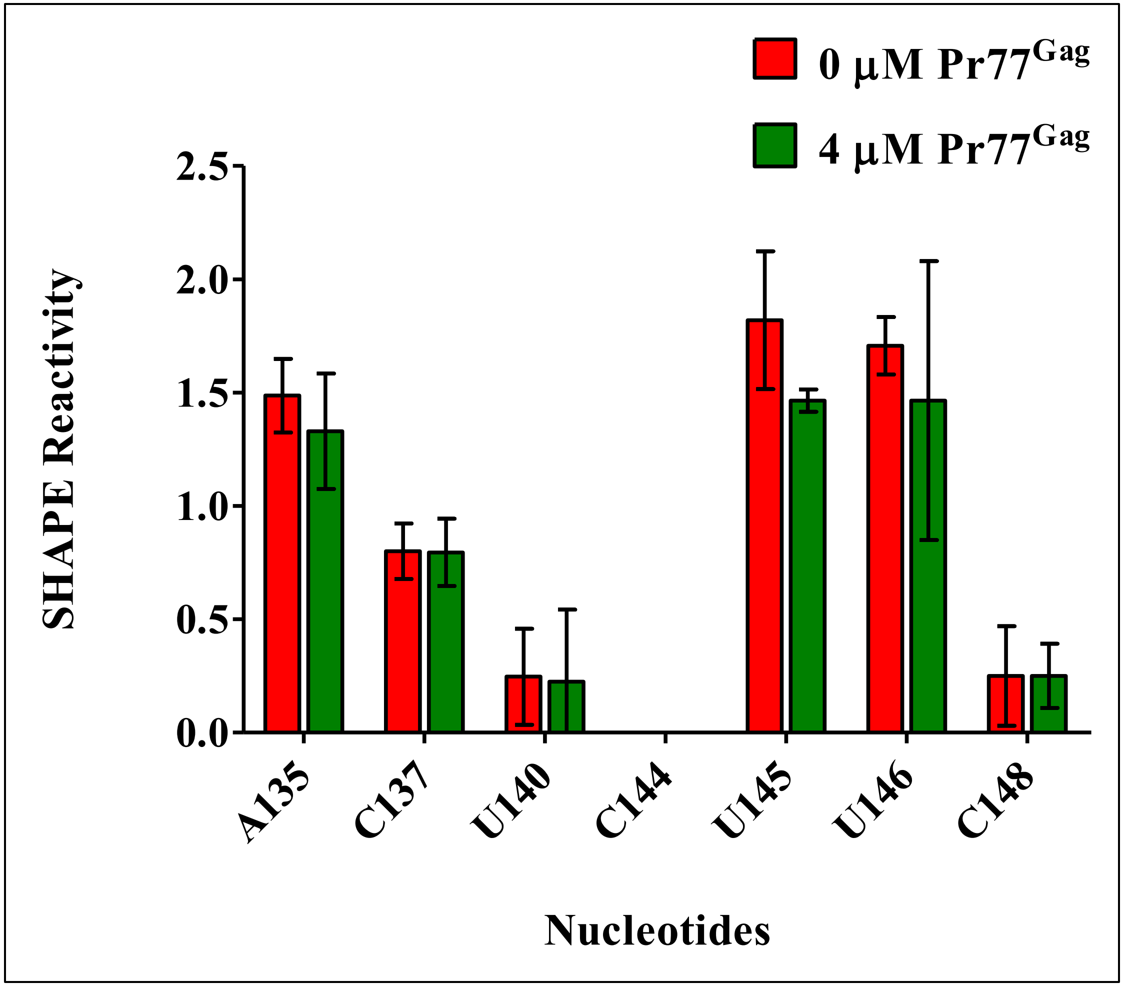

Supplementary Figure 10

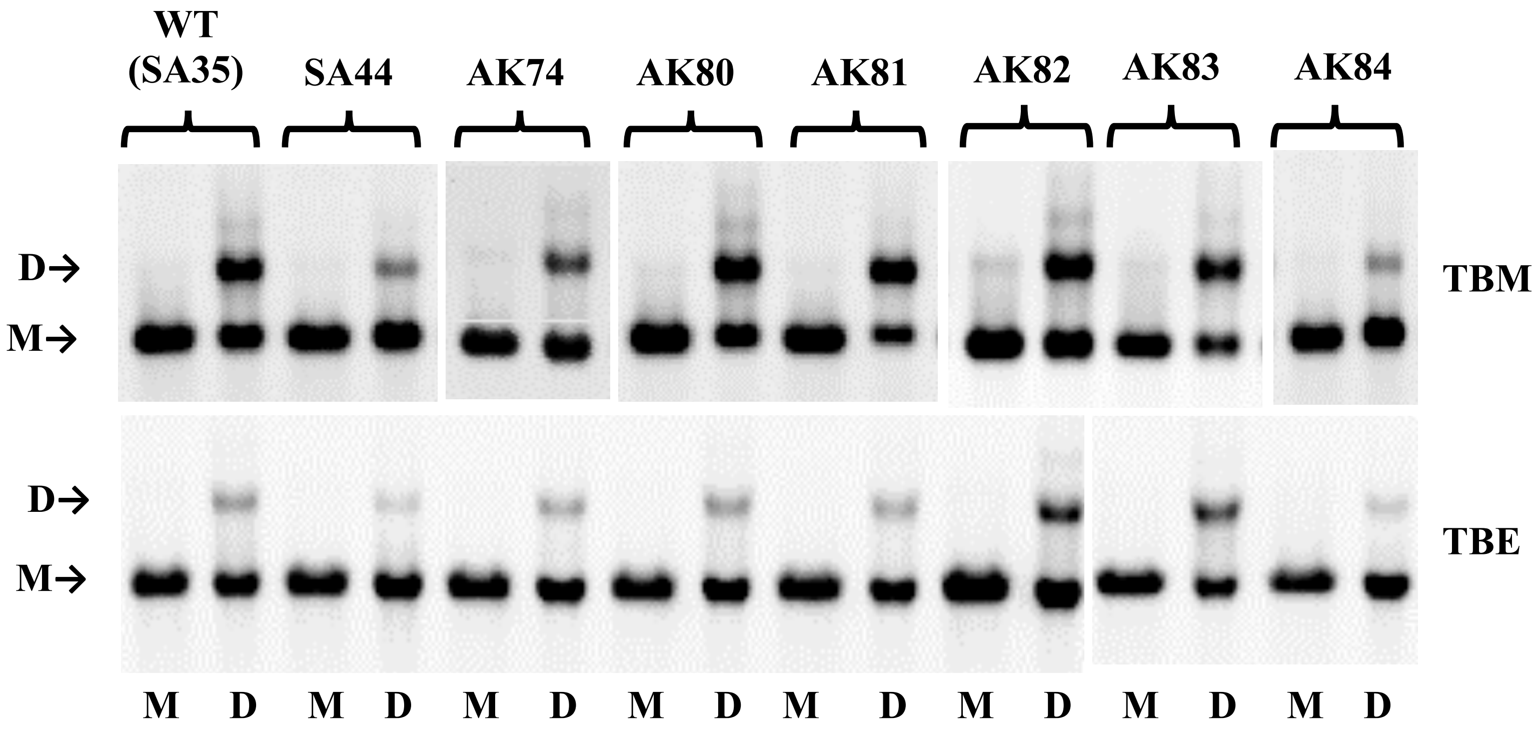

Supplementary Figure 11

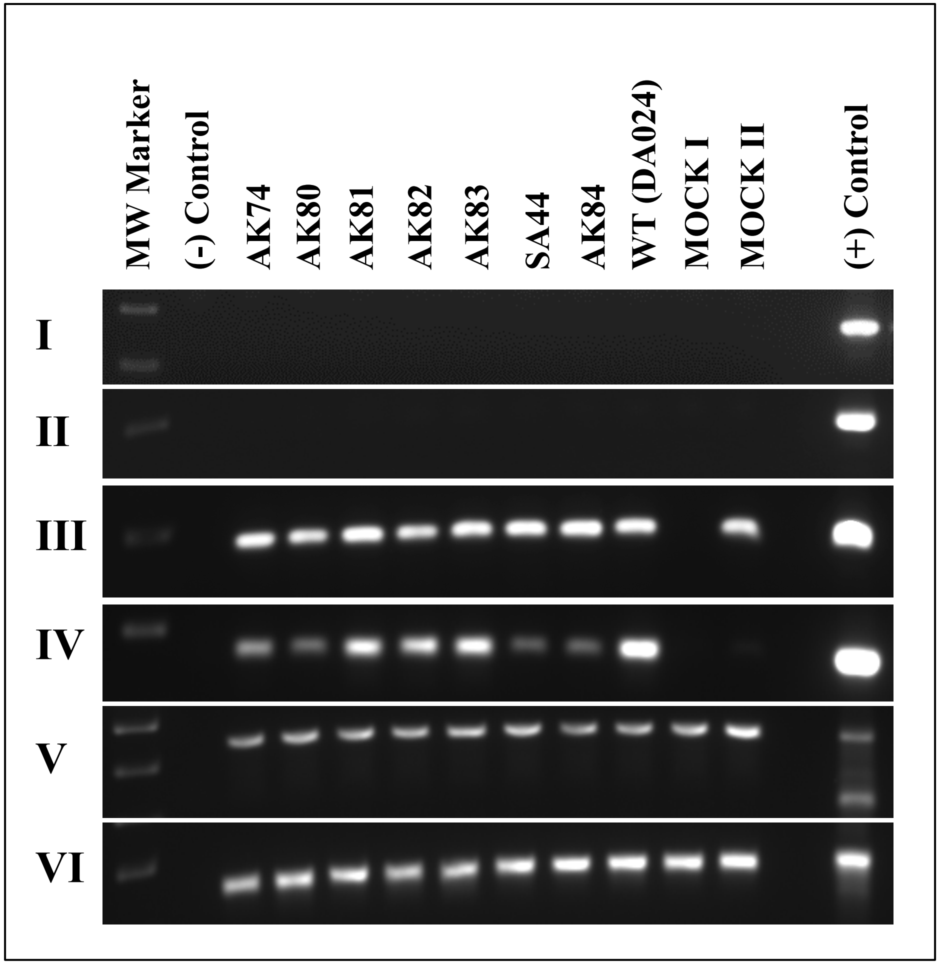

Supplement: gkab223_Supplemental_Files [file gkab223_supplemental_files.zip › Supplementary Figures.pdf]
