# Supplementary material for: A purine loop and the primer binding site are critical for the selective encapsidation of mouse mammary tumor virus genomic RNA by Pr77Gag
Source: Nucleic Acids Res. 2021 Apr 9;49(8):4668–88. doi: 10.1093/nar/gkab223 (PMC8096270; doi:10.1093/nar/gkab223)
Supplement: gkab223_Supplemental_Files [file gkab223_supplemental_files.zip › Supplementary Table 1.pdf]

Supplementary Table 1 (pages 1-6)

Primers used for introduction of mutations and construction of clones						
**Mutation Names**	**Primers**	**Region where mutations were introduced/ description**	***S or AS**	**Sequence**	**Nucleotide position**	**Starting plasmid**
AK68	OTR 1448	Apical part of SL2	AS	5’ TGC CGC ACT CGG CCG ACA GGT GTG TCA CCG GGG GGT GCG GGG GGA CCC TCT GGA A 3’	HYB-MTV 1251-1305	DA024 (Aktar *et al.*,2014)
	OTR 1449		S	5’ GTC GGC CGA GTG CGG CA 3’	HYB-MTV 1289-1305	
AK69	OTR 1450	Bulge downstream of SL4	AS	5’ AGC CCG AGA CCC CCA TTT GGT ATG GCT CAC CGT AAC CTA CCT C 3’	HYB-MTV 1458-1500	DA024
	OTR 1451		S	5’ AAA TGG GGG TCT CGG GCT 3’	HYB-MTV 1483-1500	
AK70	OTR 1452	Unpaired region upstream of SL5	AS	5’ AGA AAC AAA GAG TTT CTG CCC CCT TGA GCC CGA GAC CCC CAT TT 3’	HYB-MTV 1483-1526	DA024
	OTR 1453		S	5’ GGG CAG AAA CTC TTT GTT TCT 3’	HYB-MTV 1506-1526	
AK73	OTR 1454	Basal part of SL3	AS	5’ TAT GGT GAG TCC GTT CCG CAG ATG TGA TGA TAG CCA GAC AAG AAA G 3’	HYB-MTV 1385-1430	DA024
	OTR 1455		S	5’ GCG GAA CGG ACT CAC CAT A 3’	HYB-MTV 1412-1430	
AK74	OTR 1456	PBS region	AS	5’ TCA CTT ATC CGA GGG TCC CGG AAG GGG AGC GAT CTG CCG CAG TCG GCC GAC C 3’	HYB-MTV 1288-1339	DA024
	OTR 1457		S	5’ GGG ACC CTC GGA TAA GTG A 3’	HYB-MTV 1321-1339	
AK80	OTR 1580	PBS region	AS	5’ TCA CTT ATC CGA GGG TCC CGG AAG GCG GCC GAC GTC CCG CAG TCG GCC GAC C 3’	HYB-MTV 1288-1339	DA024
	OTR 1457		S	5’ GGG ACC CTC GGA TAA GTG A 3’	HYB-MTV 1321-1339	
AK81	OTR 1581	PBS region	AS	5’ TCA CTT ATC CGA GGG TCC CTC TTC TTT CTT CTG AGG CCG CAG TCG GCC GAC C 3’	HYB-MTV 1288-1339	DA024
	OTR 1457		S	5’ GGG ACC CTC GGA TAA GTG A 3’	HYB-MTV 1321-1339	

Supplementary Table 1 (continued)

Primers used for introduction of mutations and construction of clones						
**Mutation Names**	**Primers**	**Region where mutations were introduced/ description**	***S or AS**	**Sequence**	**Nucleotide position**	**Starting plasmid**
AK82	OTR 1582	PBS region	AS	5' TCA CTT ATC CGA GGG TCC CGG AAG GGG CGC CAG CTG CCG CAG TCG GCC GAC C 3'	HYB-MTV 1288-1339	DA024
	OTR 1457		S	5' GGG ACC CTC GGA TAA GTG A 3'	HYB-MTV 1321-1339	
AK83	OTR 1583	PBS region	AS	5' TCA CTT ATC CGA GGG TCC CTG TTC ATT TCA TAC CAC CCG CAG TCG GCC GAC C 3'	HYB-MTV 1288-1339	DA024
	OTR 1457		S	5' GGG ACC CTC GGA TAA GTG A 3'	HYB-MTV 1321-1339	
AK84	OTR 1581	PBS region	AS	5' TCA CTT ATC CGA GGG TCC CTC TTC TTT CTT CTG AGG CCG CAG TCG GCC GAC C 3'	HYB-MTV 1288-1339	SA044P (Aktar *et al.*,2014)
	OTR 1457		S	5' GGG ACC CTC GGA TAA GTG A 3'	HYB-MTV 1321-1339	
AK67	OTR 1460	ssPurines	AS	5' AAT GGC TCA CCG TAA CCT ACC TCT TCT CGG TAG GCG GGA CTG CAG CTC 3'	HYB-MTV 1433-1480	DA024
	OTR 1405		S	5' GTA GGT TAC GGT GAG CCA TT 3'	HYB-MTV 1461-1480	
AK44	OTR 1396	ssPurines	AS	5' AAT GGC TCA CCG TAA CCT ACC TGA AGT CCG TAG GCG GGA CTG CAG CTC 3'	HYB-MTV 1433-1480	DA024
	OTR 1405		S	5' GTA GGT TAC GGT GAG CCA TT 3'	HYB-MTV 1461-1480	
AK45	OTR 1397	ssPurines	AS	5' AAT GGC TCA CCG TAA CCT ACG ACT TCA GGG TAG GCG GGA CTG CAG CTC 3'	HYB-MTV 1433-1480	DA024
	OTR 1405		S	5' GTA GGT TAC GGT GAG CCA TT 3'	HYB-MTV 1461-1480	
AK50	OTR 1402	ssPurines	AS	5' AAT GGC TCA CCG TAA CCT ACC TGA ACT CCG TAG GCG GGA CTG CAG CTC 3'	HYB-MTV 1433-1480	DA024
	OTR 1405		S	5' GTA GGT TAC GGT GAG CCA TT 3'	HYB-MTV 1461-1480	

Supplementary Table 1 (continued)

Primers used for introduction of mutations and construction of clones						
**Mutation Names**	**Primers**	**Region where mutations were introduced/ description**	***S or AS**	**Sequence**	**Nucleotide position**	**Starting plasmid**
AK62	OTR 1414	ssPurines	AS	5’ AAT GGC TCA CCG TAA CCT ACG ACT TCT CCG TAG GCG GGA CTG CAG CTC 3’	HYB-MTV 1433- 480	DA024
	OTR 1405		S	5’ GTA GGT TAC GGT GAG CCA TT 3’	HYB-MTV 1461-1480	
AK63	OTR 1413	ssPurines	AS	5’ AAT GGC TCA CCG TAA CCT ACC TCT TGA GGG TAG GCG GGA CTG CAG CTC 3’	HYB-MTV 1433-1480	DA024
	OTR 1405		S	5’ GTA GGT TAC GGT GAG CCA TT 3’	HYB-MTV 1461-1480	
AK18	OTR 1035	ssPurines	AS	5' CCG TAA CCT ACG AGA AGA GGG TAG GCG GGA CTG CAG CTC C 3'	HYB-MTV 1451-1432	DA024
	OTR 1034		S	5' CCT CTT CTC GTA GGT TAC GGT GAG CCA TTG G 3'	HYB-MTV 1471-1461	
AK29	OTR 1371	Amplifying from R to mSD	AS	5’ GAT TGG TGT TTC GGC ATC CTC TTC TCC GTA GGC GGG 3’	HYB-MTV 1443-1460 & 6535-6552	HYB- MTV
	OTR 1370	Amplifying from *env* splice acceptor to 424 bp of *env*	S	5’ GAT GCC GAA ACA CCA ATC TG 3’	HYB-MTV 6535-6554	
	OTR 1372		AS	5’ aaa **ccc ggg** TAA ACC CGT GAA AGT CAG GC 3’	HYB-MTV 6940-6959	
AK30	OTR 1374	Amplifying from R to mSD	AS	5’ AGG CTC TTC GCA AGG CAC TCT TCT CCG TAG GCG GG 3’	HYB-MTV 1443-1460 & 8477-8493	HYB- MTV
	OTR 1373	Amplifying from *sag* splice acceptor to 351 bp of *sag*	S	5’ TGC CTT GCG AAG AGC CTT G 3’	HYB-MTV 8477-8495	
	OTR 1375		AS	5’ aaa **ccc ggg** TTT CTG AAG GAC AAA ATC GAT G 3’	HYB-MTV 8880-8901	

Supplementary Table 1 (continued)

Primers used for introduction of mutations and construction of clones					
**Primers**	**Region where mutations were introduced/ description**	***S or AS**	**Sequence**	**Nucleotide position**	**Starting plasmid**
OTR 249	Outer primers used for construction of subgenomic transfer vectors	S	5’ CC GCT AGC CTT CGC GAT GTA CGG GCC AGA 3’	pCDNA3 204-224	First round amplification products in the SOE PCR
OTR 552		AS	5' cg **act agt gat atc** GTT CCC CTG GTC CCA T 3'	HYB-MTV 1885-1867	
OTR 984	Primers used for construction of *in vitro* transcribing clones	S	5’ ccc **aag ctt** AAT ACG ACT CAC TAT AGG GCA ACA GTC CTA ATA TTC ACG 3'	HYB-MTV 1173-1193	DA024/ its mutants
OTR 985		AS	5’ aaa **ccc ggg** TTC CCC TGG TCC CAT AAG 3’	HYB-MTV 1885-1867	

Supplementary Table 1 (continued)

Primers used for SHAPE, RT-qPCR and other amplifications				
**Primer**	**Description**	***S or AS**	**Sequence**	**Nucleotide position or reference**
OTR 10	Labelled primers used for SHAPE analysis of WT (SA35)	AS	VIC - 5' AACAGATTTGGCTTCTGCGG 3'	HYB-MTV 1786-1805
OTR 11		AS	NED - 5' AACAGATTTGGCTTCTGCGG 3'	HYB-MTV 1786-1805
OTR 14		AS	VIC- 5' AGTTTCTGCCCTTTTGAGCC 3'	HYB-MTV 1497-1516
OTR 15		AS	NED - 5' AGTTTCTGCCCTTTTGAGCC 3'	HYB-MTV 1497-1516
Splice _1_1	Labelled primers used for SHAPE analysis of *sag* RNA (AK30)	AS	VIC- 5' GCTCTTGTGATGATAGCCAG 3'	HYB-MTV 1394-1413
Splice _1_2		AS	NED- 5' GCTCTTGTGATGATAGCCAG 3'	
Splice_2_1		AS	VIC- 5' CTATGCCAAGTTTGCAGCAG 3'	HYB-MTV 8693-8712
Splice_2_2		AS	NED- 5' CTATGCCAAGTTTGCAGCAG 3'	
Splice _1_1	Labelled primers used for SHAPE analysis of *env* RNA (AK29)	AS	VIC- 5' GCTCTTGTGATGATAGCCAG 3'	HYB-MTV 1394-1413
Splice _1_2		AS	NED- 5' GCTCTTGTGATGATAGCCAG 3'	
Splice_3_1		AS	VIC- 5' GGTTTTAAGAACCTCCTCCG 3'	HYB-MTV 6735-6754
Splice_3_2		AS	NED- 5' GGTTTTAAGAACCTCCTCCG 3'	
OTR 580	β-actin spliced mRNA	S	5' TGA GCT GCG TGT GGC TCC 3'	(Tan *et al.*, 1995)
OTR 581	β-actin spliced or unspliced mRNA	AS	5' GGC ATG GGG GAG GGC ATA CC 3'	
OTR 582	β-actin unspliced mRNA	S	5' CCA GTG GCT TCC CCA GTG 3'	
OTR 1391	Vector specific	S	5' GTC CTA ATA TTC ACG TCT CGT GTG 3'	HYB-MTV 1216-1233

Supplementary Table 1 (continued)

Primers used for SHAPE, RT-qPCR and other amplifications				
**Primer**	**Description**	***S or AS**	**Sequence**	**Nucleotide position or reference**
OTR 1392	Vector specific	AS	5' CTG TTC GGG CGC CAG CTG CCG CAG 3'	HYB- MTV 1364-1385
MTV-1LTR - SITEM1 FAM	qPCR probe	Probe	FAM- 5' TCG CCA TCC CGT CTC C 3'	HYB-MTV 1214-1229
MTV- 1LTR -SITEF	qPCR Forward primer	S	5' CGT CTC GTG TGT TTG TGT TTG TGT CTG T 3'	HYB-MTV 1192-1213
MTV-1LTR -SITER	qPCR Reverse Primer	AS	5' CCT CTG GAA AGT GAA GGA TAA GTG A 3'	HYB-MTV 1259-1235

AAT ACG ACT CAC TAT AGG; T7 Promoter

Sequence in lower case; dummy sequences that were introduced in the oligos.

Sequence in lower case and bold; restriction enzyme sequences that were introduced in the oligos.

*S, sense; AS, antisense.

References

1) Aktar,S.J., Vivet-Boudou,V., Ali,L.M., Jabeen,A., Kalloush,R.M., Richer,D., Mustafa,F., Marquet,R. and Rizvi,T.A. (2014) Structural basis of genomic RNA (gRNA) dimerization and packaging determinants of mouse mammary tumor virus (MMTV). *Retrovirology*, **11**, 96

2) Tan, Wei, Barbara K. Felber, Andrei S. Zolotukhin, George N. Pavlakis, and Stefan Schwartz. (1995) Efficient expression of the human papillomavirus type 16 L1 protein in epithelial cells by using Rev and the Rev-responsive element of human immunodeficiency virus or the cis-acting transactivation element of simian retrovirus type 1." *Journal of virology,* **69**, 9
