# Supplementary material for: A purine loop and the primer binding site are critical for the selective encapsidation of mouse mammary tumor virus genomic RNA by Pr77Gag
Source: Nucleic Acids Res. 2021 Apr 9;49(8):4668–88. doi: 10.1093/nar/gkab223 (PMC8096270; doi:10.1093/nar/gkab223)
Supplement: gkab223_Supplemental_Files [file gkab223_supplemental_files.zip › Supplementary Table 2.pdf]

Supplementary Table 2 (pages 1-11)

AK29/*env* mRNA				AK30/*sag* mRNA			
**Nucleotide number & sequence**		**Mean**	**SD**	**Nucleotide number & sequence**		**Mean**	**SD**
1	G	-999	0	1	G	-999	0
2	C	-999	0	2	C	-999	0
3	A	-999	0	3	A	-999	0
4	A	-999	0	4	A	-999	0
5	C	-999	0	5	C	-999	0
6	A	-999	0	6	A	-999	0
7	G	-999	0	7	G	-999	0
8	U	-999	0	8	U	-999	0
9	C	0.3025	0.3391	9	C	-999	0
10	C	0.0000	0.0000	10	C	-999	0
11	U	0.4425	0.2912	11	U	0.6500	0.3054
12	A	0.5100	0.1995	12	A	0.3500	0.1594
13	A	0.9575	0.2722	13	A	0.6000	0.1558
14	U	0.9950	0.2659	14	U	0.8800	0.0787
15	A	2.4625	0.7947	15	A	2.2125	0.6873
16	U	1.5575	0.4891	16	U	1.5550	0.2515
17	U	0.5900	0.1865	17	U	0.6250	0.1190
18	C	0.2825	0.1959	18	C	0.0975	0.1127
19	A	0.2900	0.2230	19	A	0.2650	0.1100
20	C	0.0100	0.0082	20	C	0.0600	0.0392
21	G	0.3975	0.1372	21	G	0.5000	0.1225
22	U	1.9875	0.5611	22	U	2.1050	0.2689
23	C	0.6125	0.2445	23	C	0.8150	0.1873
24	U	2.0950	0.5186	24	U	2.1850	0.3074
25	C	0.6100	0.2467	25	C	0.7225	0.1075
26	G	0.0175	0.0287	26	G	0.0725	0.0645
27	U	0.0050	0.0100	27	U	0.0350	0.0436
28	G	0.0125	0.0250	28	G	0.0450	0.0900
29	U	0.3750	0.0843	29	U	0.0600	0.0365
30	G	0.4775	0.1075	30	G	0.1000	0.0688
31	U	0.3375	0.0629	31	U	0.2200	0.0648
32	U	0.6650	0.1498	32	U	0.2175	0.0873
33	U	0.3925	0.0998	33	U	0.2250	0.0733
34	G	0.1575	0.0472	34	G	0.2750	0.0619
35	U	1.1800	0.3455	35	U	1.2125	0.2389
36	G	0.0750	0.0420	36	G	0.1550	0.0387
37	U	0.0075	0.0150	37	U	0.0375	0.0287
38	C	0.0000	0.0000	38	C	0.0000	0.0000

Supplementary Table 2 (continued)

AK29/*env* mRNA				AK30/*sag* mRNA			
Nucleotide number & sequence		Mean	SD	Nucleotide number & sequence		Mean	SD
39	U	0.0100	0.0200	39	U	0.0025	0.0050
40	G	0.0025	0.0050	40	G	0.0000	0.0000
41	U	0.0000	0.0000	41	U	0.0000	0.0000
42	U	0.0850	0.1066	42	U	0.0200	0.0283
43	C	0.1700	0.1140	43	C	0.0750	0.0881
44	G	0.0000	0.0000	44	G	0.0000	0.0000
45	C	0.0000	0.0000	45	C	0.0425	0.0850
46	C	0.1000	0.2000	46	C	0.3200	0.6400
47	A	1.8675	0.5982	47	A	2.3950	0.6409
48	U	0.9625	0.3280	48	U	1.3250	0.1865
49	C	0.0775	0.0556	49	C	0.2525	0.1526
50	C	0.0000	0.0000	50	C	0.0000	0.0000
51	C	0.0000	0.0000	51	C	0.0025	0.0050
52	G	0.0700	0.1334	52	G	0.0575	0.0465
53	U	0.1225	0.0457	53	U	0.1125	0.0250
54	C	0.2300	0.1111	54	C	0.2825	0.0655
55	U	0.0000	0.0000	55	U	0.0025	0.0050
56	C	0.0000	0.0000	56	C	0.0000	0.0000
57	C	0.0000	0.0000	57	C	0.0000	0.0000
58	G	0.0175	0.0350	58	G	0.0125	0.0250
59	C	0.0250	0.0500	59	C	0.0225	0.0450
60	U	0.5100	0.2340	60	U	0.3250	0.0480
61	C	0.1125	0.0450	61	C	0.2300	0.0548
62	G	0.0000	0.0000	62	G	0.0000	0.0000
63	U	0.0550	0.0971	63	U	0.0525	0.0640
64	C	0.7325	0.5016	64	C	0.5000	0.5788
65	A	0.4425	0.1255	65	A	0.2425	0.1087
66	C	0.0125	0.0250	66	C	0.0175	0.0171
67	U	0.3325	0.1109	67	U	0.2775	0.0330
68	U	0.2600	0.3308	68	U	0.2850	0.2310
69	A	0.4925	0.1678	69	A	0.5025	0.0695
70	U	0.2475	0.1100	70	U	0.2700	0.0337
71	C	0.0550	0.0911	71	C	0.0500	0.0577
72	C	0.0475	0.0550	72	C	0.0950	0.0954
73	U	0.9350	0.2173	73	U	1.0275	0.2326
74	U	1.3225	0.2234	74	U	1.0575	0.3489
75	C	0.6975	0.6551	75	C	0.6400	0.7812
76	A	1.4675	0.4583	76	A	1.4950	0.4671

Supplementary Table 2 (continued)

AK29/*env* mRNA				AK30/*sag* mRNA			
Nucleotide number & sequence		Mean	SD	Nucleotide number & sequence		Mean	SD
77	C	0.3400	0.1433	77	C	0.4225	0.1830
78	U	1.4075	0.6649	78	U	1.5675	0.4740
79	U	1.7600	0.6639	79	U	1.9550	0.5814
80	U	0.8300	0.3556	80	U	0.9050	0.1942
81	C	0.0725	0.0797	81	C	0.0750	0.0957
82	C	0.4775	0.9550	82	C	0.3475	0.5638
83	A	1.1375	0.9932	83	A	0.5475	0.8918
84	G	1.5475	0.8387	84	G	1.7050	0.2076
85	A	0.4475	0.3435	85	A	0.2925	0.1053
86	G	0.1625	0.2803	86	G	0.2050	0.2762
87	G	0.0000	0.0000	87	G	0.2450	0.3489
88	G	0.0325	0.0650	88	G	0.0825	0.1650
89	U	0.0000	0.0000	89	U	0.0050	0.0100
90	C	0.0025	0.0050	90	C	0.0150	0.0300
91	C	0.0375	0.0450	91	C	0.0450	0.0772
92	C	0.0675	0.0780	92	C	0.1750	0.1546
93	C	0.0350	0.0700	93	C	0.0275	0.0550
94	C	0.0175	0.0350	94	C	0.0475	0.0950
95	C	0.0275	0.0550	95	C	0.0750	0.0500
96	G	0.0300	0.0535	96	G	0.1100	0.0141
97	C	0.0600	0.1200	97	C	0.0900	0.1023
98	A	0.3375	0.2089	98	A	0.3675	0.0946
99	G	0.5325	0.1839	99	G	0.5800	0.1102
100	A	1.6725	0.5867	100	A	1.5775	0.2980
101	C	0.1200	0.0606	101	C	0.1475	0.0556
102	C	0.1325	0.1247	102	C	0.2300	0.1829
103	C	0.3150	0.3964	103	C	0.6625	0.3340
104	C	0.0050	0.0100	104	C	0.0500	0.0594
105	G	0.0000	0.0000	105	G	0.0075	0.0150
106	G	0.0000	0.0000	106	G	0.0350	0.0473
107	U	0.1200	0.0920	107	U	0.1400	0.0356
108	G	0.0475	0.0403	108	G	0.0325	0.0359
109	A	0.0650	0.0569	109	A	0.0475	0.0150
110	C	0.0000	0.0000	110	C	0.0050	0.0100
111	C	0.1750	0.0915	111	C	0.1800	0.0796
112	C	0.6875	0.2885	112	C	0.8550	0.1139
113	U	2.7375	0.8160	113	U	2.5350	0.2659
114	C	1.1850	0.3022	114	C	1.0350	0.3795

Supplementary Table 2 (continued)

AK29/*env* mRNA				AK30/*sag* mRNA			
Nucleotide number & sequence		Mean	SD	Nucleotide number & sequence		Mean	SD
115	A	3.4175	0.8825	115	A	3.1675	0.5784
116	G	0.1375	0.1187	116	G	0.1400	0.0383
117	G	0.0400	0.0497	117	G	0.0800	0.0294
118	U	0.0775	0.0699	118	U	0.1150	0.0191
119	C	0.1575	0.2559	119	C	0.0475	0.0250
120	G	0.5650	0.1790	120	G	0.4925	0.1513
121	G	0.7350	0.5208	121	G	0.9600	0.1249
122	C	0.0125	0.0250	122	C	0.0150	0.0238
123	C	0.0400	0.0800	123	C	0.0025	0.0050
124	G	0.1500	0.1192	124	G	0.1850	0.0614
125	A	0.3250	0.1103	125	A	0.3275	0.0538
126	C	0.0275	0.0263	126	C	0.0625	0.0310
127	U	0.0275	0.0340	127	U	0.0700	0.0337
128	G	0.0250	0.0300	128	G	0.0575	0.0206
129	C	0.0000	0.0000	129	C	0.0000	0.0000
130	G	0.0200	0.0400	130	G	0.0050	0.0100
131	G	0.0025	0.0050	131	G	0.0000	0.0000
132	C	0.3100	0.6200	132	C	0.3125	0.3709
133	A	0.0050	0.0100	133	A	0.1525	0.2035
134	G	0.9975	0.7101	134	G	0.8675	0.7830
135	C	0.7600	0.7869	135	C	0.3300	0.2232
136	U	1.2250	0.6268	136	U	1.5050	0.2999
137	G	0.3100	0.0622	137	G	0.2875	0.0750
138	G	0.1650	0.3300	138	G	0.2050	0.1091
139	C	0.1125	0.0096	139	C	0.1425	0.0640
140	G	0.1275	0.0377	140	G	0.1375	0.0287
141	C	0.0000	0.0000	141	C	0.0050	0.0100
142	C	0.0550	0.1100	142	C	0.1750	0.0759
143	C	0.2000	0.1822	143	C	0.3925	0.2103
144	G	0.6975	0.1473	144	G	0.7825	0.1391
145	A	1.0950	0.2745	145	A	1.2475	0.2526
146	A	0.7550	0.5683	146	A	0.7375	0.4988
147	C	0.9625	0.6547	147	C	0.9500	1.0970
148	A	0.0275	0.0550	148	A	0.0625	0.1250
149	G	0.0000	0.0000	149	G	0.0000	0.0000
150	G	0.0000	0.0000	150	G	0.0975	0.1950
151	G	0.0000	0.0000	151	G	0.1275	0.2550
152	A	0.0000	0.0000	152	A	0.0225	0.0450

Supplementary Table 2 (continued)

AK29/*env* mRNA				AK30/*sag* mRNA			
Nucleotide number & sequence		Mean	SD	Nucleotide number & sequence		Mean	SD
153	C	0.0475	0.0950	153	C	0.8975	1.6452
154	C	2.1725	2.5086	154	C	0.3475	0.5638
155	C	0.1425	0.2850	155	C	0.7275	0.5416
156	U	0.1650	0.3300	156	U	0.4050	0.4853
157	C	0.1625	0.1752	157	C	0.3950	0.3287
158	G	0.1300	0.2600	158	G	0.2500	0.4609
159	G	0.0525	0.1050	159	G	0.0950	0.1769
160	A	0.0900	0.0883	160	A	0.1500	0.0141
161	U	0.3300	0.2080	161	U	0.3375	0.1905
162	A	0.0900	0.0753	162	A	0.0775	0.0763
163	A	0.0475	0.0320	163	A	0.0175	0.0222
164	G	0.0175	0.0350	164	G	0.0075	0.0150
165	U	0.1050	0.2100	165	U	0.0325	0.0340
166	G	0.0250	0.0500	166	G	0.0575	0.0386
167	A	0.0550	0.0802	167	A	0.1950	0.1115
168	C	0.1575	0.1819	168	C	0.4150	0.3373
169	C	0.1450	0.1127	169	C	0.1700	0.1831
170	C	0.1350	0.0926	170	C	0.1825	0.1758
171	U	0.6225	0.0189	171	U	0.5200	0.0707
172	U	0.3675	0.1619	172	U	0.2675	0.1090
173	G	0.3100	0.1407	173	G	0.2825	0.0922
174	U	0.3200	0.0183	174	U	0.2550	0.0705
175	C	0.1325	0.1031	175	C	0.0825	0.0929
176	U	0.4125	0.1750	176	U	0.3625	0.2541
177	C	0.1925	0.1394	177	C	0.2400	0.1757
178	U	0.5425	0.5803	178	U	0.7350	0.5244
179	A	0.8475	0.0709	179	A	0.7350	0.1852
180	U	0.3975	0.0591	180	U	0.4000	0.0949
181	U	0.5775	0.0854	181	U	0.5200	0.0490
182	U	0.3300	0.0638	182	U	0.2750	0.1589
183	C	0.1600	0.1010	183	C	0.1900	0.1719
184	U	0.5275	0.4565	184	U	0.6100	0.4399
185	A	0.6025	0.1090	185	A	0.4500	0.3014
186	C	0.2500	0.2501	186	C	0.1725	0.1424
187	U	0.4525	0.3676	187	U	0.3075	0.2341
188	A	0.7000	0.2820	188	A	0.4950	0.2053
189	U	0.2200	0.1030	189	U	0.2325	0.1269
190	U	0.1600	0.1117	190	U	0.2175	0.0896

Supplementary Table 2 (continued)

AK29/*env* mRNA				AK30/*sag* mRNA			
Nucleotide number & sequence		Mean	SD	Nucleotide number & sequence		Mean	SD
191	U	0.1700	0.0938	191	U	0.1475	0.0768
192	G	0.0400	0.0497	192	G	0.0600	0.0735
193	G	0.0000	0.0000	193	G	0.0000	0.0000
194	U	0.0025	0.0050	194	U	0.0000	0.0000
195	G	0.0050	0.0100	195	G	0.0050	0.0100
196	U	0.2350	0.1047	196	U	0.1400	0.0787
197	U	0.3275	0.1289	197	U	0.4050	0.0900
198	U	0.3550	0.1678	198	U	0.4350	0.1085
199	G	0.2375	0.1826	199	G	0.0075	0.0150
200	U	0.1875	0.1167	200	U	0.0125	0.0250
201	C	0.0100	0.0200	201	C	0.0050	0.0100
202	U	0.3700	0.1655	202	U	0.0750	0.1136
203	U	0.8625	0.2775	203	U	0.5350	0.1950
204	G	0.1300	0.0408	204	G	0.0350	0.0700
205	U	0.2450	0.3188	205	U	0.3950	0.2124
206	A	0.3275	0.0608	206	A	0.2675	0.1132
207	U	0.2975	0.1184	207	U	0.2575	0.0250
208	U	0.3975	0.2211	208	U	0.1400	0.0583
209	G	0.7950	0.3783	209	G	0.0350	0.0507
210	U	0.4025	0.0881	210	U	0.3150	0.2453
211	C	0.0000	0.0000	211	C	0.0000	0.0000
212	U	0.0000	0.0000	212	U	0.0000	0.0000
213	C	0.0000	0.0000	213	C	0.0000	0.0000
214	U	0.0000	0.0000	214	U	0.0475	0.0550
215	U	0.1725	0.1087	215	U	0.3200	0.0739
216	U	0.2750	0.1162	216	U	0.3350	0.0843
217	C	0.0225	0.0450	217	C	0.0200	0.0245
218	U	0.2275	0.0943	218	U	0.2350	0.1066
219	U	0.2800	0.0762	219	U	0.2275	0.1072
220	G	0.3425	0.0780	220	G	0.2250	0.0557
221	U	0.1600	0.0316	221	U	0.1150	0.0850
222	C	0.1525	0.0776	222	C	0.1300	0.0294
223	U	0.3200	0.0638	223	U	0.1275	0.0562
224	G	0.3825	0.0826	224	G	0.2050	0.1269
225	G	0.0850	0.0661	225	G	0.0225	0.0450
226	C	0.1875	0.2156	226	C	0.1050	0.1085
227	U	1.3900	0.3710	227	U	1.2175	0.3657
228	A	1.3700	0.1883	228	A	1.2400	0.1641

Supplementary Table 2 (continued)

AK29/*env* mRNA				AK30/*sag* mRNA			
Nucleotide number & sequence		Mean	SD	Nucleotide number & sequence		Mean	SD
229	U	1.3575	0.1209	229	U	1.1275	0.2555
230	C	0.6650	1.0812	230	C	0.6500	0.3599
231	A	0.8150	0.2068	231	A	0.7300	0.2162
232	U	0.5375	0.1159	232	U	0.4500	0.1857
233	C	0.7450	1.1778	233	C	0.4900	0.3472
234	A	0.8400	0.2102	234	A	0.7825	0.2870
235	C	1.6475	2.9075	235	C	0.7000	0.5141
236	A	0.6600	0.2789	236	A	0.7775	0.3425
237	A	0.7500	0.1598	237	A	0.8625	0.2783
238	G	0.1450	0.0545	238	G	0.0100	0.0200
239	A	0.1650	0.0289	239	A	0.1400	0.0141
240	G	0.0125	0.0189	240	G	0.0875	0.0299
241	C	0.0300	0.0216	241	C	0.0725	0.1384
242	G	0.0425	0.0723	242	G	0.0600	0.1200
243	G	0.1525	0.0954	243	G	0.1600	0.1071
244	A	0.3800	0.1806	244	A	0.3350	0.1204
245	A	0.1600	0.0294	245	A	0.1175	0.0680
246	C	0.0450	0.0614	246	C	0.0000	0.0000
247	G	0.1025	0.1617	247	G	0.0000	0.0000
248	G	0.0450	0.0714	248	G	0.0000	0.0000
249	A	0.4150	0.1535	249	A	0.5425	0.2207
250	C	0.0475	0.0763	250	C	0.0550	0.1100
251	U	0.2875	0.2500	251	U	0.1275	0.0873
252	C	1.3175	1.9319	252	C	1.0575	0.3951
253	A	0.8225	0.1059	253	A	0.4925	0.1245
254	C	0.0900	0.1800	254	C	0.0000	0.0000
255	C	1.8125	3.5191	255	C	0.2700	0.4175
256	A	1.5575	0.6464	256	A	0.8675	0.3398
257	U	1.2975	0.6992	257	U	1.1625	0.4132
258	A	2.1950	0.1921	258	A	1.2675	0.3404
259	G	0.4375	0.0806	259	G	0.4525	0.1424
260	G	0.1200	0.0346	260	G	0.0925	0.0330
261	G	0.0275	0.0427	261	G	0.1425	0.0690
262	A	0.2125	0.0472	262	A	0.4275	0.1218
263	G	0.0650	0.0480	263	G	0.0000	0.0000
264	C	0.0475	0.0330	264	C	0.1250	0.1893
265	U	0.3625	0.1819	265	U	0.1300	0.1061
266	G	0.7850	0.2601	266	G	0.2650	0.1292

Supplementary Table 2 (continued)

AK29/*env* mRNA				AK30/*sag* mRNA			
Nucleotide number & sequence		Mean	SD	Nucleotide number & sequence		Mean	SD
267	C	0.4175	0.4007	267	C	0.2150	0.1245
268	A	1.0525	0.3371	268	A	0.3175	0.1799
269	G	0.3025	0.3597	269	G	0.1800	0.0804
270	U	0.3525	0.1678	270	U	0.1550	0.0742
271	C	0.0100	0.0200	271	C	0.0050	0.0100
272	C	0.0250	0.0500	272	C	0.0000	0.0000
273	C	0.0100	0.0200	273	C	0.3275	0.3093
274	G	0.1150	0.0777	274	G	1.2725	0.1181
275	C	0.1875	0.1839	275	C	0.2175	0.1739
276	C	0.3850	0.1870	276	C	0.0800	0.0294
277	U	0.5200	0.2920	277	U	0.3675	0.0411
278	A	1.1075	0.1905	278	A	0.5375	0.1112
279	C	0.5000	0.1512	279	C	0.3875	0.1059
280	G	0.0725	0.0330	280	G	0.0900	0.0707
281	G	0.0600	0.0632	281	G	0.1550	0.1406
282	A	0.2575	0.1153	282	A	0.3900	0.4318
283	G	0.0850	0.0790	283	G	0.0750	0.0926
284	A	0.2200	0.0942	284	A	0.3150	0.0975
285	A	0.2550	0.0420	285	A	0.4575	0.1664
286	G	0.0575	0.0704	286	G	0.0600	0.1200
287	A	0.4675	0.0525	287	A	0.4600	0.3151
288	G	0.0100	0.0141	288	G	0.4575	0.2455
289	G	0.0000	0.0000	289	U	0.2200	0.1545
290	A	0.0925	0.1069	290	G	0.3550	0.1038
291	U	0.0450	0.0173	291	C	0.1875	0.1410
292	G	0.0400	0.0163	292	C	0.0250	0.0300
293	C	0.0425	0.0723	293	U	0.0450	0.0835
294	C	0.0550	0.0681	294	U	0.0525	0.0377
295	G	0.2825	0.0699	295	G	0.3725	0.0403
296	A	0.4025	0.1408	296	C	0.4800	0.1669
297	A	0.3675	0.0746	297	G	0.1925	0.0435
298	A	0.4225	0.0350	298	A	0.3050	0.0420
299	C	0.1725	0.3450	299	A	0.1300	0.1010
300	A	0.1975	0.0991	300	G	0.2400	0.0408
301	C	0.0175	0.0350	301	A	0.7225	0.1396
302	C	0.1500	0.3000	302	G	0.9575	0.1987
303	A	0.2675	0.3021	303	C	0.2600	0.0909
304	A	1.9200	0.3721	304	C	0.1650	0.0874

Supplementary Table 2 (continued)

**AK29/*env* mRNA**				**AK30/*sag* mRNA**			
**Nucleotide number & sequence**		**Mean**	**SD**	**Nucleotide number & sequence**		**Mean**	**SD**
305	U	0.6975	0.1081	305	U	1.0400	0.2981
306	C	0.6425	0.1357	306	U	0.8850	0.2469
307	U	1.3425	0.2277	307	G	0.9275	0.4509
308	G	0.5075	0.0896	308	A	1.3350	0.2428
309	G	0.0350	0.0700	309	C	0.0600	0.0952
310	G	0.0875	0.0660	310	C	0.1225	0.2450
311	U	0.1550	0.1012	311	A	0.0475	0.0763
312	C	0.0075	0.0150	312	A	0.1250	0.0819
313	C	0.0275	0.0550	313	G	0.1400	0.0548
314	C	0.1475	0.2950	314	U	0.3650	0.0603
315	C	0.1125	0.2250	315	G	0.0025	0.0050
316	G	0.1875	0.2237	316	C	0.0575	0.0802
317	A	0.8150	0.0835	317	A	1.3425	0.2435
318	U	0.0500	0.0577	318	G	0.6425	0.2855
319	C	0.0000	0.0000	319	U	0.5325	0.2926
320	G	0.0500	0.1000	320	C	0.2000	0.2828
321	G	0.1525	0.1644	321	A	0.2400	0.1691
322	U	0.3650	0.1218	322	G	0.5375	0.3804
323	U	0.6950	0.1015	323	A	0.8325	0.2061
324	C	0.6475	1.1844	324	U	0.3725	0.2037
325	A	0.3000	0.1402	325	C	0.1600	0.1236
326	U	0.0675	0.0359	326	U	0.5200	0.1080
327	C	0.0075	0.0150	327	U	1.0325	0.7293
328	C	0.0450	0.0772	328	A	0.7000	0.1645
329	G	0.2625	0.1611	329	A	0.6175	0.1001
330	A	1.5825	0.1914	330	C	0.3700	0.3209
331	C	0.1925	0.0386	331	G	0.3925	0.0544
332	C	0.0725	0.0763	332	U	0.6375	0.0810
333	U	0.7325	0.2920	333	G	0.8225	0.2356
334	U	0.9475	0.2016	334	C	0.1425	0.1590
335	U	1.3300	0.1679	335	U	0.4025	0.3369
336	U	2.7650	0.6488	336	U	0.2675	0.1646
337	A	0.7350	0.1473	337	C	0.1900	0.1647
338	C	0.2700	0.1105	338	U	1.0975	0.6766
339	U	0.6575	0.1473	339	U	0.4275	0.0922
340	G	0.5875	0.1788	340	U	1.3200	0.0529
341	A	0.4050	0.1279	341	U	0.8350	0.4994
342	G	0.0175	0.0206	342	A	0.4800	0.2734

Supplementary Table 2 (continued)

AK29/*env* mRNA				AK30/*sag* mRNA			
Nucleotide number & sequence		Mean	SD	Nucleotide number & sequence		Mean	SD
343	C	0.0150	0.0173	343	A	0.9325	0.0411
344	G	0.0000	0.0000	344	A	0.9775	0.2572
345	G	0.0200	0.0400	345	A	0.5550	0.2133
346	A	0.4425	0.1491	346	A	0.1200	0.1169
347	A	0.9950	0.1518	347	A	0.2200	0.1068
348	A	1.2075	0.0818	348	G	0.2350	0.0614
349	G	0.8850	0.0806	349	A	0.0650	0.0896
350	A	0.9775	0.2792	350	A	0.3300	0.0852
351	A	0.8200	0.0668	351	A	1.2825	0.4192
352	G	0.2025	0.3299	352	A	0.6775	0.1615
353	C	0.8575	1.7150	353	A	0.8500	0.2586
354	A	0.4700	0.3782	354	A	0.4200	0.1449
355	A	0.4200	0.0606	355	G	0.0050	0.0100
356	C	0.1700	0.0408	356	G	0.0075	0.0150
357	G	0.0850	0.0493	357	G	0.1175	0.2350
358	C	0.0000	0.0000	358	G	0.0775	0.1550
359	C	0.0000	0.0000	359	G	0.1450	0.2900
360	C	0.0000	0.0000	360	A	0.3500	0.7000
361	A	0.8575	0.2935	361	A	0.5300	0.3366
362	C	0.8700	1.5465	362	A	1.6550	0.2931
363	A	1.1975	0.3395	363	U	1.8050	0.3142
364	C	0.0750	0.1500	364	G	0.3650	0.2004
365	C	0.1400	0.1241	365	C	0.0000	0.0000
366	U	0.1725	0.1717	366	C	0.0000	0.0000
367	G	0.0000	0.0000	367	G	0.2700	0.1691
368	G	0.0475	0.0950	368	C	0.3100	0.2189
369	C	0.9500	1.9000	369	G	0.5425	0.1338
370	A	0.4175	0.2193	370	C	0.0600	0.0455
371	C	0.1500	0.0913	371	C	0.0975	0.0903
372	U	0.0675	0.0532	372	U	0.2400	0.0245
373	G	0.0550	0.0451	373	G	0.1250	0.0404
374	C	0.0225	0.0287	374	C	1.1750	0.5459
375	G	0.0200	0.0400	375	A	1.4450	0.0173
376	G	0.1625	0.1535	376	G	0.0025	0.0050
377	A	0.6725	0.3041	377	C	0.0000	0.0000
378	G	0.5950	0.5087	378	A	0.0000	0.0000
379	A	1.5150	0.3739	379	G	0.0000	0.0000
380	A	0.0850	0.0532	380	A	0.5100	0.1924

Supplementary Table 2 (continued)

AK29/*env* mRNA				AK30/*sag* mRNA			
Nucleotide number & sequence		Mean	SD	Nucleotide number & sequence		Mean	SD
381	A	1.4125	0.3289	381	A	0.7150	0.1515
382	A	1.1650	0.3193	382	A	1.3800	0.1802
383	C	1.3425	0.2707	383	U	1.0175	0.4046
384	G	0.0475	0.0411	384	G	0.0000	0.0000
385	C	0.0000	0.0000	385	G	0.0000	0.0000
386	C	0.0000	0.0000	386	U	0.0450	0.0465
387	G	0.0000	0.0000	387	U	0.0850	0.1179
388	C	0.0100	0.0200	388	G	0.1975	0.0723
389	C	0.0900	0.0589	389	A	0.6350	0.1881
390	G	0.1825	0.0386	390	A	0.7950	0.2034
391	C	0.3525	0.0846	391	C	0.0525	0.0443
392	G	0.8300	0.2368	392	U	0.0000	0.0000
393	A	0.3550	0.1072	393	C	0.0800	0.0956
394	G	0.0400	0.0566	394	C	0.0000	0.0000
395	A	0.1275	0.0377	395	C	0.0000	0.0000
396	U	0.1100	0.0469	396	G	0.2950	0.1271
397	G	0.0950	0.0387	397	A	0.4975	0.3597
398	A	0.4350	0.1139	398	G	1.0250	0.2193
399	G	0.5875	0.0954	399	A	2.6075	0.6607
400	A	0.2325	0.0359	400	G	0.8200	1.6400
401	A	0.2375	0.0330	401	U	0.0850	0.0603
402	A	0.3600	0.0455	402	G	0.1900	0.1049
403	G	0.2400	0.0753	403	U	1.7625	0.4518
404	A	0.6775	0.1024	404	C	0.3700	0.3136
405	U	0.6525	0.2090	405	C	0.6050	0.2533
406	C	0.8925	1.7850	406	U	2.7550	1.1024
407	A	0.6775	0.1975	407	A	0.6275	0.0591
408	A	0.4625	0.1994	408	C	0.0000	0.0000
409	C	0.6850	1.3700	409	A	0.0500	0.1000
410	A	0.1225	0.1109	410	C	0.0300	0.0535
411	G	0.0625	0.0793	411	C	0.0450	0.0900
412	A	0.0375	0.0750	412	U	0.2700	0.5400
413	A	0.1225	0.2130	413	A	0.8150	0.1136
414	A	0.2175	0.1159	414	G	1.8875	0.4711
415	A	0.2850	0.0723	415	G	0.0000	0.0000
416	G	0.6175	0.1443	416	G	0.0000	0.0000
417	U	0.2300	0.0337	417	G	0.0000	0.0000
418	C	0.0350	0.0635	418	A	0.2900	0.0966

Supplementary Table 2 (continued)

**AK29/*env* mRNA**				**AK30/*sag* mRNA**			
**Nucleotide number & sequence**		**Mean**	**SD**	**Nucleotide number & sequence**		**Mean**	**SD**
419	C	0.1425	0.0189	419	G	0.0175	0.0236
420	G	0.3225	0.0532	420	A	1.1550	0.3854
421	G	0.0475	0.0512	421	A	1.1975	0.1473
422	A	0.0550	0.1100	422	G	0.3475	0.1524
423	G	0.0000	0.0000	423	C	0.1200	0.2400
424	G	0.0000	0.0000	424	A	0.1750	0.3500
425	A	0.0000	0.0000	425	G	0.0000	0.0000
426	U	0.1075	0.0918	426	C	0.0000	0.0000
427	G	0.4725	0.3188	427	C	0.0375	0.0519
428	A	0.6750	0.1576	428	A	0.5175	0.3410
429	A	0.8000	0.2525	429	A	1.4025	0.1533
430	U	0.0525	0.0386	430	G	1.8350	0.1179
431	C	0.0550	0.0640	431	G	0.5175	0.2228
432	U	0.0500	0.1000	432	G	0.0000	0.0000

Supplementary Table 2. Mean SHAPE reactivities of first 432 nts of spliced *env* (AK29) and *sag* (AK30) mRNAs from four experiments. The boxed and yellow highlighted nucleotides show reduced SHAPE reactivities of single stranded purines (ssPurines; from nucleotides 280 to 288) compared to those of unspliced gRNA.
