# Supplementary material for: A purine loop and the primer binding site are critical for the selective encapsidation of mouse mammary tumor virus genomic RNA by Pr77Gag
Source: Nucleic Acids Res. 2021 Apr 9;49(8):4668–88. doi: 10.1093/nar/gkab223 (PMC8096270; doi:10.1093/nar/gkab223)
Supplement: gkab223_Supplemental_Files [file gkab223_supplemental_files.zip › Supplementary Table 3.pdf]

Supplementary Table 3 (pages 1-12)

Nucleotide number & sequence		Mean SHAPE Reactivities (from 4 independent experiments)			
		In the absence of $Pr77^{Gag}$		In the presence of 4uM $Pr77^{Gag}$	
		Mean	SD	Mean	SD
1	G	-999	0	-999	0
2	C	-999	0	-999	0
3	A	-999	0	-999	0
4	A	-999	0	-999	0
5	C	-999	0	-999	0
6	A	-999	0	-999	0
7	G	-999	0	-999	0
8	U	-999	0	-999	0
9	C	-999	0	-999	0
10	C	-999	0	-999	0
11	U	-999	0	-999	0
12	A	-999	0	-999	0
13	A	-999	0	-999	0
14	U	-999	0	-999	0
15	A	-999	0	-999	0
16	U	2.6225	0.5856	-999	0
17	U	1.21	0.3337	-999	0
18	C	0.1075	0.1295	-999	0
19	A	0.29	0.1802	-999	0
20	C	0.0325	0.0525	-999	0
21	G	0.605	0.3712	-999	0
22	U	3.0475	0.9153	-999	0
23	C	0.7675	0.1493	-999	0
24	U	4.5025	1.5269	-999	0
25	C	1.305	0.3112	0.745	0.4834
26	G	0.1825	0.1034	0.075	0.1500
27	U	0.0375	0.0450	0.0375	0.0519
28	G	0.01	0.0141	0.14	0.1635
29	U	0.1275	0.1438	0.2375	0.1340
30	G	0.08	0.0698	0.1375	0.1187
31	U	0.0425	0.0568	0.135	0.0656
32	U	0.1475	0.0330	0.1425	0.0746
33	U	0.075	0.0465	0.075	0.0835
34	G	0.0475	0.0525	0.065	0.0790
35	U	1.95	0.6578	1.21	0.6981
36	G	0.2725	0.1034	0.14	0.1449
37	U	0.0075	0.0150	0.0175	0.0222

Supplementary Table 3 (continued)

Nucleotide number & sequence		Mean SHAPE Reactivities (from 4 independent experiments)			
		In the absence of Pr77^{Gag}		In the presence of 4uM Pr77^{Gag}	
		Mean	SD	Mean	SD
38	C	0.005	0.0100	0	0.0000
39	U	0	0.0000	0.0275	0.0550
40	G	0	0.0000	0	0.0000
41	U	0.0025	0.0050	0	0.0000
42	U	0.245	0.3287	0.2325	0.2794
43	C	0.33	0.1479	0.225	0.3009
44	G	0.005	0.0100	0.0125	0.0189
45	C	0	0.0000	0.1475	0.2950
46	C	0	0.0000	0.6375	0.8320
47	A	2.2375	0.7006	1.6675	0.4861
48	U	1.31	0.4790	0.9625	0.3018
49	C	0.205	0.0911	0.28	0.2110
50	C	0	0.0000	0.065	0.1300
51	C	0	0.0000	0.0775	0.1239
52	G	0.075	0.0772	0.07	0.1400
53	U	0.255	0.1967	0.415	0.1603
54	C	0.6025	0.1097	0.47	0.2258
55	U	0.0175	0.0206	0.02	0.0400
56	C	0	0.0000	0.1075	0.0998
57	C	0	0.0000	0.06	0.1200
58	G	0	0.0000	0	0.0000
59	C	0.0175	0.0350	0.15	0.1152
60	U	0.095	0.1308	0.2525	0.1658
61	C	0.235	0.1515	0.27	0.1036
62	G	0	0.0000	0.0625	0.1121
63	U	0.1175	0.1138	0.4375	0.3707
64	C	0.195	0.3900	3.4375	3.5259
65	A	0.255	0.0968	0.39	0.1023
66	C	0.0025	0.0050	0.0225	0.0450
67	U	0.21	0.1322	0.46	0.1966
68	U	0.32	0.3947	2.5425	2.4904
69	A	0.9275	0.2763	0.4775	0.1443
70	U	0.4175	0.1357	0.2425	0.0574
71	C	0	0.0000	0.3075	0.3354
72	C	0	0.0000	0.1225	0.1415
73	U	0.985	0.2158	0.93	0.2634
74	U	1.6125	0.5619	1.88	0.1780

Supplementary Table 3 (continued)

Nucleotide number & sequence		Mean SHAPE Reactivities (from 4 independent experiments)			
		In the absence of Pr77^{Gag}		In the presence of 4uM Pr77^{Gag}	
		Mean	SD	Mean	SD
75	C	0.045	0.0661	2.5425	2.4623
76	A	1.2225	0.2311	1.18	0.1579
77	C	0.225	0.1542	0.2975	0.0359
78	U	1.3225	0.2439	0.985	0.2844
79	U	1.7775	0.4864	1.19	0.4274
80	U	1.05	0.3473	0.71	0.2824
81	C	0.0225	0.0450	0.185	0.2138
82	C	0.065	0.1300	2.1075	2.2395
83	A	3.105	2.0855	1.92	1.3176
84	G	1.7275	3.2096	0.4725	0.8275
85	A	0.2	0.3611	0.0325	0.0340
86	G	0.0225	0.0450	0	0.0000
87	G	0	0.0000	0.0225	0.0263
88	G	0	0.0000	0.055	0.0640
89	U	0.0175	0.0350	0.0175	0.0287
90	C	0.01	0.0115	0	0.0000
91	C	0.0275	0.0222	0.02	0.0337
92	C	0.1125	0.0900	0.165	0.0645
93	C	0.035	0.0635	0.1875	0.1520
94	C	0	0.0000	0.14	0.2668
95	C	0.0275	0.0550	0.035	0.0700
96	G	0.0575	0.0512	0.01	0.0141
97	C	0	0.0000	0.4325	0.4874
98	A	0.26	0.1208	0.195	0.1256
99	G	0.5425	0.0574	0.295	0.1085
100	A	2.835	0.4871	1.575	0.8021
101	C	0.2475	0.1037	0.1225	0.0737
102	C	0.9425	0.1997	0.6025	0.2253
103	C	1.1525	0.4397	0.7275	0.2926
104	C	0.05	0.0935	0.065	0.0943
105	G	0.0225	0.0450	0.0075	0.0150
106	G	0.12	0.0770	0.0375	0.0189
107	U	0.2325	0.0411	0.15	0.0497
108	G	0.01	0.0200	0.0225	0.0171
109	A	0.0825	0.0624	0.0175	0.0236
110	C	0	0.0000	0.0425	0.0613
111	C	0.3375	0.2427	0.185	0.1282

Supplementary Table 3 (continued)

Nucleotide number & sequence		Mean SHAPE Reactivities (from 4 independent experiments)			
		In the absence of Pr77^{Gag}		In the presence of 4uM Pr77^{Gag}	
		Mean	SD	Mean	SD
112	C	1.595	1.1651	0.9675	0.2834
113	U	6.165	1.6679	4.0375	1.8953
114	C	2.21	0.5687	2.16	0.3789
115	A	7.73	1.6137	4.8175	2.4106
116	G	0.4625	0.2567	0.195	0.1782
117	G	0.0825	0.0806	0.0075	0.0150
118	U	0.085	0.0311	0.085	0.0785
119	C	0.3	0.3369	0.1275	0.2419
120	G	1.3575	1.0648	0.5925	0.1952
121	G	1.66	0.9926	1.2225	0.9326
122	C	0	0.0000	0.065	0.0507
123	C	0.0075	0.0150	0.0775	0.1297
124	G	0.3525	0.1132	0.245	0.0645
125	A	0.705	0.1710	0.26	0.2394
126	C	0	0.0000	0	0.0000
127	U	0.0375	0.0263	0.0225	0.0287
128	G	0.11	0.0594	0.025	0.0300
129	C	0	0.0000	0	0.0000
130	G	0	0.0000	0	0.0000
131	G	0	0.0000	0	0.0000
132	C	0.01	0.0200	0.1175	0.1228
133	A	0.2725	0.3308	1.09	1.0723
134	G	0.4675	0.0556	0.46	0.3692
135	C	2.745	0.7189	1.62	0.6926
136	U	1.2675	0.2919	0.6775	0.4871
137	G	0.325	0.1642	0.045	0.0580
138	G	0.3175	0.2287	0.075	0.0311
139	C	0.14	0.0183	0.115	0.0705
140	G	0.1325	0.0411	0.0425	0.0591
141	C	0.0175	0.0350	0.04	0.0616
142	C	0.0225	0.0450	0.055	0.0656
143	C	0.505	0.2356	0.0875	0.1008
144	G	1.125	0.4014	0.4075	0.2755
145	A	2.51	0.7054	1.23	0.6277
146	A	2.2275	0.3514	1.455	0.3091
147	C	0.0625	0.1250	2.7475	2.3170
148	A	0.8375	0.0978	0.4725	0.1567

Supplementary Table 3 (continued)

Nucleotide number & sequence		Mean SHAPE Reactivities (from 4 independent experiments)			
		In the absence of Pr77^{Gag}		In the presence of 4uM Pr77^{Gag}	
		Mean	SD	Mean	SD
149	G	0.02	0.0245	0.02	0.0245
150	G	0.01	0.0200	0.0075	0.0150
151	G	0	0.0000	0.0575	0.1084
152	A	0.1275	0.2419	0.175	0.3500
153	C	0.0775	0.1484	1.47	1.5422
154	C	0.435	0.5334	1.6975	1.2422
155	C	0.1875	0.2836	0.96	0.4939
156	U	0.1675	0.0350	0.1875	0.1103
157	C	0.455	0.0968	0.28	0.1068
158	G	0	0.0000	0	0.0000
159	G	0	0.0000	0	0.0000
160	A	0.245	0.1678	0.09	0.0762
161	U	1.51	0.5036	0.5875	0.2877
162	A	0.09	0.0942	0.01	0.0200
163	A	0.0775	0.0580	0.0125	0.0189
164	G	0.015	0.0238	0	0.0000
165	U	0.0275	0.0550	0.005	0.0100
166	G	0.08	0.1233	0.02	0.0337
167	A	0.2125	0.2290	0.0525	0.0984
168	C	0.155	0.1792	0.19	0.3668
169	C	0.055	0.0666	0.3175	0.3451
170	C	0.0375	0.0750	0.1075	0.1028
171	U	0.18	0.0698	0.17	0.1230
172	U	0.48	0.1968	0.3875	0.1839
173	G	0.925	0.2671	0.4125	0.3764
174	U	0.6425	0.1550	0.3375	0.1982
175	C	0.005	0.0100	0.03	0.0600
176	U	0	0.0000	0.01	0.0200
177	C	0	0.0000	0	0.0000
178	U	0.1925	0.3850	1.4975	1.8414
179	A	0.3575	0.2734	0.3175	0.2198
180	U	0.125	0.0493	0.13	0.1111
181	U	0.12	0.0716	0.155	0.1401
182	U	0.105	0.1156	0.1075	0.1044
183	C	0.01	0.0200	0.1525	0.2122
184	U	0.2475	0.4685	1.1725	1.1405
185	A	0.1	0.0812	0.1825	0.0888

Supplementary Table 3 (continued)

Nucleotide number & sequence		Mean SHAPE Reactivities (from 4 independent experiments)			
		In the absence of Pr77^{Gag}		In the presence of 4uM Pr77^{Gag}	
		Mean	SD	Mean	SD
186	C	0.07	0.1400	0.2875	0.2363
187	U	0.5525	1.0459	1.9525	1.4806
188	A	0.6725	0.3582	0.5475	0.3411
189	U	0.175	0.0681	0.16	0.1186
190	U	0.07	0.0688	0.09	0.0693
191	U	0.215	0.3129	0.0675	0.1350
192	G	0	0.0000	0.005	0.0100
193	G	0.0375	0.0750	0	0.0000
194	U	0.3275	0.2885	0.175	0.1733
195	G	0.3325	0.2837	0.1275	0.1500
196	U	0.5025	0.2037	0.2225	0.1895
197	U	0.565	0.2640	0.265	0.2659
198	U	0.47	0.2302	0.19	0.1726
199	G	0.035	0.0700	0.0025	0.0050
200	U	0.045	0.0900	0.0125	0.0250
201	C	0	0.0000	0	0.0000
202	U	0.4	0.0726	0.24	0.0775
203	U	0.555	0.2439	0.3775	0.3485
204	G	0.1025	0.2050	0.0775	0.0838
205	U	0.3725	0.3089	1.15	0.9751
206	A	0.295	0.0619	0.165	0.1196
207	U	0.2975	0.1231	0.1525	0.1382
208	U	0.52	0.4262	0.215	0.2307
209	G	0.8975	0.5289	0.485	0.4871
210	U	0.2325	0.1394	0.0975	0.1228
211	C	0	0.0000	0	0.0000
212	U	0.0125	0.0250	0.0125	0.0250
213	C	0.035	0.0700	0	0.0000
214	U	0.0325	0.0650	0.0425	0.0850
215	U	0.165	0.0971	0.0225	0.0263
216	U	0.24	0.1426	0.075	0.0926
217	C	0.0075	0.0150	0.0075	0.0150
218	U	0.37	0.1257	0.2175	0.0746
219	U	0.385	0.1926	0.2125	0.1795
220	G	0.36	0.1720	0.135	0.1121
221	U	0.165	0.1127	0.12	0.0673
222	C	0.0125	0.0189	0.0025	0.0050

Supplementary Table 3 (continued)

Nucleotide number & sequence		Mean SHAPE Reactivities (from 4 independent experiments)			
		In the absence of Pr77^{Gag}		In the presence of 4uM Pr77^{Gag}	
		Mean	SD	Mean	SD
223	U	0.4025	0.1922	0.14	0.1208
224	G	0.375	0.2447	0.12	0.0804
225	G	0.0125	0.0250	0	0.0000
226	C	0	0.0000	0.31	0.3404
227	U	0.67	0.6725	1.735	1.4311
228	A	0.705	0.1482	0.38	0.1534
229	U	0.465	0.1109	0.41	0.1953
230	C	0	0.0000	1.4425	1.1923
231	A	0.625	0.7664	0.5675	0.3468
232	U	0.475	0.3579	0.64	0.3851
233	C	0.0525	0.1050	1.6925	1.4735
234	A	0.61	0.8398	0.6375	0.6736
235	C	0	0.0000	2.235	2.0859
236	A	0.9325	0.4055	0.7525	0.1394
237	A	1.7375	0.0946	1.1825	0.3608
238	G	0.49	0.1734	0.1425	0.0750
239	A	0.1825	0.0263	0.1175	0.0222
240	G	0.0125	0.0126	0.02	0.0183
241	C	0.015	0.0300	0.0925	0.1269
242	G	0.3125	0.2134	0.0725	0.1450
243	G	0.2225	0.0818	0.1	0.1244
244	A	0.105	0.2100	0.09	0.1052
245	A	0.2425	0.1550	0.085	0.0387
246	C	0.185	0.2014	0.085	0.1387
247	G	0.3575	0.2891	0.0375	0.0750
248	G	0.175	0.2079	0.215	0.2669
249	A	5.125	0.8055	2.71	1.3284
250	C	0.0425	0.0613	0	0.0000
251	U	0.075	0.1500	0	0.0000
252	C	0.0225	0.0386	0.0975	0.1127
253	A	0.22	0.1257	0.085	0.1109
254	C	0	0.0000	0.06	0.0712
255	C	0	0.0000	0.57	0.5126
256	A	0.89	1.7800	0.7175	1.3822
257	U	1.3225	0.1601	2.0875	0.8420
258	A	2.19	0.3813	1.2975	0.5881
259	G	0.0425	0.0568	0.02	0.0245

Supplementary Table 3 (continued)

Nucleotide number & sequence		Mean SHAPE Reactivities (from 4 independent experiments)			
		In the absence of Pr77^{Gag}		In the presence of 4uM Pr77^{Gag}	
		Mean	SD	Mean	SD
260	G	0.09	0.1485	0	0.0000
261	G	0.0775	0.0866	0.0325	0.0525
262	A	0.6025	0.2485	0.3275	0.2148
263	G	0.36	0.2765	0.085	0.1377
264	C	0.0275	0.0340	0.015	0.0300
265	U	0.4225	0.2850	0.12	0.1010
266	G	0.295	0.2266	0.08	0.0589
267	C	0	0.0000	2.2475	1.9336
268	A	0.1875	0.3750	0.08	0.1600
269	G	0.4425	0.2900	0.285	0.2111
270	U	0.24	0.0627	0.09	0.1068
271	C	0.08	0.1095	0.2425	0.2417
272	C	0	0.0000	0.205	0.2562
273	C	0	0.0000	0.0675	0.0670
274	G	0	0.0000	0.05	0.0560
275	C	0.24	0.1541	0.355	0.3117
276	C	0.27	0.1203	0.245	0.2424
277	U	0.2125	0.0988	0.1275	0.1258
278	A	0.0875	0.0680	0.0925	0.0512
279	C	0.145	0.0881	0.13	0.1643
280	G	0.275	0.1502	0.1825	0.1617
281	G	1.81	0.4346	0.9	0.1920
282	A	2.595	0.4022	1.505	0.3861
283	G	1.3275	0.2313	0.6075	0.1312
284	A	2.1675	0.3051	1.185	0.4243
285	A	1.4675	0.2367	0.89	0.2255
286	G	1.215	0.1537	0.6325	0.2240
287	A	2.2725	0.4923	1.2125	0.3981
288	G	1.1375	0.1797	0.6475	0.1164
289	G	0.0925	0.1352	0.16	0.1657
290	U	0.0725	0.0780	0.0475	0.0660
291	A	0.0175	0.0287	0	0.0000
292	G	0.0225	0.0450	0.0025	0.0050
293	G	0.13	0.1467	0.0225	0.0330
294	U	0.62	0.3757	0.485	0.3455
295	U	1.3325	0.4555	1.325	0.7713
296	A	1.7375	0.5063	1.29	0.3034

Supplementary Table 3 (continued)

Nucleotide number & sequence		Mean SHAPE Reactivities (from 4 independent experiments)			
		In the absence of Pr77^{Gag}		In the presence of 4uM Pr77^{Gag}	
		Mean	SD	Mean	SD
297	C	1.575	0.4132	1.245	0.3478
298	G	0.1025	0.0574	0.085	0.1226
299	G	0.0925	0.0655	0.0225	0.0222
300	U	0.055	0.0681	0.065	0.0695
301	G	0.0025	0.0050	0.0175	0.0287
302	A	0.1075	0.0830	0.1075	0.0629
303	G	0	0.0000	0.1675	0.2558
304	C	0	0.0000	0.3025	0.3505
305	C	1.1275	1.2550	3.3025	3.0092
306	A	0.7325	0.4670	0.755	0.1603
307	U	1.31	0.4285	0.835	0.1420
308	U	1.485	0.5157	0.7375	0.1452
309	G	0.9275	0.2419	0.3075	0.1396
310	G	0.635	0.0420	0.2275	0.1124
311	A	0.37	0.0455	0.2925	0.2854
312	A	0.2875	0.1103	0.2825	0.2965
313	A	0.32	0.1499	0.3025	0.2562
314	U	0.455	0.1702	0.48	0.0716
315	G	0.0375	0.0263	0.0625	0.0645
316	G	0.0075	0.0150	0	0.0000
317	G	0.02	0.0400	0	0.0000
318	G	0	0.0000	0.035	0.0700
319	G	0.2275	0.0946	0.26	0.1278
320	U	0.8225	0.2496	0.7	0.3175
321	C	0.085	0.0129	0.205	0.2316
322	U	0.24	0.0622	0.215	0.1150
323	C	0.1025	0.0222	0.1175	0.1193
324	G	0.0775	0.0263	0.035	0.0700
325	G	0.1775	0.1377	0.2175	0.0359
326	G	0	0.0000	0.005	0.0058
327	C	0	0.0000	0.0475	0.0585
328	U	0.3175	0.2855	0.6775	0.3635
329	C	2.755	2.0722	5.32	4.5072
330	A	0.865	0.3478	0.77	0.1530
331	A	1.285	0.5089	0.815	0.2117
332	A	1.045	0.3747	0.5725	0.0675
333	A	0.955	0.3114	0.525	0.0985

Supplementary Table 3 (continued)

Nucleotide number & sequence		Mean SHAPE Reactivities (from 4 independent experiments)			
		In the absence of $Pr77^{Gag}$		In the presence of 4uM $Pr77^{Gag}$	
		Mean	SD	Mean	SD
334	G	0.19	0.0876	0.1125	0.0806
335	G	0.0225	0.0386	0.0775	0.0665
336	G	0.03	0.0476	0.3	0.2680
337	C	0.49	0.1948	0.63	0.2636
338	A	0.0575	0.1150	0.17	0.0455
339	G	0.0425	0.0850	0.1375	0.1406
340	A	0.0975	0.0591	0.1675	0.0660
341	A	0.3025	0.1357	0.29	0.1246
342	A	0.0325	0.0403	0.01	0.0141
343	C	0.0625	0.0776	0.04	0.0616
344	U	1.095	0.2596	0.965	0.2613
345	C	0.4425	0.1422	0.3575	0.0793
346	U	1.5675	0.4700	1.0475	0.1601
347	U	1.2975	0.2893	1.0075	0.1670
348	U	1.445	0.3484	1.0525	0.1008
349	G	0.0125	0.0250	0.0325	0.0377
350	U	0.1225	0.1415	0.1425	0.0995
351	U	0.37	0.0392	0.2725	0.0419
352	U	0	0.0000	0.2075	0.3824
353	C	0	0.0000	0	0.0000
354	U	0.35	0.1017	0.4475	0.2879
355	G	4.2575	1.1943	2.93	0.7032
356	U	0.3175	0.1350	0.1775	0.1374
357	U	0.235	0.0404	0.1775	0.0714
358	U	0.365	0.0635	0.315	0.0635
359	U	0.5925	0.5519	1.03	0.9862
360	A	0.6225	0.2330	0.7525	0.2109
361	C	2.1675	2.4387	5.6125	4.7502
362	A	0.315	0.1526	0.3425	0.1727
363	A	0.13	0.0787	0.1475	0.1162
364	A	0.04	0.0283	0.0225	0.0330
365	G	0.0025	0.0050	0	0.0000
366	G	0.0125	0.0250	0.3525	0.6719
367	C	0.0225	0.0450	0.0825	0.0960
368	U	0	0.0000	0.0325	0.0395
369	C	0.0525	0.0806	0.095	0.1109
370	C	0.005	0.0100	0.155	0.2901

Supplementary Table 3 (continued)

Nucleotide number & sequence		Mean SHAPE Reactivities (from 4 independent experiments)			
		In the absence of Pr77^{Gag}		In the presence of 4uM Pr77^{Gag}	
		Mean	SD	Mean	SD
371	U	0.05	0.0469	0.0475	0.0629
372	C	0.07	0.0804	0.255	0.2655
373	U	0.5525	0.1533	0.2825	0.2909
374	C	3.5025	2.6729	2.4225	0.9164
375	A	4.665	1.2064	3.825	0.9533
376	G	3.11	0.9832	2.4075	0.6737
377	A	3.44	1.2007	2.7	0.7877
378	G	0.09	0.1052	0.0775	0.0618
379	A	0.0325	0.0320	0	0.0000
380	G	0	0.0000	0.0175	0.0350
381	G	0	0.0000	0	0.0000
382	G	0	0.0000	0.2125	0.2494
383	G	0	0.0000	0.9725	1.6288
384	U	0	0.0000	0.2375	0.1782
385	C	0	0.0000	0.14	0.1689
386	U	0.11	0.0902	0.0175	0.0236
387	U	1.2425	0.4244	1.1025	0.4847
388	C	2.6025	1.9221	6.9	4.7137
389	A	0.8	0.8568	1.49	0.2660
390	U	0.1275	0.0984	0.2725	0.0608
391	G	0.01	0.0200	0.0375	0.0411
392	U	0.05	0.0627	0.0075	0.0096
393	G	0.0225	0.0450	0	0.0000
394	A	0.0375	0.0556	0.0225	0.0263
395	A	0.1425	0.1130	0.1325	0.1544
396	A	0.52	0.1268	0.4425	0.1544
397	G	0.5575	0.1652	0.4925	0.2004
398	A	0.065	0.0480	0.0475	0.0359
399	G	0.0425	0.0723	0.0075	0.0096
400	A	0.055	0.0656	0.0475	0.0629
401	G	0.02	0.0400	0.11	0.1281
402	U	1.49	2.1801	1.965	1.7148
403	A	0.4625	0.1877	0.455	0.1974
404	G	0.3025	0.0780	0.235	0.1348
405	U	0.34	0.0762	0.3225	0.1081
406	G	1.145	0.1008	1.305	0.2424
407	C	2.085	1.6914	5.94	4.5535

Supplementary Table 3 (continued)

Nucleotide number & sequence		Mean SHAPE Reactivities (from 4 independent experiments)			
		In the absence of Pr77^{Gag}		In the presence of 4uM Pr77^{Gag}	
		Mean	SD	Mean	SD
408	A	0.3525	0.3077	0.63	0.1098
409	A	0.515	0.0520	0.5575	0.1571
410	U	1.0225	0.9828	2.0425	1.9120
411	A	0.46	0.0424	0.4475	0.1417
412	G	0.315	0.2055	0.305	0.1396
413	A	0.4825	0.0486	0.3925	0.1473
414	A	0.535	0.0619	0.4175	0.1078
415	U	0.3125	0.0650	0.295	0.0545
416	U	0.41	0.0779	0.36	0.1294
417	U	0.5175	0.0991	0.495	0.1121
418	U	0.575	0.3695	0.8925	0.7032
419	A	0.5975	0.1537	0.495	0.1863
420	U	0.375	0.1266	0.485	0.0911
421	C	1.4075	1.5318	4.0525	3.0791
422	A	0.28	0.2282	0.47	0.1055
423	G	0.38	0.2652	0.45	0.1822
424	U	0.1925	0.0907	0.215	0.0656
425	U	0.2075	0.0386	0.18	0.0627
426	U	0.0925	0.0250	0.1075	0.1044
427	C	0	0.0000	0.145	0.1150
428	U	0.085	0.1330	1.3875	1.3277
429	A	0.0775	0.0550	0.13	0.0616
430	A	0.3025	0.1147	0.325	0.1797
431	U	0.5925	0.2955	1.6925	1.5118
432	A	0.395	0.1282	0.4	0.1426

Supplementary Table 3. Mean SHAPE reactivities from four experiments in the absence and presence of Pr77^{Gag}. The yellow highlighted nucleotides showed ≥ 1.5 fold reduction of SHAPE reactivities in the presence of Pr77^{Gag} (*p*-value ≤ 0.05). The boxed and highlighted nucleotides (from nucleotides 280 to 288) represent the sequence of single stranded purines (ssPurines).
